# Chromosome-level genome and the identification of sex chromosomes in *Uloborus diversus*

**DOI:** 10.1093/gigascience/giad002

**Published:** 2023-02-10

**Authors:** Jeremiah Miller, Aleksey V Zimin, Andrew Gordus

**Affiliations:** Department of Biology, Johns Hopkins University, Baltimore, MD 21218, USA; Department of Biomedical Engineering, Johns Hopkins University, Baltimore, MD 21218, USA; Center for Computational Biology, Johns Hopkins University, Baltimore, MD 21218, USA; Department of Biology, Johns Hopkins University, Baltimore, MD 21218, USA; Solomon H. Snyder Department of Neuroscience, Johns Hopkins University, Baltimore, MD 21218, USA

**Keywords:** arachnid, spider, genome, Uloborid, cribellate, spidroin

## Abstract

The orb web is a remarkable example of animal architecture that is observed in families of spiders that diverged over 200 million years ago. While several genomes exist for araneid orb-weavers, none exist for other orb-weaving families, hampering efforts to investigate the genetic basis of this complex behavior. Here we present a chromosome-level genome assembly for the cribellate orb-weaving spider *Uloborus diversus*. The assembly reinforces evidence of an ancient arachnid genome duplication and identifies complete open reading frames for every class of spidroin gene, which encode the proteins that are the key structural components of spider silks. We identified the 2 X chromosomes for *U. diversus* and identify candidate sex-determining loci. This chromosome-level assembly will be a valuable resource for evolutionary research into the origins of orb-weaving, spidroin evolution, chromosomal rearrangement, and chromosomal sex determination in spiders.

## Background

Spiders are among the most successful and diverse terrestrial predators on Earth. Almost 400 million years of evolution has produced more than 50,000 extant spider species representing 128 families that are distributed over every continent except Antarctica [1]. The success of these animals is due in part to the diversity of behaviors that have evolved to capture prey in different environments [2]. Many spiders attack their prey by physically grabbing and immobilizing them with venom and use their silk exclusively for egg sacs. Others use silk to line their burrows or construct webs of varying geometry and composition to detect or entrap prey. The diversity of web use correlates with a diversity of spidroin proteins that form silk, as well as the glands that produce these proteins [2–6]. Spiders such as orb-weavers alternate between glands depending upon the web feature they are constructing. For example, load-bearing parts of the web such as the radii are composed of major ampullate silk that has high tensile strength, whereas the anchors are made up of pyriform silk, which is sticky and amorphous [5].

**Figure 1:**
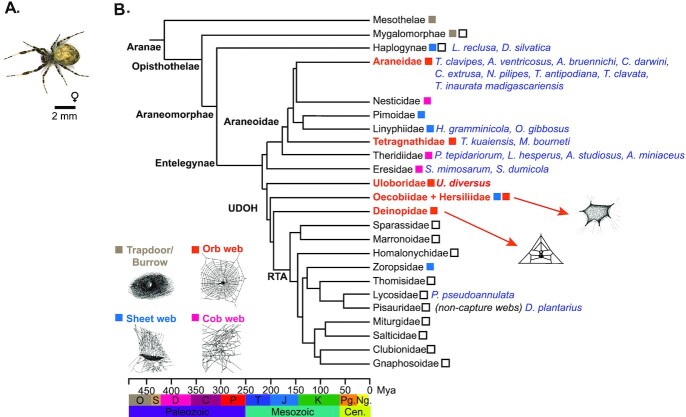
Spider phylogeny. (A) A female *U. diversus*. (B) Phylogeny of spiders. Orb-weaver families are highlighted in orange. Species with sequenced genomes are highlighted in blue. *U. diversus* is highlighted in red. Example webs from Roberson [45], Glatz [46], and Coddington [18]. Divergence times were obtained from Fernández et al. [7]. UDOH, Uloboridae, Deinopidae, Oecobiidae, Hersiliidae; RTA, retrolateral tibial apophysis; O, Ordovician; S, Silurian; D, Devonian; C, Carboniferous; P, Permian; T, Triassic; J, Jurassic; K, Cretaceous; Pg., Paleogene; Ng., Neogene; Mya, millions of years ago.

Remarkably, the orb web is not restricted to a single monophyletic group but is observed in 2 lineages that diverged 250 million years ago, leading to considerable debate about its evolutionary origins [3, 7–9] (Fig. 1). Araneoidea is the largest superfamily of orb-weavers, which have evolved adhesive aggregate spidroins that are used in the capture spiral to adhere prey to the web [4, 10–13]. However, uloborids also build orb webs but use a more ancient cribellate spidroin in their capture spiral to immobilize prey [14–17]. In addition to Uloboridae, other families such as Deinopidae and Oecobiidae + Hersiliidae (Uloboridae, Deinopidae, Oecobiidae, Hersiliidae [UDOH] grade in Fig. 1) also build orb webs, but with more derived behavioral and structural characteristics [18]. Orb-weaving is an innate behavior, with discrete stages of web construction that are shared between araneoid and nonaraneoid orb-weavers [19]. When exposed to neuroactive compounds, the behaviors within specific stages are altered, indicating that the neuronal targets of these compounds are more important for certain stages than others [20–22]. This behavioral paradigm offers an excellent model for understanding not only how complex behaviors can be organized in a small brain [23] but also how such behaviors evolve.

However, a genetic understanding of the evolution of orb-weaving behavior is hampered by a lack of sequenced genomes for nonaraneoid families. Spider genomes are enormous with high repeat content, making them challenging to assemble [24–26]. While 22 spider genomes have been assembled and made publicly available [24, 27–42] (Supplementary Table S1), most are highly fragmented, with 7 assembled to chromosome scale [29, 32, 35, 37, 38, 41, 42]. Of the 22 genomes, only 7 represent the Araneoidea [28, 29, 32, 39] or ecribellate orb-weavers, 2 of which have been assembled to chromosome scale [29, 32]. While Correa-Garhwal et al. [43] recently published a scaffold-scale 10× assembly of the *Uloborus diversus* genome, we provide the first chromosome-scale assembly for a member of the UDOH clade. In addition, to date, only 1 genome represents a member of the cribellate retrolateral tibial apophysis (RTA) clade, which is the sister clade to the UDOH clade (Fig. 1B) [44]. Chromosome-scale assemblies are essential for understanding evolutionary divergence and identifying sites of chromosomal reorganization that play roles in adaptation and speciation.

Spiders have multiple sex chromosomes, with ♂X_1_X_2_/♀X_1_X_1_X_2_X_2_ being the most common sex determination observed. Sex chromosome dosage compensation has evolved multiple times, but all known genetic mechanisms are for single sex chromosome systems. A molecular understanding of dosage compensation in spiders is lacking, in part due to a paucity of sex-associated genetic loci.

*U. diversus* (NCBI: txid327109) (Fig. 1A), of the UDOH clade, is a cribellate orb-weaving spider native to the desert Southwest in the United States [47] and an important model for understanding the evolution of spidroins and orb-weaving [44]. The existence of a nonaraneoid orb-weaving genome is crucial for addressing the evolutionary origins of orb-weaving. Recent work has demonstrated the utility of this species as a model system for understanding orb-weaving behavior [23]. This, combined with the potential to compare both behaviors and their genetic underpinnings across divergent species of orb-weavers, offers a rich opportunity to understand the underlying genetics that encode this behavior and whether orb-weaving behaviors are conserved or convergently evolved. Here, we report a high-quality, chromosome-scale draft genome assembly of *U. diversus* (NCBI: txid327109), as well as a complementary transcriptome assembly and gene annotations. This genome enabled the identification of full >10-kb spidroin genes, as well as the identification of sex chromosomes for this species. This chromosome-level assembly will be a valuable resource for evolutionary research into the origins of orb-weaving, spidroin evolution, chromosomal rearrangement, and chromosomal sex determination in spiders.

## Data Description

### Genome sequencing

We sequenced and assembled a high-quality, chromosome-scale genome assembly for *U. diversus* using a hybrid approach that leveraged the complementary benefits of multiple technologies. The genome of *U. diversus* contains long regions of a low-complexity sequence, which hinders assembly using short reads alone, as well as extremely long protein-coding genes, which makes long reads necessary for a reference-quality assembly [24–29]. Illumina sequencing provides high sequence fidelity but short read lengths, while ONT sequencing provides long read lengths, useful for scaffolding and spanning long, low-complexity regions but lower sequence fidelity [48]. PacBio HiFi sequencing provides an excellent combination of long read lengths and high sequence fidelity, but we were able to produce multiple megabase-long reads with ONT, which is not possible with PacBio. Each of these sequencing technologies provided unique advantages for improving the overall assembly.

To limit genetic variation, we used sequencing data from only 5 unmated female spiders in our assembly. We used Illumina to obtain high fidelity read data from a single female spider, generating 795 million 150-bp read pairs totaling 119.3 Gb. Because Illumina short reads are not sufficiently long to span long, highly repetitive regions encountered in spider genomes, we sequenced 3 ONT libraries, each from a single female, generating 14.7 million reads totaling 98.4 Gb, with a read N50 of 6.7 kb. To obtain long sequencing reads with high sequence fidelity, we also sequenced a single adult female using PacBio HiFi, generating 35 million subreads totaling 412.8 Gb with a read N50 of 12.8 kb, which yielded 2 million consensus reads totaling 26 Gb with a read N50 of 13.0 kb. To investigate the sex determination system in *U. diversus*, we also generated an Illumina library from a single adult male, producing 937 million 50-bp read pairs totaling 46.8 Gb. Sequencing library statistics are available in Table 1.

**Table 1:** Summary of sequencing library statistics

Library type	Instrument	Mean read length	Number of reads or read pairs	Bases sequenced	Coverage (×)^a^
Illumina	NovaSeq 6000	2 × 150 bp	795 million (female)	119.3 Gb (female)	60 (female)
			937 million (male)	46.8 Gb (male)	24 (male)
ONT	PromethION	6.7 kb	14.7 million	98.4 Gb	50
PacBio	Sequel II	12.8 kb (subreads)	34.9 million (subreads)	412.8 Gb (subreads)	208 (subreads)
		13.0 kb (consensus)	2.0 million (consensus)	26.2 Gb (consensus)	13.1 (consensus)
Chicago Hi-C	HiSeq X	2 × 150 bp	286 million	85.8 Gb	21b 43c
Dovetail Hi-C	HiSeq X	2 × 150 bp	537 million	161.1 Gb	1,009b 81c

a Based on in silico genome size estimate of 1.98 Gb by *k*-mer analysis with GenomeScope 2.0.

b Physical coverage, defined as the number of read pairs that span a base pair.

c Sequence coverage, defined as number of times a base pair is directly observed in sequencing data.

### Genome size, heterozygosity, and coverage estimation

**Figure 2:**
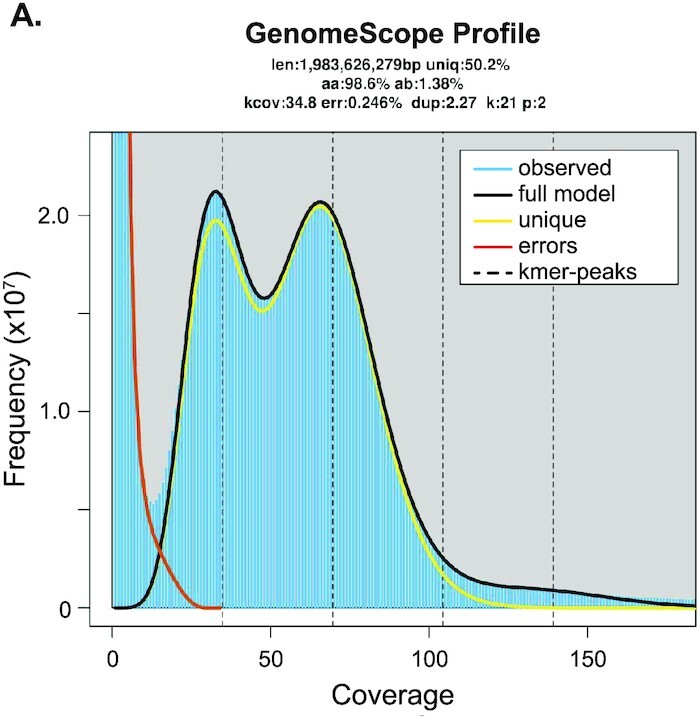
GenomeScope plot from Illumina data. The *k*-mer spectra for Illumina reads from a single, virgin female. The diploid and haploid peaks are at 70× and 35× coverage, respectively.

To assess the size and heterozygosity of the genome, we used Jellyfish [49] to count the frequency of canonical 21-mers in our adult female Illumina sequencing reads and used the 21-mer distribution as input to GenomeScope [50]. The resulting model estimated a genome size of 1.98 Gb with a heterozygosity of 1.38% and 50.2% of the genome occurring as a unique sequence (Fig. 2), similar to other spider genomes (Supplementary Table S1) [24, 27–34]. Given this genome size, our Illumina sequencing yielded 63× coverage, our ONT sequencing yielded 68× coverage, and our PacBio HiFi yielded 208× in raw read coverage and 13× in consensus read coverage (Table 1).

### Karyotype of *U. diversus*

**Figure 3:**
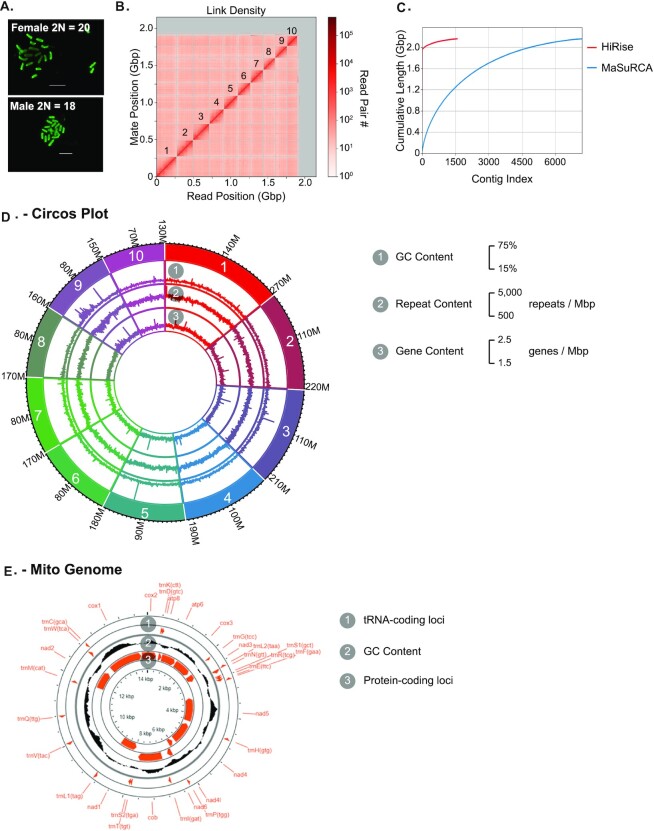
Chromosome-scale genome assembly. (A) Karyotype of female and male embryos. Female and male diploid sizes of 20 and 18, respectively, indicate a ♂X1 × 2/♀X1 × 1 × 2 × 2 sex determination system, with 8 autosomes. (B) Hi-C linkage map of assembled scaffolds. The 10 largest scaffolds are annotated. (C) Comparison of HiRise and MaSuRCA assemblies. The majority of the HiRise assembly is captured by the first 10 scaffolds. (D) Circos plot of 10 largest nuclear scaffolds, highlighting GC content, repeat content, and gene content across the scaffolds. (E) Circos plot of mitochondrial scaffold, highlighting transfer RNA-coding loci, protein-coding loci, and GC content.

To infer the expected number of pseudo-chromosomes in our final assembly, we determined the number of chromosomes in *U. diversus* using metaphase karyotyping. Mitotic chromosome spreads from developing embryos displayed 2 distinct patterns of chromosome number: either 18 or 20 (Fig. 3A), consistent with ♂X_1_X_2_/♀X_1_X_1_X_2_X_2_ sex determination, which is the most common form of sex determination observed in spiders [51]. Thus, *U. diversus* appears to have 8 autosomes and 2 sex chromosomes.

### *De novo* nuclear genome assembly

First, we used MaSuRCA [52, 53] to produce an initial assembly (*U. diversus* v.1.0) using Illumina short-read data scaffolded by ONT long-read data, consisting of 68,259 scaffolds spanning 3.22 Gb. The scaffold N50 was 98,014 bp and the scaffold L50 was 6,558 (Table 2), with 94.7% of complete BUSCOs (Table 3). The inferred redundancy accounts for the significant increase in the length of the assembly compared to the expected genome size. High heterozygosity leads to alternative haplotypes that can often be misassembled into their own contigs.

**Table 2:** Summary of *Uloborus diversus* draft genome assembly statistics

	*U. diversus*v.1.0		*U. diversus*v.1.1		*U*. *diversus* v.1.2		*U. diversus*v.1.3		*U. diversus*v.2.0		*U. diversus*v.3.0		*U. div v.3.1*	
	Contigs	Scaffolds	Contigs	Scaffolds	Contigs	Scaffolds	Contigs	Scaffolds	Contigs	Scaffolds	Contigs	Scaffolds	Contigs	Scaffolds
Total length	3,218,774,062		3,218,805,127		2,798,889,957		2,802,061,857		2,122,354,655		2,151,304,433		2,151,890,133	
Number of contigs/scaffolds	68,581	68,259	68,581	66,417	50,738	48,632	55,142	21,317	9,997	9,734	7,467	7,197	7,713	1,586
Largest contig/scaffold	3,987,394	3,987,394	3,987,394	6,446,995	3,987,394	6,446,995	3,987,394	367,325,988	4,617,239	4,617,239	5,877,357	5,877,357	5,877,357	272,330,431
Mean contig/scaffold length	46,933	47,155	46,933	48,463	55,162	57,552	50,756	131,447	212,298	218,035	288,107	298,916	278,918	1,356,803
Median contig/scaffold length	21,841	21,923	21,841	21,546	23,184	22,648	24,516	12,994	137,429	218,035	166,676	175,879	168,537	94,191
Smallest contig/scaffold	1,106	1,667	1,106	1,667	1,106	1,667	10	312	10,045	10,045	10,045	10,045	380	10,220
N10/L10	558,595/374	564,850/373	558,595/374	686,213/308	613,610/302	744,166/249	432,729/4,437	367,325,988/1	1,100,324/132	1,100,324/132	1,705,719/86	1,715,742/84	1,564,267/91	272,330,431/1
N20/L 20	330,632/1,135	332,714/1,131	330,632/1,135	395,570/943	375,534/894	452,633/744	271,565/1,278	289,477,137/2	711,683/383	713,631/382	1,147,222/244	1,163,865/241	1,032,642/237	221,850,441/2
N30/L30	214,753/2,358	215,989/2,348	214,753/2,358	252,183/1,983	252,860/1,816	296,524/1,521	192,451/2,518	264,078,423/3	516,155/741	518,859/736	826,976/467	844,177/461	763,983/509	218,885,758/3
N40/L40	114,360/4,189	145,056/4,171	114,360/4,189	166,010/3,572	178,542/3,143	205,473/2,661	139,664/4,234	243,450,885/4	401,815/1,210	404,285/1,205	636,610/763	648,167/753	587,015/831	185,519,777/4
N50/L50	97,524/6,913	98,015/6,885	97,524/6,913	108,431/5,994	126,236/5,019	142,390/4,302	103,046/6,850	241,033,576/5	326,127/1,798	328,082/1,789	487,746/1,150	496,769/1,133	452,781/1,250	185,519,777/5
N60/L60	64,898/10,992	65,255/10,942	64,898/10,992	69,185/9,724	88,433/7,678	97,899/6,679	75,059/9,775	217,901,867/7	259,595/2,527	261,845/2,511	380,517/1,651	387,963/1,626	359,368/1,784	172,099,698/7
N70/L70	42,949/17,131	43,310/17,041	42,949/17,131	44,720/15,580	60,079/11,529	64,377/10,226	53,001/14,221	213,666,783/8	201,138/3,460	204,029/3,433	292,644/2,296	298,089/2,258	280,097/2,463	161,310,338/8
N80/L80	28,594/26,353	28,760/26,208	28,594/26,353	29,173/24,561	38,159/17,400	39,689/15,782	35,344/20,692	183,225,947/9	148,105/4,686	151,467/4,638	206,980/3,169	211,349/3,116	200,858/3,369	159,483,530/9
N90/L90	18,127/40,511	18,198/40,294	18,127/40,511	18,295/38,527	21,027/27,290	21,374/25,391	20,312/31,107	37,840/1,399	97,979/6,436	100,002/6,354	127,987/4,484	131,815/4,398	126,330/4,713	9,355,840/14
N100/L100	1,106/68,581	1,667/68,259	1,106/68,581	1,667/66,417	1,106/50,738	1,667/48,632	10/55142	312/21,317	10,045/9,997	10,045/9,734	10,045/7,467	10,045/7,197	380/7,713	10,220/1,586
Gaps	322		2,164		2,106		33,825		263		270		6,127	
Ns	32,200		63,265		57,963		3,229,863		6,049		6,749		592,449	
GC content (%)	33.78		33.78		33.72		33.72		33.83		33.82		33.82	

*U. diversus* v.1.0 is the MaSuRCA assembly.

*U. diversus* v.1.1 is the MaSuRCA assembly with further scaffolding using Rascaf.

*U. diversus* v.1.2 is the MaSuRCA assembly with Rascaf and with reduction of redundancy using Pseudohaploid.

*U. diversus* v.1.3 is the MaSuRCA assembly with Rascaf and Pseudohaploid further scaffolded using Chicago and Dovetail Hi-C.

*U. diversus* v.2.0 is the PacBio IPA assembly.

*U. diversus* v.3.0 is the MaSuRCA assembly and PacBio IPA assembly merged using MaSuRCA.

*U. diversus* v.3.1 is the merged MaSuRCA/IPA assembly further scaffolded using Dovetail Hi-C.

**Table 3:** Summary of draft genome assembly BUSCO scores

	*v.1.0*	*v.1.1*	*v.1.2*	*v.1.3*	*v.2.0*	*v.3.0*	*v.3.1*
Complete	94.7	95.4	95.1	97.0	92.4	94.1	94.8
Single copy	70.9	72.9	78.4	84.6	82.1	82.9	85.2
Duplicated	23.8	22.5	16.7	12.4	10.3	11.2	9.6
Fragmented	2.3	1.9	2.0	1.2	1.7	1.3	1.0
Missing	3.0	2.7	2.9	1.8	5.9	4.6	4.2

Next, we used Rascaf [54] to improve continuity and ordering of scaffolds in the initial MaSuRCA assembly. Rascaf uses paired-end RNA sequencing (RNA-seq) reads to improve the contiguity of gene models and scaffolds. We observed a modest improvement, reducing the number of scaffolds from 68,259 to 63,265, with the scaffold N50 increasing from 98,014 bp to 108,431 bp and the scaffold L50 decreasing from 6,885 to 5,994, with no change in the assembly span (Table 2). However, despite identifying 95.4% of BUSCOs, 22.5% were duplicated (Table 3). This, combined with the large span of the genome, indicated a high degree of redundancy in the assembly.

To filter out redundant heterozygous contigs, we used Pseudohaploid [55]. Pseudohaploid filters suspected homologous contigs and selects a single representative contig where high rates of heterozygosity prevent assemblers from appropriately identifying haplotypes. We reduced the number of scaffolds by 23.1%, with an increase in scaffold N50 and a decreased span from 3.22 to 2.8 Gb (Table 2) and a drop in duplicated BUSCOs to 16.7% (Table 3). This suggests that Pseudohaploid was able to accurately collapse much of the redundant sequence attributable to alternative haplotypes.

To further improve our assembly, we sequenced using PacBio HiFi and assembled the resulting HiFi reads with PacBio's IPA (Improved Phased Assembly) pipeline, resulting in a substantial decrease in the new assembly span, from 2.8 to only 2.1 Gbp. The number of scaffolds in this assembly was only 9,734, a remarkable improvement. The scaffold N50 in the IPA assembly was 328,082 bp and the scaffold L50 in the IPA assembly was 1,789 (Table 2). The BUSCO score for the IPA assembly indicated that this assembly contained 92.3% of BUSCOs complete (83.7% in single copy, 9.3% duplicated) (Table 3).

We then used SAMBA [56] to merge the previous MaSuRCA assembly with the IPA assembly. SAMBA used scaffolds from the MaSuRCA assembly produced with Illumina and ONT data to patch gaps in the scaffolds of the IPA assembly produced with PacBio HiFi data. Overall, the gap-closing process patched 2,617 gaps, inserting 31.4 Mbp of sequence, accounting for only about 1.5% of sequence added to the HIFI assembly. The new assembly had a length of 2.1 Gbp from only 7,197 scaffolds, with a scaffold N50 of 496,769 bp (Table 2) and 94% complete BUSCOs (Table 3).

To generate a chromosome-level assembly, we used HiRise to scaffold the IPA + MaSuRCA assembly with a Dovetail Hi-C library [57]. Scaffolding did not change the amount of sequencing in the assembly; however, the total number of scaffolds was reduced by 78% to only 1,586 scaffolds, with a remarkable improvement in scaffold N50, which increased to 185,519,777 bp in the Hi-C scaffolded assembly (Table 2, Fig. 3C). Most important, 88% of the total assembly was represented by 10 large scaffolds that comprise 1.9 Gbp (Fig. 3B), matching the expected number of chromosomes (Fig. 3A). The BUSCO score for the final assembly showed 94.8% of the BUSCOs were complete (with 85.2% in single copy, 9.6% duplicated) (Table 3). Our final chromosome-level genome assembly statistics are consistent with previously published spider genomes, with 10 scaffolds representing the 10 chromosomes and high-scaffold N50 [24, 27–34] (Supplementary Table S1). We will refer to these 10 scaffolds as pseudochromosomes.

### Repeat annotation

To characterize repetitive sequences, we constructed a species-specific repeat library using RepeatModeler2 [58]. This library was used in conjunction with the RepBase RepeatMasker Edition [59] database for masking the genome. RepeatMasker analysis of the combined *U. diversus* and RepBase repeats masked 66.6% of the final *U. diversus* genome assembly. Many (29.27%) of the repetitive regions were unclassified; however, DNA transposons accounted for a similar proportion (22.73%). Retroelements accounted for a much smaller proportion (7.7%). Total interspersed repeats account for 59.7% and simple repeats cover 2.61% of the genome (Table 4). The disparity between the GenomeScope estimation of 49.8% repetitive sequence and the RepeatMasker estimation of 66.6% suggests that the repeat content may be underestimated by GenomeScope. Therefore, the genome size may also be underestimated by GenomeScope. Because the length of the *U. diversus* genome is slightly less than the prediction by GenomeScope, this possibility suggests that the length of the assembly may more closely represent the true length of the *U. diversus* genome than the GenomeScope prediction. The repeat content is typical of spider genomes (Supplementary Table S2).

**Table 4:** Summary of the repeat content of the *Uloborus diversus* draft genome assembly

Type of element	Number of elements	Total length	Percent of assembly
Retroelements	311,947	165,713,554	7.70
SINEs	87,037	46,791,087	2.17
Penelope	29,911	11,105,124	0.52
LINEs	151,953	66,189,386	3.08
CRE/SLAC	0	0	0.00
L2/CR1/Rex	31,294	18,030,662	0.84
R1/LOA/Jockey	17,662	9,587,620	0.45
R2/R4/NeSL	0	0	0.00
RTE/Bov-B	40,080	15,062,698	0.70
L1/CIN4	21,465	5,682,132	0.26
LTR elements	72,957	52,733,081	2.45
BEL/Pao	10,210	8,094,671	0.38
Ty1/Copia	24,043	10,565,700	0.49
Gypsy/DIRS1	27,837	29,214,338	1.36
Retroviral	10,867	4,858,372	0.23
DNA transposons	1,510,089	489,113,225	22.73
hobo-Activator	545,025	164,842,101	7.66
Tc1-IS630-Pogo	428,233	150,644,883	7.00
En-Spm	0	0	0.00
MuDR-IS905	0	0	0.00
PiggyBac	11,411	4,281,311	0.20
Tourist/Harbinger	6,573	2,466,806	0.11
Other (Mirage, P-element, Transib)	2,942	1,822,173	0.08
Rolling-circles	229,864	88,460,474	4.11
Unclassified	2,482,847	629,886,344	29.27
Total interspersed repeats		1,284,713,123	59.70
Small RNA	20,797	5,420,377	0.25
Satellites	0	0	0.00
Simple repeats	529,329	56,254,893	2.61
Low complexity	67,980	3,208,338	0.15
**Total**			66.58

### Transcriptome sequencing and assembly

To identify protein-coding genes, we assembled a transcriptome. To capture a wide range of transcripts, we extracted RNA from spiders at multiple developmental stages and from male and female adults. We produced an Illumina short-read sequencing library from a whole adult female, a whole adult male, the dissected prosoma (cephalothorax) from an adult female, the dissected opisthosoma (abdomen) from the same adult female, the dissected prosoma from an adult male, the dissected opisthosoma from the same adult male, the pooled legs from the dissected male and female, a single fourth instar female, and approximately 30 pooled second instars. In total, 302 million read pairs were generated, totaling 45.4 Gbp.

We used Trinity [60] to assemble a genome-guided transcriptome. We then used TransDecoder [60] to find coding regions within our transcripts. We included homology searches to known proteins using both BLAST [61, 62] and Pfam [63] searches. We assessed the BUSCO score of the long open reading frames (ORFs) predicted by TransDecoder, finding that 90.9% of the BUSCOs were present and complete, with 54.8% single copy and 36.1% duplicated, with 1.5% present but fragmented and 7.6% missing (Table 3).

### Protein-coding gene annotation

For protein coding gene annotations, we used BRAKER2 [64, 65] with our RNA-seq data and homology evidence using a custom library of spider proteins obtained from NCBI (Supplementary Table S3). The number of predicted genes in the final *U. diversus* assembly was 44,408, with 40,466 models predicted on the 10 pseudochromosomes (Table 5), with 86.7% of complete BUSCOs. To functionally annotate these genes, we used Interproscan [66, 67] to annotate the longest CDS for each gene. In total, 30,911 models were assigned a domain or function from 1 or more of the databases used (Table 6).

**Table 5:** Summary of annotation statistics for the *Uloborus diversus* draft genome assembly

Attribute	Value
Number of gene models	45,762
Minimum gene model length (bp)	60
Maximum gene model length (bp)	408,348
Average gene model length (bp)	16,764
Number of exons	222,483
Average number of exons per gene model	5
Average exon length (bp)	237
Number of transcripts	47,540
Average number of transcripts per gene model	1
Number of gene models <200 bp	37

**Table 6:** Summary of Interproscan results

Database	Total hits	Individual mRNAs with hits
CDD	11,047	6,560
Coils	9,254	5,947
Gene3D	34,377	16,424
Hamap	270	260
MobiDBLite	44,028	14,046
PANTHER	46,497	20,941
Pfam	35,843	19,491
PIRSF	916	736
PRINTS	16,380	3,134
ProSitePatterns	8,222	3,898
ProSiteProfiles	23,252	9,922
SFLD	120	62
SMART	25,638	7,561
SUPERFAMILY	26,766	15,559
TIGRFAM	821	736

### Noncoding RNA annotation

We used tRNAscan-SE [68, 69] to annotate transfer RNAs (tRNAs). We found 3,084 tRNAs coding for the standard 20 amino acids and 14 tRNAs coding for selenocysteine tRNAs. We found 21 tRNAs with undetermined or unknown isotypes, 537 tRNAs with mismatched isotypes, and 57,824 putative tRNA pseudogenes. We identified no putative suppressor tRNAs. We used Barrnap [70] to annotate ribosomal RNAs (rRNAs). We found 114 rRNAs, of which 100 were located on the 10 pseudochromosomes. These included 6 copies of the 18S subunit, with 4 on pseudochromosomes; 83 copies of the 5S subunit, with 81 on pseudochromosomes; 6 copies of the 5.8S subunit, with 2 on pseudochromosomes; and 19 copies of the 28S subunit, with 13 pseudochromosomes.

### Mitogenome assembly

Animal mitochondrial genomes comprise 37 genes: 13 protein-coding genes, 22 tRNAs, 2 rRNAs, and at least 1 control region [71]. We assembled the mitochondrial genome sequence with NOVOplasty [72] using the adult female Illumina DNA sequencing data and each of the mitochondrial genome sequences listed in Supplementary Table S4 as sources for seed sequences [73–86]. Each run produced the same single, circularized 14,737-bp mitochondrial sequence, consistent with the expected size for an arachnid mitochondrial sequence [71]. We annotated the sequence with the MITOS2 web server [87] and found all 13 of the expected protein-coding genes, 20 of 22 tRNAs, 2 rRNAs, and the control region (Fig. 3E). All identified tRNAs were truncated and lacked T-arms, which has been observed in other species [78, 80, 88, 89]. The functional reason for these truncated tRNAs is unknown, but posttranscriptional RNA editing is assumed to be necessary to recover functional 3′ aminoacyl acceptor stems. Shrunken tRNAs are observed in other metazoans such as *Caenorhabditis elegans*, which has an extended mt EF-Tu1 that can bind to T-arm-lacking tRNA and deliver it to the ribosome [90]. Why these truncations evolve is currently unknown.

### Identification and analysis of spidroins

Spidroins are a unique class of proteins that are the primary components of spider silk. While all spiders produce silk, spidroins have evolved for different uses in web-making. Orb-weavers in particular evolved several silk glands that each produce a different repertoire of spidroins to make different silks with varying utility. Several ecribellate Araneidae spidroins have been sequenced, and many of these spidroins are also made by cribellate orb-weavers such as *U. diversus*. However, ecribellate spiders evolved a unique type of hydrated flageliform silk for their capture spiral, whereas cribellate spiders such as *U. diversus* use a dry cribellate silk in their capture spirals.

Spider dragline silk has the strongest stress and strain capabilities of any known substance. Interest in silk properties extends beyond their evolved use, as silk has many potential human applications in both industry and medicine [91–98]. However, the genetic characterization of spidroins is often challenging due to their exceptional length (coding regions >5 kb) and high repeat content [25]. The annotation of these genes is difficult and often fragmented because reads rarely span the entire length of these genes. With the exceptional contiguity and read depth of our assembly, due to the diversity of sequencing technologies employed, we identified the entire open-read frames of all major spidroins in the *U. diversus* genome.

We found 12 full-length candidate sequences, including at least 1 candidate for each of the 7 types of spidroin used by cribellate orb-weavers, as well as candidate sequences for the ampullate spidroin (AmSp) and 2 proposed paracribellar spidroins (Sp_vA and Sp_vB) recently reported by Correa-Garhwal et al. [43]. There were no gaps in the assembly interrupting our candidate spidroin sequences with the exception of Sp_vA. We performed read mapping to validate the continuity of each full-length sequence and ensure that the predicted sequences were not chimeric. In 10 cases, including the 3 minor spidroin (MiSp) candidates, a major spidroin (MaSp) MaSp-1 candidate, both MaSp-2 candidates, a tubuliform spidroin (TuSp) candidate, an aciniform spidroin (AcSp) candidate, an AmSp candidate, and 1 paracribellar spidroin candidate (Sp_vB), the full length of the predicted genomic region was spanned entirely by at least 1 HiFi consensus read. In the remaining cases, consisting of a pyriform spidroin (PySp) candidate, a cribellate spidroin CrSp candidate, a pseudoflagelliform spidroin (Pflag) candidate, and a paracribellar spidroin candidate (Sp_vA), no more than 2 HiFi reads were necessary to span the entirety of the predicted genomic region, and in each of these cases, there was sufficient depth and overlap in the reads to call the region with high confidence (see Supplementary Fig. S1). The length of the coding regions for the spidroins ranged from 5.5 to 20 kb. This is consistent with expectations of full-length sequences found in other spiders [27, 28, 99]. Only in the cases of MaSp-1 and Sp_vA were we unable to call a complete, full-length sequence. All spidroins other than MaSp-1 and Sp_vA were found to be single-exon sequences. It is likely that Sp_vA would be identified as a single-exon sequence if the gap in the assembly were to be resolved, consistent with that found by Correa-Garhwal et al. While most single-exon genes tend to be small highly expressed proteins such as histones, spidroins are a rare exception. The single-exon structure of spidroin genes has been noted in other species, as well as most recently in *U. diversus* [26, 43, 99–106].

#### Aciniform spidroin (AcSp)

**Figure 4:**
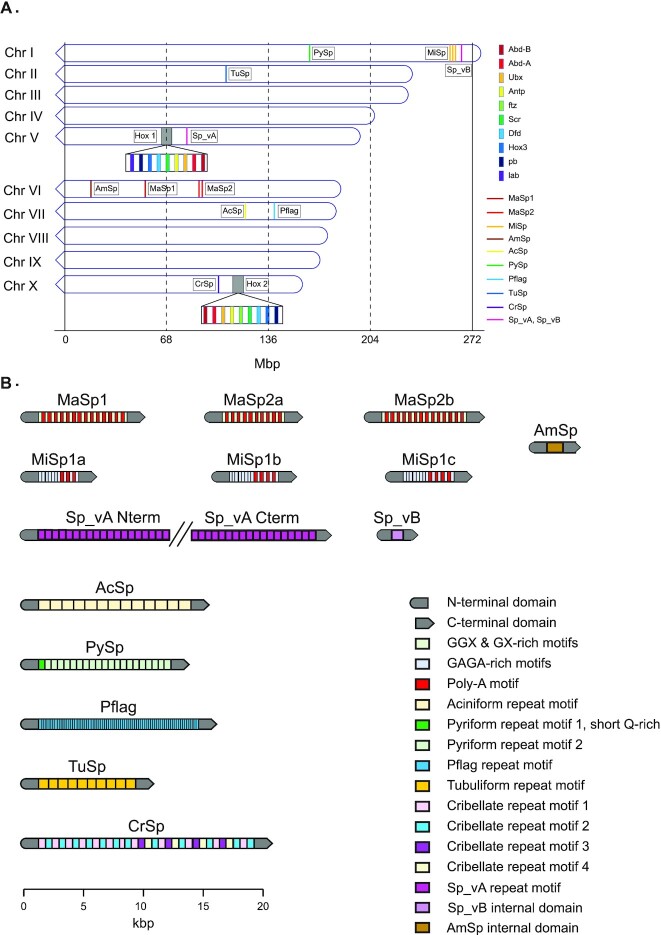
Gene annotations. (A) Gene loci for spidroins and *hox* gene clusters. (B) Domain composition of identified spidroins. (Repeat region annotations are condensed for clarity.)

Aciniform silk is one of the toughest spider silks and is typically used for wrapping prey [107]. A single exon for AcSp was identified on chromosome 7 (Fig. 4A and Table 7), consistent with the sequences in the araneid orb-weaving spiders *Araneus ventricosus* [108] and *Argiope agentata* [109], as well as the cobweb spider *Latrodectus hesperus* [26]. Our confidence in this sequence is high since the complete sequence was spanned entirely by multiple PacBio HiFi reads. In the repetitive region, we found 13 iterated repeats of a 357–amino acid motif and a 14th partial repeat (Supplementary Fig. S1). This differs somewhat from the results reported by Correa-Garhwal et al. [43], which identified 10 tandem repeats. As with previous reports on the structure of AcSp [26, 108–110], we also found that the repeats are remarkably well homogenized. After removal of the signal peptide between Ser-23 and Arg-24, the remaining N-terminal domain secondary structure includes 5 alpha-helices and a C-terminal domain consisting of 4 alpha helices, which is consistent with the structure found in other AcSps [108].

**Table 7:** Summary of spidroins

Spidroin	Gene length (bp)	CDS length (bp)	Protein length (aa)	N-terminal length (aa)	C-terminal length (aa)	Signal peptide stop
Aciniform	14,907	14,907	4,968	148	109	Ser-23
Pseudoflagelliform	15,549	15,549	5,182	195	96	Gly-29
Cribellate	20,198	20,115	6,704	874	296	Gly-23
Major ampullate 2a	7,317	7,317	2,438	165	100	Gly-25
Major ampullate 2b	9,141	9,141	3,046	195	109	Gly-25
Minor ampullate 1a	5,523	5,523	1,840	242	95	Gly-23
Minor ampullate 2a	6,255	6,255	2,084	254	99	Gly-23
Minor ampullate 2b	6,432	6,432	2,143	254	98	Gly-23
Pyriform	13,179	13,179	4,392	168	266	Gly-23
Tubuliform	10,146	10,146	3,381	179	346	Ala-25

#### Pseudoflagelliform spidroin (pflag)

The capture spiral of an orb web is a composite of 2 types of silk [111]. For cribellate spiders such as *U. diversus*, the core fiber is the pseudoflagelliform silk made up of pseudoflagelliform spidroin (Pflag). When produced, this core fiber is coated with finely brushed cribellate silk, which provides adhesive properties to the capture silk (see Cribellate spidroin). Pflag is understood to be homologous to the flagelliform spidroin found in ecribellate spiders, due to sequence homology and the fact that the silk glands and spinnerets that produce Pflag and Flag in cribellate and ecribellate spiders, respectively, are in the same place [43, 112–115].

We found a single candidate for Pflag on chromosome 7 (Fig. 4A and Table 7). Our results with respect to the structure of Pflag differ slightly from those reported by Correa-Garhwal et al. [43] While they reported a roughly 2,600-aa protein sequence consisting of 48 repeats, we determined that the internal structure consists of 91 repeats, each of which range between 39 and 70 aa in length. We also found that these repeats are composed of 2 parts: a glycine-poor spacer region followed by a glycine-rich repeat region with variations on the motif *PSSGGXGG*. We do not have full-length transcripts available in our own data to offer support for one or the other structure, although we do have multiple HiFi consensus reads that extend the majority of the sequence and into the terminal regions, suggesting that our assembled length is accurate.

#### Cribellate spidroin (CrSp)

Cribellate silk is produced by numerous silk glands with hundreds to thousands of spigots in the cribellum. These numerous fibers are combined into a single silk, which is combed into a “wooly” silk by calamistra located on the posterior legs. This wooly silk soaks into the waxy cuticle of insects by means of van der Waals interactions and hygroscopic forces and is used as the capture silk by cribellate orb-weavers [116]. No full-length sequence for CrSp has been reported to date, although partial spidroin sequences have been reported for some CrSps in *Tengella perfuga* [117] and several *Octonoba* species [99]. The whole genomic length of the *CrSp* locus on chromosome 10 is 20,195 nt (Fig. 4A and Table 7). The *U. diversus CrSp* gene was predicted to be a 2-exon gene, with a single 83-nt intron, which is consistent with what we found by manual inspection. While the entire *CrSp* locus was not spanned by single HiFi reads, we are still confident in the sequence produced, since no more than 2 reads were required to span the entire sequence. The N-terminal region of the predicted protein product consists of 874 aa, and the C-terminal region consists of 296 aa (Table 7). The long N-terminal domain is consistent with that found in *Octonoba* spp., which were also found to have coding regions more than 2 kbp [99]. We found an internal region that consists of variations on 4 repetitive motifs, with the first half of the sequence made up of motifs 1 and 2 alone and the second half of the sequence including all 4 motifs. Motifs 1 and 3 are similar, whereas motifs 2 and 4 are distinct from each other motif (Supplementary Fig. S1). Our approach to the classification of repeats differs here slightly from the approach used by Correa-Garhwal et al. [43]; however, our results are effectively similar.

#### Paracribellar spidroins

Correa-Garhwal et al. [43], in their recent paper investigating the evolutionary history of cribellar proteins in *U. diversus*, identified 2 spidroins that they propose represent a previously unidentified class of paracribellar spidroins, which they named Sp_vA and Sp_vB. We were able to identify both spidroin sequences in our assembly as well. While Correa-Garhwal et al. were able to recover the full-length sequence of both spidroins on single 10× contigs, we were only able to recover the full-length sequence of Sp_vB, as the Sp_vA sequence was interrupted by a gap in our assembly. These paracribellar spidroins were noted by Correa-Garhwal et al. as having similar amino acid compositions to tubuliform and aciniform spidroins, although having repetitive motifs dissimilar to either tubuliform or aciniform spidroins.

#### Major ampullate spidroin (MaSp)

Draglines are produced by the major ampullate gland, which produces 2 major ampullate spidroins (MaSp1 and MaSp2). This silk has extremely high tensile strength and elasticity and is commonly used for the primary load-bearing parts of the web such as the frame and radii. It is also the primary silk produced by spiders when they are navigating their environment [5]. We found 3 candidates for major ampullate spidroin (MaSp). Based on previous work that identified multiple distinct classes of MaSps, we were able to assign 1 of our candidate sequences to the MaSp-1 class and the other 2 candidates to the MaSp-2 class. All 3 MaSp candidates are on chromosome 6, although the MaSp-1 locus was located distantly from the 2 MaSp-2 loci (Fig. 4A).

The MaSp-1 candidate is the single spidroin sequence we were not able to call as a complete sequence. In our annotation, the sequence appears as a 2-exon gene, with the 5′ sequence and 3′ sequence found in different reading frames; however, a close inspection of the data suggests that this is not likely to be correct. We found instead that there is a large region to which the PacBio HiFi reads mapped poorly. There is consensus between the reads that indicates sequence found in the reference assembly that is not found in the reads. However, it is not clear from inspection exactly where the boundaries should be called for this region. This is likely an artifact of the assembly, since the reference was assembled from polymerase-based sequencing, which is susceptible to polymerase slippage.

The first MaSp-2 candidate, MaSp-2a, is a single-exon sequence. We found that there were 2 distinct regions. Interestingly, the first repetitive region, which is 958 aa in length, contains mostly *GPGPQ* motifs reminiscent of the *GPGPX* motifs found in the MaSp-4 sequence recently reported in *Caerostris darwini* but not elsewhere in the known catalog of spidroins [118]. The second repetitive region, which is 1,215 aa long, contains runs of poly-A and *GPX*, although *GPGPQ* repeats are also found less frequently in this region.

The second MaSp-2 candidate, MaSp-2b, is also a single-exon sequence. In the repetitive region, GPGPQ occurs in a few instances but is relatively rare compared to MaSp-2a. Alternating runs of polyalanine and variations on the motif *GSGPGQQGPGQQGPGGYGPG* characterize the repetitive region. Unlike the case of the first MaSp-2 candidate, MaSp-2b does not have 2 distinct repetitive regions.

#### Minor ampullate spidroin (MiSp)

Minor ampullate silk has lower strength but greater extensibility, and it is composed of spidroin made by the minor ampullate gland. While it is commonly used for the construction of the auxiliary spiral in orb-weavers, it is used for prey wrapping by cob-weavers [119]. We found 3 candidates for minor ampullate spidroin (MiSp). All 3 MiSp loci were located near one another on chromosome 1 (Fig. 4A).

The first candidate, MiSp-1, is a single-exon sequence (Table 7). There are 3 repetitive regions in MiSp-1, separated by short spacers. Previous work in *Araneus ventricosus* and the cobweb-weaving spiders *Latrodectus hesperus*, *Latrodectus tredecimguttatus*, *Latrodectus geometricus*, *Steatoda grossa*, and *Parasteatoda tepidariorum* has suggested that MiSp length and sequence are conserved [119, 120]; however, while the spacers we observed shared some sequence similarities, such as the presence of serine, threonine, and valine residues, the lengths of the spacers observed in *U. diversus* are much shorter.

The second and third candidates, MiSp-2a and MiSp-2b, shared a nearly identical amino acid composition, which was slightly different from that of MiSp-1. Both are single-exon sequences (Table 7). Their hydrophobicity profiles are also slightly different from MiSp-1.

#### Ampullate spidroin (AmSp)

Correa-Garhwal et al. [43] recently reported an ampullate spidroin (AmSp) sequence that could not be characterized as either MaSp or MiSp, due to the lack of characteristic repeats from either spidroin family—in fact, perhaps the most characteristic attribute of this sequence is its lack of conspicuous repetitive elements at all; however, it retains conserved N- and C-terminal amino acid signatures of these families. The full-length sequence for AmSp, which we were able to recover from the genomic and transcriptomic data released by Correa-Garhwal et al., coded for a 1,137-aa product. We were also able to recover a full-length sequence for AmSp in our assembly, which coded for a 1,130-aa single-exon product and shared 88% identity with that released by Correa-Garhwal et al.

#### Pyriform spidroin (PySp)

Pyriform silk serves as an adhesive compound used to adhere silk lines to one another or to substrate that holds the web [5]. We found one single-exon candidate, pyriform spidroin (PySp), on chromosome 1 (Fig. 4A), which is consistent in size with a prior PySp sequence reported from *Araneus ventricosus* [105]. We found that the internal repetitive region was preceded by a Q-rich N-linker region. Nineteen tandem repeat motifs, ranging from 188–196 aa, were found. This spidroin is monophyletic with araneid pyriform genes [43] and, along with ampullate, aciniform, and cribellate genes, was likely present in the last common ancestor of araneomorphs [121]. The adhesive properties of this silk are particularly important for web-building, since it enables point attachments of silk to other lines or to substrate. This, in turn, makes suspended webs possible, a trait observed in Entelegynae.

#### Tubuliform spidroin (TuSp)

Tubuliform silk is used to encase the egg sac and is spun from tubuliform glands. We found a single candidate for tubuliform spidroin (TuSp) on chromosome 2 (Fig. 4A and Table 7), which is a single exon. We found an internal region that was composed of 10 repeats, ranging from 262–302 aa in length. This is consistent with other reported TuSp repeats, which have been observed between 176 and 375 residues, although it seems that the typical TuSp module is repeated 15 to 20 times [106]. The N-terminal region has 3 Cys residues: Cys-21, Cys-52, and Cys-132. Other TuSp N-terminal sequences have been reported with 2 Cys residues [106], but Cys-21 is expected to be removed during signal peptide cleavage. After cleavage, the N-terminal domain contains 5 predicted alpha helices. Cys-52 and Cys-132 are found in alpha helix 1 and alpha helix 4 after cleavage, which is also where the AcSp Cys residues are found. This conservation suggests functionality.

### Whole-genome duplication

Gene duplication acts as a primary mode of evolutionary diversification by providing new genetic material that serves as a reservoir for subfunctionalization and neofunctionalization under selective pressure [122–124]. Previous studies in spiders uncovered evidence of a whole-genome duplication in Arachnopulmonata, including synteny between chromosomes, the presence of multiple copies of *Hox* genes [29,36,125,126], and the expansion of silk genes and chemosensory genes [36]. We identified similar properties in the *U. diversus* genome.

Hox gene cluster number has been observed as a sign of genome duplications in other species. For example in mammals, the 4 Hox gene clusters are the result of 2 genome duplication events [127]. We used published *Hox* gene sequences from the spider *Parasteatoda tepidariorum* as query sequences for BLAST searches against the *U. diversus* genome, identifying 2 *Hox* gene clusters on chromosome 5 and chromosome 10 (Fig. 4A), which were found to retain the expected order of *Hox* genes [128]. Each cluster was missing a single *Hox* gene; however, the specific missing gene was different for each of the clusters. Cluster A on chromosome 5 is missing the *fushi tarazu* (*ftz*) gene sequence, while cluster B on chromosome 10 is missing the *labial* gene sequence. Our findings are consistent with the discovery of 2 *Hox* gene clusters in *P. tepidariorum* [31]. In the genome of *T. antipodiana*, 2 *Hox* clusters were found [32], including 1 complete cluster on chromosome 12, which included a copy of all 10 expected *Hox* genes, and a second cluster on chromosome 8, which was found to be missing *abdominal-A*, *Hox3*, *ftz*, and *Ultrabithorax*. The presence of multiple *Hox* clusters in the *U. diversus* genome adds further support to an ancient, ancestral whole-genome duplication.

**Figure 5:**
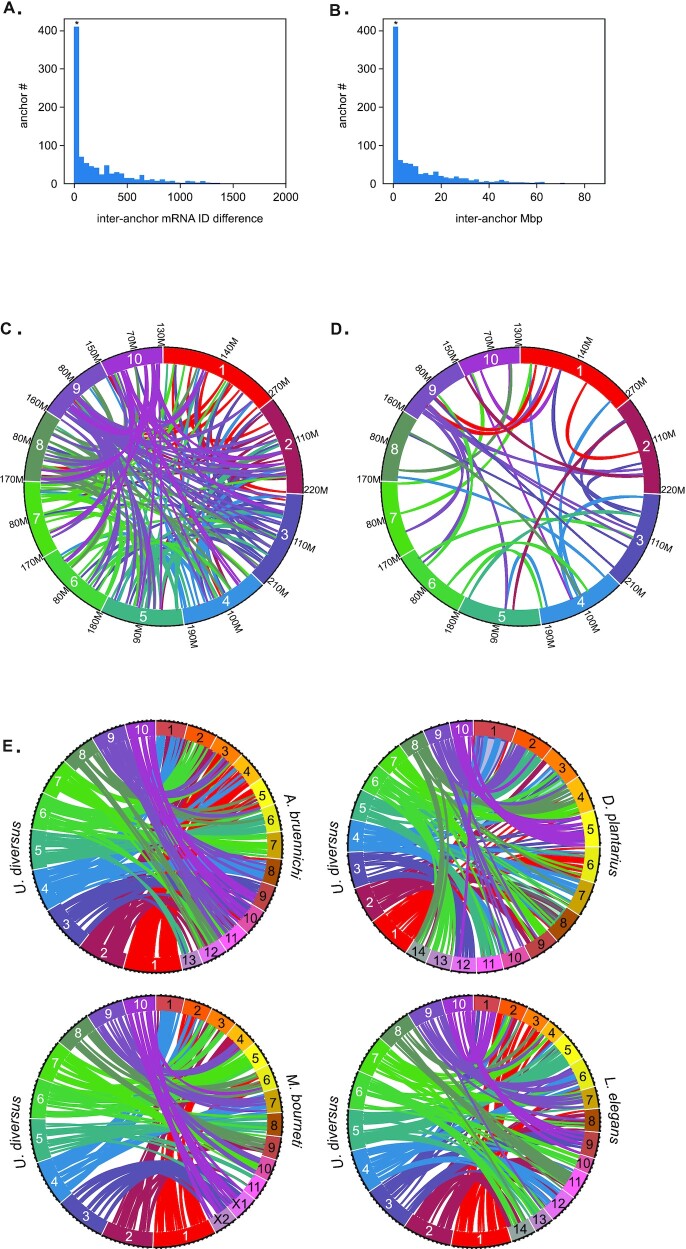
Synteny and chromosomal rearrangements. (A) Interanchor mRNA ID difference distribution of syntenic blocks identified by AnchorWave analysis. Each syntenic block is defined by ORF or inter-ORF anchors. All ORFs are numerically annotated in consecutive order from scaffold 1 through scaffold 10. Interanchor mRNA ID difference is defined as the difference in these numerical ORF IDs between consecutive ORF anchors. If the distance equals 1, it means the 2 anchors are consecutive ORFs within the block. Asterisk indicates syntenic blocks used in E. (B) Interanchor Mbp difference distribution of syntenic blocks identified with AnchorWave analysis. Interanchor difference was calculated as the base-pair distance between consecutive ORF anchors within a syntenic block. Asterisk indicates syntenic blocks used in E. (C) Ribbon plot of all AnchorWave-defined syntenic blocks shared between chromosomal scaffolds. (D) Ribbon plot of filtered AnchorWave-defined syntenic blocks shared between chromosomal scaffolds. Only blocks consisting of consecutive ORF anchors <4 mRNA IDs apart are plotted. (E) Ribbon plot of filtered AnchorWave-defined syntenic blocks shared between *U. diversus* and *A. bruennichi*, *M. bourneti*,*L. elegans*, and *D. plantarius* chromosomal scaffolds. Only blocks consisting of consecutive ORF anchors <4 mRNA IDs apart are plotted.

An additional signature of genome duplications is a high degree of synteny across chromosomes within the same genome [31, 129]. We searched for evidence of synteny between pseudochromosomes using AnchorWave [130]. The initial identification of syntenic blocks was quite ubiquitous across all 10 pseudochromosomes, but with large gaps between mRNAs and/or with very large interanchor distances. To constrain these results, we chose to include only mRNAs where the number of missing mRNAs between anchors was 4 or fewer. We made this allowance to account for the fact that we expect there to be significant loss of copies of duplicated genes [122,123, 131]. Even with this conservative threshold, it resulted in retaining 196 blocks of at least 2 mRNAs (Fig. 5). Considering the conservative nature of our analysis, this line of evidence provides further support for an ancient duplication event. However, considerable reorganization has occurred since the duplication event, an observation also made in mammalian genomes, and potentially associated with their successful adaptation to diverse environments [132,133].

We also compared the synteny of our *U. diversus* pseudochromosomes with those from *Argiope bruennichi*, an araneid orb-weaver; *Meta bourneti*, a Tetragnathid orb-weaver; and *Dolomedes plantarius*, a Pisaurid (Fig. 5). While the syntenic blocks of some of the chromosomes seem to be split between multiple chromosomes across the species, certain chromosomes, or pairs of chromosomes, have nearly exclusive synteny between species. *A. bruennichi* chromosomes 6, 7, and 8 share nearly exclusive syntenic blocks with *U. diversus* chromosomes 5, 6, and 4, respectively. This shared synteny with *U. diversus* chromosomes 5 and 6 is also observed for chromosomes 8 and 5 from *M. bourneti*, but *U. diversus*chromosome 4 is split between *M. bourneti* chromosomes 1 and 7. *U. diversus* chromosome 1 shares considerable synteny with *A. bruennichi* chromosomes 3–5, while *U. diversus* 2 is biased for shared synteny with *A. bruennichi*chromosomes 1–2 and 11–12. Even though *D. plantarius* diverged more recently from *U. diversus* (Fig. 1), there appears to be a greater degree of chromosomal rearrangement between these 2 species. However, 2 outliers are *U. diversus* 3 and 10, which share nearly exclusive synteny with *D. plantarius* 12 and 5, respectively. The large degree of synteny between *U. diversus* 3 and 10 with *M. bourneti* X1 and X2 is a strong indication that these 2 chromosomes are the sex chromosomes for *U. diversus*.

### Sex chromosomes

**Figure 6:**
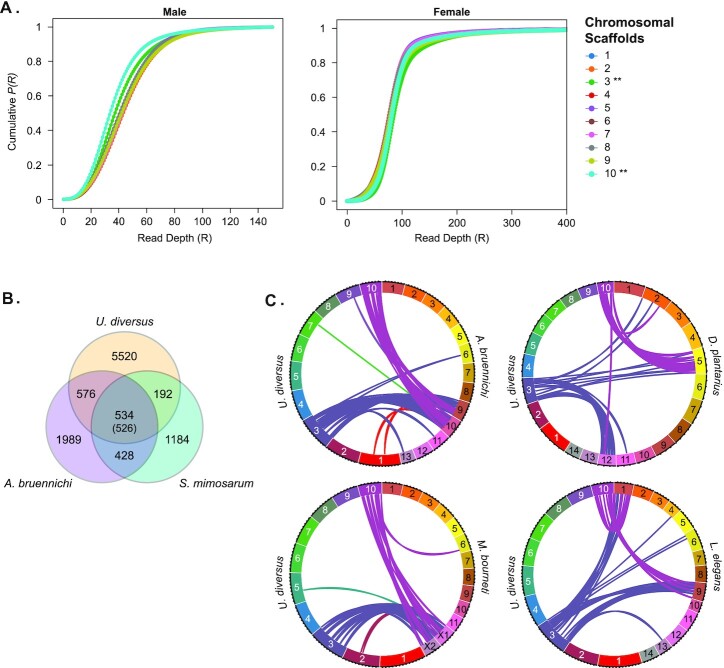
Sex chromosomes. (A) Read depth of Illumina reads from a male (left panel) and female (right panel) spider aligned to the chromosomal scaffolds. Scaffolds 3 and 10 (asterisks) exhibited lower read depth than other scaffolds in the male alignment, but not the female. (B) Venn diagram of shared sex-associated genes identified in *U. diversus*, *S. mimosarum*, and *A. bruennichi*. Number in parentheses is the number of genes that overlap with X chromosome genes from *D. plantarius*, *M. bourneti*, and *L. elegans*. (C) Ribbon plots of shared synteny between predicted X chromosomes from *U. diversus* and *A. bruennichi*,*D. plantarius*,*M. bourneti*, and *L. elegans*.

The most common and likely ancestral system of chromosomal sex determination in spiders is ♂X_1_X_2_/♀X_1_X_1_X_2_X_2_ [51]. However, spiders exhibit a diversity of sex-determining systems, with some including Y chromosomes and others possessing up to 13 X chromosomes [134]. Usually, these sex-determining systems are determined by karyotyping [51, 134] (Fig. 3). The genetic basis of sex determination and chromosomal dosage compensation are unknown for spiders. Determining the genetic identity of X chromosomes has been challenging, in part due to significant levels of shared synteny between sex chromosomes and autosomes [51] (Fig. 5), as well as a paucity of spider genomes with chromosome-level scaffolds. Sex-linked scaffolds from more fragmented genomes have been identified by quantifying the relative difference in read depth from sperm with or without the X chromosomes [135]. In this approach, the ratio of scaffold reads from sperm nuclei without X chromosomes (based on flow cytometry) to the sum of reads from all nuclei produces 2 peaks. The lower peak is from reads that were undersampled due to mapping to X chromosomes. Similarly, the X chromosomes for *A. bruennichi* (also ♂X_1_X_2_/♀X_1_X_1_X_2_X_2_) were recently identified through disparities in read coverage of X chromosomes between males and females [51]. In principle, because males have only 1 copy of each X chromosome, the average read depth for scaffolds from these chromosomes should be half that of autosomes. To determine the sex chromosome in *U. diversus*, we assembled an Illumina short-read library from a single male spider and mapped the reads onto the 10 assembled pseudochromosomes (Fig. 6).

While 8 of the 10 pseudochromsomes had a median read depth of 40 ± 2, pseudochromosomes 3 and 10 were outliers, with read depths of 36 and 33, respectively. In contrast, read depths from females were comparable for all chromosomes (Fig. 6). If all regions of these pseudochromosomes were exclusively unique to X chromosomes, the expected read depth in males would have been ∼20. However, our observations are consistent with those observed in *A. bruennichi*, where some X-associated scaffolds were observed to produce lower read depth from males as expected, and several other X-associated scaffolds had read depths comparable to autosomes [51] (Fig. 5). Why we and others observe this phenomenon is not well understood. One possibility is that reads mapping to homologous autosomal and pseudoautosomal regions on the X chromosomes should decrease the expected depth disparity between autosomes and X chromosomes and lead to a disparity of ∼75% rather than 50%, which is closer to what we observe. The higher than expected read depth could also be due to misassembly of these pseudochromosomes, but very little linkage was observed between pseudochromosomes 3 and 10 in the Hi-C data (Fig. 3). A third possibility is that spiders may have somatic chromosome copy number variation, as observed in birds and aging mammals [136, 137]. Despite these caveats, the lower median read depth in males for pseudochromosomes 3 and 10 is a strong indicator these likely represent the 2 X chromosomes for *U. diversus*.

Prior work with *Stegodyphus mimosarum* (also ♂X_1_X_2_/♀X_1_X_1_X_2_X_2_) identified sex-linked scaffolds based on lower read depth of sperm lacking X chromosomes [135]. When genes identified on these X-linked *S. mimosarum* scaffolds were mapped onto the *U. diversus* pseudochromosomes (Table 5), 62% of these genes mapped onto pseudochromosomes 3 and 10 (Fig. 6B). This large fraction of predicted X-linked genes between 2 distantly related species of spiders is a strong indicator that not only are pseudochromosomes 3 and 10 likely to be the X chromosomes but that the genetic composition of these chromosomes has remained fairly stable among spiders. Since 2 X chromosomes were recently identified in *A. bruennichi*, we compared the genetic composition (Supplementary Table S6) and synteny between the X chromosomes identified in both species (Fig. 6). In addition to shared X-linked genes (Supplementary Table S6, Fig. 6B), *A. bruennichi* scaffolds 9 and 10 appear to share considerable synteny with *U. diversus* pseudochromosomes 3 and 10 (Fig. 6C), while sharing little synteny with the predicted autosomes (Fig. 6C). This also appears to be true of the sex chromosomes X1 and X2 from *M. bourneti*, but this genome is not currently annotated. Based on this conserved X-linked synteny, we predict that chromosomes 5 and 12 from *D. plantarius* are X chromosomes, as are chromosomes 1 and 9 from *L. elegans* (Fig. 6C). When chromosomal rearrangements have occurred, they appear to have been largely confined to rearrangements between sex chromosomes. The sex chromosomes themselves share little synteny with each other (Fig. 5), which indicates they are not the result of an ancient duplication, but there appears to be selective pressure to ensure that when chromosomal rearrangements do occur, they occur between sex chromosomes. When we compared genes in the syntenic regions of the sex chromosomes from *D. plantarius* and *L. elegans* to the 534 conserved genes from *U. diversus*, *A. bruennichi*, and *S. mimosarum*, the number of X-linked genes shared between all 5 species only reduced to 526 genes, indicating there is selective pressure to retain these genomic regions on sex chromosomes.

However, some syntenic blocks are shared with autosomes. One of these syntenic blocks is the *Hox* gene cluster located on chromosome 10 for both *U. diversus* and *A. bruennichi*. The presence of a *hox* cluster on a sex chromosome was surprising since these genes play critical roles in development. Therefore, either dosage compensation is needed in males, or dosage disparity between males and females plays a role in developmental sexual dimorphism.

In some dipteran insects, the primary sex chromosome dosage sensor is *sex lethal* (*sxl*), which then triggers a cascade of sex-defining signaling events leading to sexually dimorphic expression of genes and/or splice variants. While no *sxl* homologue has been found in spider genomes (including *U. diversus*), other genes involved in sexual dimorphism, such as *doublesex* (*dsx*), are present. Thus, while sex-determining systems may be conserved, the mechanism spiders use for sensing X/autosome ratio differences to trigger these circuits remains unknown, but relevant genes are likely in the regions containing the 526 genes conserved among the 5 species. Of the 526 shared X-linked genes in these 5 species, 14 are predicted to be DNA/RNA binding and may play a role in sex determination. However, non-protein-coding RNAs (such as Xist in mammals) can also act as effectors for dosage compensation; therefore, the sex-trigger mechanism in spiders may not be the 526 conserved protein-coding genes but unidentified small RNA genes in these regions. Regardless, the X-linked genes shared between these 5 species (Supplementary Table S6) will be a valuable resource for comparative analysis to identify conserved genes that serve as sex-specifying triggers for spiders. Uncovering how spiders perform sex-linked dosage compensation can not only illuminate how arthropods evolved different sex-determining systems but also how dosage compensation has evolved independently in numerous animals.

## Discussion

Here, we present a high-quality chromosome-level genome and complementary transcriptome assembly of the hackled orb-weaver *U. diversus*. The 2.15-Gbp draft genome assembly comprises 1,586 scaffolds, including 10 pseudochromosomes that contain 1.9 Gbp (88%) of the total assembly, comparable to the estimated genome size (1.98 Gbp) predicted by GenomeScope2 and contains the vast majority of highly conserved orthologs (94.1% complete, with 88.6% complete and in single copy), as estimated by BUSCO. We predicted a total of 44,408 protein-coding gene models with a BUSCO completeness of 86.7%. Despite the aforementioned technical hurdles, the contiguity and completeness of this assembly, along with the recovery of a complete catalog of full-length spidroin gene sequences, demonstrates the utility of using multiple complementary sequencing technologies for large, repetitive, and highly heterozygous genomes.

The repetitive nature and length of spidroin genes have posed a technical challenge for identifying and reporting full-length sequences. However, it is exactly these qualities that lend spidroins their unique mechanical properties [138–140], underscoring the need for accurate assemblies. Recent studies leveraging single-molecule, long-read sequencing technology have predicted longer spidroin sequences than those using PCR approaches [28]. Here, we used ONT and PacBio HiFi reads to achieve a complete catalog of full-length spidroin sequences for *U. diversus*. The ability to recover full-length sequences for this family of genes is an indication of the high quality of the assembly. In addition, complete sequences enable us to more fully analyze the evolutionary relationships between spidroins.

Web-weaving spiders, orb-weavers in particular, have evolved numerous spidroins with unique functionality for a diversity of uses in their webs. Both ecribellate and cribellate orb-weavers produce pyriform silk that is used to create attachment disks for bonding silk fibers together, an important property of orb webs. Ecribellate spiders use flagelliform in-capture spiral construction. Cribellate spiders use a homologous protein, pseudoflagelliform, to construct their capture spiral as well. These proteins are likely the result of a duplication since flagelliform is absent in Uloborids but present in at least 2 RTA families [43]. Since these spidroins and the spinnerets that produce them [43] and their roles in orb-weaving are conserved between cribellate and ecribellate spiders, as are other nonaggregate/cribellate spidroins, the structural components of orb-weaving likely have a single evolutionary origin. The behaviors and phases of web-building are remarkably similar [141], though whether the behaviors themselves are of a single origin or convergently evolved to use the same structural components will require a deeper genetic understanding of how these behaviors are encoded in the brain. However, if orb-weaving behaviors evolved independently to use the same spidroins for the same structural elements of the orb web, it begs the question: How were these spidroins used in web construction prior to the convergence of the orb web?

All current models of chromosomal dosage compensation are based on single-sex chromosome animals, but multiple sex chromosome systems exist in both vertebrates and invertebrates [142, 143]. Spiders exhibit considerable morphologic and behavioral sexual dimorphism that is based on a multiple sex chromosome system. Understanding the genetic underpinnings of spider sexual development will contribute to a fuller understanding of how chromosomal sex determination can evolve independently in different species. Here we provide evidence for the identities of sex chromosomes in *U. diversus* and leverage this information to identify 14 candidate DNA-binding genes that are shared between 3 divergent species of spiders.

Our genome will facilitate comparative studies and meets a specific need in the field for a greater representation of genomes from the UDOH + RTA clade that represent nearly half of all known spider species (Fig. 1) [44]. We expect that the highly contiguous draft genome and transcriptome datasets we produced for *U. diversus* will serve as a valuable resource for continuing research into the evolution, development, and physiology of spiders, as well as a vital tool to study the genetic basis of orb-weaving behavior. While a handful of spider genomes have been published, all orb-weaving genomes have been from ecribellate araneid spiders, with no representative genomes from the cribellate families Uloboridae, Deinopidae, Oecobiidae, or Hersiliidae. Improved knowledge of genomes from these families, combined with behavioral and cellular analyses of orb-weaving behavior, will offer a crucial foundation for understanding how and when orb-weaving evolved.

## Materials and Methods

### Sample collection and husbandry

We collected spiders of the species *U. diversus* from the ancestral lands of the Ramaytush, in Half Moon Bay, California, USA. We collected colony founders from a single greenhouse during several trips between 2016 and 2019 and transported them to custom-fabricated habitats in an on-campus greenhouse at Johns Hopkins University. We later transferred experimental animals from the on-site greenhouse to custom-fabricated habitats in the laboratory until required for experiments. We fed all animals alternately *Drosophila melanogaster* or *Drosophila virilis* once per week.

### Karyotyping

We soaked embryos soaked in Grace's insect medium (ThermoFisher Scientific, Waltham, MA, USA) containing 0.1% colchicine for 2 hours. We then added an equal volume of hypotonic solution. After 15 minutes, we transferred the embryos to a 3:1 ethanol/acetic acid solution for 1 hour. After fixing, we transferred embryos to gelatin-coated microscope slides and dissociated them in a drop of 45% acetic acid. We used siliconized coverslips to squash the dissociated tissue and briefly froze them in liquid nitrogen. After removing the slides from LN2, we immediately removed the coverslips with a razor blade and transferred the slides as quickly as possible to 95% ethanol. We then performed a step-down series from 95% ethanol to 70%, 35%, and finally to Grace's insect medium to return the tissue to an aqueous solution. We then transferred the slides to a 1-µg/mL DAPI solution. After a 10-minute incubation, we transferred the slides to deionized water to rinse and mounted coverslips with a drop of Vectamount (Vector Laboratories, Burlingame, CA, USA).

### RNA extraction, library preparation, and sequencing

We extracted total RNA from multiple samples: a whole adult female, a whole adult male, adult female prosoma and opisthosoma, adult male prosoma and opisthosoma, pooled legs from both the adult female and adult male dissections, a fourth instar female, and approximately 30 pooled second instars. We used the Qiagen RNeasy Mini Kit (Qiagen, Hilden, Germany) to extract total RNA, following the manufacturer's protocol. We estimated the quality and quantity of total RNA using a NanoDrop One Microvolume UV-Vis Spectrophotometer (ThermoFisher Scientific, Waltham, MA, USA). Before library preparation, we also measured the quality, quantity, and fragment length of our total RNA using a TapeStation 4200 System with RNA ScreenTape and reagents (Agilent, Santa Clara, CA, USA). We prepared barcoded, directional, paired-end RNA-seq libraries with the NEBNext Ultra II Directional RNA Library Prep Kit for Illumina (San Diego, CA, USA) using the NEBNext Poly(A) mRNA Magnetic Isolation Module. We submitted the resulting libraries to the Johns Hopkins Genomics Core Resources Facility to be sequenced on an Illumina HiSeq 2500 Sequencing System with 150-bp paired-end chemistry.

### Genomic DNA extraction, library preparation, and sequencing

Prior to extraction of DNA, we withdrew food for 3 days to minimize the potential contribution of contaminating DNA from dietary sources. We extracted high molecular weight (HMW) DNA using the QIAgen MagAttract HMW DNA kit. Prior to HMW purification, we followed the manufacturer's protocol for disruption/lysis of tissue. We avoided fast pipetting and prolonged vortexing to minimize shearing of DNA. We flash-froze adult spiders in liquid N_2_ and crushed them with a pellet pestle (Fisher, 12–141–364) in a Protein LoBind tube (Eppendorf, 022431081) containing 220 μL Buffer ATL. We then added 20 μL Proteinase K and briefly vortexed the sample. We next incubated the sample overnight at 56°C with 900 rpm shaking on a ThermoMixer C (Eppendorf, 5382000023). After the overnight incubation, we then briefly centrifuged the sample to spin down condensate on the tube. We next transferred 200 μL lysate to a fresh 2-mL sample tube and followed the manufacturer's protocol for manual purification of HMW DNA from fresh or frozen tissue. We estimated DNA quality using a NanoDrop One Microvolume UV-Vis Spectrophotometer and quantified DNA using a Qubit 4 Fluorometer (ThermoFisher) with a Quant-iT dsDNA HS Assay Kit. We also measured DNA quality, quantity, and fragment length distributions using the Agilent TapeStation 4200 System with Genomic DNA ScreenTape and reagents before proceeding to library preparation. A typical preparation from a 20-mg spider yielded 8.5 μg DNA with a major integrated area percentage peaks accounting for an average of 86.7% of the total mass centered around an average fragment length of 20 kbp.

#### Illumina sequencing

For Illumina sequencing, we extracted genomic DNA from a single, whole, unmated penultimate stage female to minimize the potential contribution of extraneous haplotypes from stored sperm after mating events. We submitted the HMW genomic DNA to the Johns Hopkins Genetic Resources Core Facility, where they prepared a PCR-free library of approximately 400-bp DNA insert size using the Illumina TruSeq PCR-Free High Throughput Library Prep Kit, according to the manufacturer's protocol. They then sequenced the prepared library on an Illumina NovaSeq 6000 Sequencing System (RRID:SCR_016387) with 150-bp paired-end chemistry.

#### ONT sequencing

For ONT sequencing, we extracted HMW genomic DNA from 3 adult females. We prepared sequencing libraries using the Ligation Sequencing Kit (SQK-LSK109) (Oxford Nanopore Technologies, Oxford, UK), according to the manufacturer's protocols. Third-party reagents we used during library preparation included New England Biolabs (Ipswich, MA, USA) NEBNext End Repair/dA-Tailing Module (E7546), NEBNext FFPE DNA Repair Mix (M6630), and NEB Quick Ligation Module (E6056). We then sequenced the libraries, using ONT R.9.4.1 flowcells (FLO-PRO002), on an ONT PromethION (RRID:SCR_017987) sequencing platform. We then used ONT's Albacore basecalling software v.2.0.1 (RRID:SCR_015897) to basecall the raw fast5 data.

#### PacBio HiFi sequencing

For PacBio sequencing, HMW DNA were extracted from a single adult female spider provided to Circulomics (Baltimore, MD, USA). They extracted DNA using a modified protocol with the Nanobind Tissue Kit (Circulomics, #NB-900–701–01). Briefly, they froze and crushed a single, adult female spider with a pellet pestle (Fisher, #12–141–364) in a Protein LoBind tube (Eppendorf, #022431081) containing 200 μL Buffer CT. The crushed spider was centrifuged at 16,000 × *g* at 4°C for 2 minutes. The supernatant was discarded, and the pellet was resuspended in 500 μL Buffer CT, and the mixture was transferred to a 2.0-mL Protein LoBind tube (Eppendorf, #022431102). The suspension was spun again at 16,000 × *g* at 4°C for 2 minutes and the supernatant discarded. The spider tissue pellet was combined with 20 μL Proteinase K and 150 μL Buffer PL1 and resuspended by pipetting with a P200 wide-bore pipette tip. The tissue was incubated on a ThermoMixer at 55°C with 900 rpm mixing for 1 hour. After lysis, 20 μL RNaseA was added, and the lysate was mixed by pipetting with a P200 wide-bore pipette tip. The lysate was incubated at room temperature for 3 minutes. After RNaseA incubation, 25 μL Buffer SB was added, and the lysate was vortexed 5 × 1-second pulses and then centrifuged at 16,000 × *g* at 4°C for 5 minutes. The supernatant (∼200 μL) was transferred to a 70-μM filter (Fisher, #NC1444112) set in a new 1.5-mL Protein LoBind tube (Eppendorf, #022431081). The tube with the 70-μM filter was spun on a mini-centrifuge (Ohaus, #FC5306) for 1 second and then the filter was discarded. Then, 50 μL Buffer BL3 was added to the cleared lysate and the tube was inversion mixed 10×. The tube was then incubated on a ThermoMixer at 55°C with 900 rpm mixing for 5 minutes. After incubation, the tube was allowed to come to RT, which took about 2 minutes. The tube was spun for 1 second on a mini-centrifuge to spin down condensate from the lid. One 5-mm Nanobind disk was added to the tube followed by 250 μL isopropanol and then the tube was inversion mixed 5×. The tube was then rocked on a platform rocker (ThermoScientific, #M48725Q) at RT and maximum speed for 30 minutes. The DNA-bound Nanobind disk was washed according to handbook directions with one 500-μL CW1 wash and one 500-μL CW2 wash. The tube with the disk was tap spun for 2 × 1 second to dry the disk. The DNA was eluted with 50 μL Buffer EB and incubated at RT overnight. The next day, the eluate was pipette mixed with a standard bore pipette tip 5× and then quantitated with Nanodrop and Qubit dsDNA BR assay and then sized by pulsed-field gel electrophoresis.

We then submitted the DNA sample to the University of Maryland School of Medicine Genomics Core Facility for PacBio HiFi sequencing. There, they size-selected the DNA using a Safe Science BluePippin with a 9-kb high-pass cutoff. They prepared the sequencing library using the Express v2 kit, according to the standard protocol for preparing HiFi sequencing libraries. They then sequenced the library on a PacBio Sequel II (RRID:SCR_017990) 8 M SMRT Cell using a 30-hour HiFi run mode and processed using SMRT Link v.9.0 software.

### Dovetail Chicago and Dovetail Hi-C sequencing

To further improve the *U. diversus* genome assembly, we used proximity ligation-based sequencing techniques to scaffold intermediate versions of our assembly. We provided 19 spider specimens to Dovetail Genomics (Scotts Valley, CA, USA) for Chicago and Hi-C library preparation as previously described [57]. They prepared a Chicago library using 15 pooled adult females and a Hi-C library using 4 pooled adult females. They sequenced both the prepared Chicago and Dovetail Hi-C libraries on an Illumina HiSeq X sequencing platform (Illumina HiSeq X Ten, RRID:SCR_016385) on 1 flowcell.

### DNA-seq and RNA-seq Quality Assurance and Control

For Illumina, we examined read quality using FastQC [144] v.0.11.9 (RRID:SCR_014583). For DNA-seq data, we determined that, due to the high quality of reads and the absence of adapter sequences, no further processing would be required and proceeded to assembly with raw read data. For RNA-seq data, we used TrimGalore [145] v.0.4.2 (RRID:SCR_011847) to apply quality filtering and remove adapter sequences from the FASTQ files. We performed additional filtering for quality with Trimmomatic [146] v.0.33 (RRID:SCR_011848). For ONT, reads shorter than 3 kbp were discarded. The length-filtered ONT long reads were used in downstream assembly.

### Genome size, heterozygosity, and unique sequence estimation

Prior to assembly, we used Jellyfish [49] v.2.2.4 (RRID:SCR_005491) to count the frequency of canonical 21-mers in our Illumina sequencing data. We used the resulting sorted *k*-mer frequencies versus counts histogram as input to GenomeScope [50,147] v.2.0 (RRID:SCR_017014) to estimate genome size, heterozygosity, and repetitiveness.

### Recovery of mitogenome

We used Novoplasty [72] v.4.2 (RRID:SCR_017335) to generate a complete circularized mitochondrial sequence using raw Illumina read data. The mitochondrial sequences of several spider species were used to provide seed sequences (Supplementary Table S3). The resulting mitogenome sequences assembled by Novoplasty were compared for consensus. The consensus mitogenome was uploaded to the MITOS 2 web server [87] for annotation. The CGView web server [148] (RRID:SCR_011779) was used to visualize the annotated mitogenome.

### Nuclear genome assembly

#### De novo nuclear genome assembly with MaSuRCA

Illumina reads were assembled into contigs, and the resulting contigs were scaffolded with ONT long reads using the MaSuRCA assembly pipeline [52, 53] v.3.4.2 (RRID:SCR_010691). We used default settings, including the default CABOG contigging module, in lieu of the Flye assembler. The resulting genome assembly is referred to as *U. diversus* v.1.0.

To improve the assembly, we used *Rascaf* [54] v.2016–11–29 (RRID:SCR_022014) to scaffold with Illumina RNA-seq read data. The resulting genome assembly is referred to as *U. diversus* v.1.1. To reduce redundancy in the assembly due to the presence of alternative haplotigs, we used Pseudohaploid with default settings. The resulting genome assembly is referred to as *U. diversus* v.1.2

#### De novo nuclear genome assembly with PB-IPA

We used PacBio's IPA HiFi Genome Assembler v.1.3.2 (IPA HiFi Genome Assembler, RRID:SCR_021966) with default settings, specifying a genome size of 1.9 Gbp, to assemble the HiFi reads. The resulting genome assembly is referred to as *U. diversus* v.2.0.

#### Merging MaSuRCA and PB-IPA assemblies with SAMBA

We used the SAMBA tool distributed with MaSuRCA to merge the MaSuRCA assembly, *U. diversus* v.1.2, and the PB-IPA assembly, *U. diversus* v.2.0. The resulting genome assembly is referred to as *U. diversus* v.3.0.

### Scaffolding assemblies with *HiRise*

The initial *U. diversus* v.1.2 draft assembly obtained using a combination of MaSuRCA, Rascaf, and Pseudohaploid was provided to Dovetail Genomics in FASTA format. The resulting genome assembly is referred to as *U. diversus* v.1.3.

The merged MaSuRCA and PB-IPA assembly, *U. diversus* v.3.0, was provided to Dovetail Genomics in FASTA format. The resulting genome assembly is referred to as *U. diversus* v.3.1.

### Genome assembly metrics and assessments

For each assembly, completeness was estimated with BUSCO [149–151] v.5.2.1 (RRID:SCR_015008) using the arachnida_odb10 database [152]. Contiguity of each assembly was evaluated for comparison using Quast [153] v.5.0.2 (RRID:SCR_001228).

### Genome-guided transcriptome assembly

Cleaned and trimmed Illumina RNA-seq reads were aligned to the genome using *HISAT2* [154] v.2.2.1 (RRID:SCR_015530). We then used the Trinity assembler v.2.12.0 (RRID:SCR_013048) to produce a genome-guided transcriptome assembly (–CPU 60 –max_memory 200 G –genome_guided_max_intron 20000 –SS_lib_type RF –include_supertranscripts –verbose). We used TransDecoder [60] v.5.5.0 (RRID:SCR_017647) with default settings, including homology searches using both BlastP [61, 62] (RRID:SCR_001010) against a SwissProt UniProt database [155] (UniProtKB/Swiss-Prot, RRID:SCR_021164), as well as the Pfam database [63] v.32 (RRID:SCR_004726), as ORF retention criteria.

### Repeat annotations

To characterize the repeat elements in the *U. diversus* genome, we generated a custom *de novo* repeat library using RepeatModeler [58] v.2.0.2 (RRID:SCR_015027) with default parameters. We used RepeatMasker [156] v.4.1.2 (RRID:SCR_012954) to screen and mask repeat and low-complexity regions of the genome with the Dfam consensus [157] v.3.4 (RRID:SCR_021168) and RepBase RepeatMasker Edition [59] v.2018–10–26 (RRID:SCR_021169) repeat libraries.

### Annotation of protein coding genes

We performed gene annotation using the BRAKER2 pipeline [64, 65, 158–168] v.2.1.6 (RRID:SCR_018964) with RNA-seq evidence and protein homology evidence based on a custom library of spider sequences obtained from NCBI. BRAKER2 uses RNA-seq data to produce intron hints for training the *ab initio* gene prediction program AUGUSTUS (RRID:SCR_008417) [160, 161] on a species-specific model. This species-specific model is then used in conjunction with RNA-seq data to predict protein-coding genes. The bam file previously generated in transcriptome assembly and analysis was passed to BRAKER2, which was run with default settings.

### Annotation of noncoding RNAs

We used tRNAscan-SE [68, 69] v.2.0.7 (RRID:SCR_010835) with default settings to predict tRNAs. We then used Barrnap [70] v.0.9 (RRID:SCR_015995) with default settings to predict rRNAs.

### Functional annotation

We started the annotation of predicted genes used the BLAST+ BLASTP algorithm. First, we obtained the longest coding sequence for each gene predicted by BRAKER2. We then used the EMBOSS [169] v.6.6.0.0 Transeq tool (RRID:SCR_015647) to translate and trim the coding sequences. Once translated and trimmed, we used the BLAST+ v.2.10.1+ Blastp tool to search against the UniProt SwissProt database with an e-value cutoff of 1e-10. We used InterProScan [66, 67] (RRID:SCR_005829) to predict motifs, domains, and gene ontology (GO) [170,171] terms (RRID:SCR_002811), as well as MetaCyc [172, 173] (RRID:SCR_007778) and Reactome [174, 175] (RRID:SCR_003485) pathways, using the following analyses: CDD [176] v.3.18 (RRID:SCR_002077), Coils v.2.2.1 (RRID:SCR_008440), Gene3D [177] v.4.3.0 (RRID:SCR_007672), Hamap [178] v.2020–05 (RRID:SCR_007701), MobiDBLite [179] v.2.0 (RRID:SCR_014542), PANTHER [180] v.15.0 (RRID:SCR_004869), Pfam [63] v.34.0, the PIR PIRSF [181] v.3.10 and PIRSR [182] v.2021–02 (RRID:SCR_003352) databases, PRINTS [183] v.42.0 (RRID:SCR_003412), the ProSite (RRID:SCR_003457) ProSitePatterns [184, 185] v.2021–01 and ProSiteProfiles [184, 185] v.2021–01 databases, SFLD [186] v.4 (RRID:SCR_001375), SMART [187, 188] v.7.1 (RRID:SCR_005026), SUPERFAMILY [189, 190] v.1.75 (RRID:SCR_007952), and TIGRFAMS [191–194] v.15.0 (RRID:SCR_005493).

### Spidroins

#### Identification of spidroin candidate sequences

We identified *U. diversus* by conducting BLAST [61, 62] searches using the list of spidroin sequences included in Supplementary Table S5 as queries against the assembled genome, transcriptome, and gene models predicted by BRAKER2. We looked for matches to both N- and C-terminal sequences from members of each type of spidroin, as well as to available repetitive motifs. After cross-referencing genomic coordinates with gene models and transcripts, we used JBrowse [195] (RRID:SCR_001004) to visualize mapping of Illumina RNA-seq data and PacBio HiFi reads to the assembled genome. RNA-seq reads were mapped to the genome with HISAT2 [154], while minimap2 [196, 197] (RRID:SCR_018550) was used to map PacBio HiFi reads. Samtools [163] was used to convert the resulting SAM files to BAM files, as well as to sort and index the BAM files. For each spidroin candidate, the entire sequence from start codon to stop codon, ignoring any predicted splicing, with an additional 5 kb of sequence on both the 5′ and 3′ end, was translated in all 6 frames using the ExPASy Translate Tool via the ExPASy web server [198] (RRID:SCR_012880) and inspected for ORFs as well as the presence of repetitive motifs characteristic of spidroins. Predicted splice sites were compared with RNA-seq data. Unsupported splice sites, either by lack of evidence in the mapping of RNA-seq reads or by the obvious presence of spidroin repeat motifs within the predicted intronic region, were removed from the annotations. Spidroins sequences were called based upon the preponderance of available evidence, which in some cases conflicted with the structure predicted by BRAKER2.

#### Spidroin sequence analysis

We used the ExPASy web server tool ProtScale to find the amino acid composition of each sequence, as well as to estimate the hydrophobicity using the Kyte–Doolittle method [198,199]. We used the PSIPRED v.4.0 tool in the UCL Bioinformatics Group's PSIPRED Protein Analysis Workbench [200] (RRID:SCR_010246) to predict the secondary structure of each sequence. The sequences were often too long and necessitated judicious segmentation into reasonable sequences that were short enough for analysis. In such cases, we selected natural breaks in the sequence structure, such as separating the N-terminal region from the repetitive regions. We used SignalP [201] v.6.0 (RRID:SCR_015644) to predict the presence of signal peptides and signal peptidase cleavage sites in the N-terminal regions.

## Data Availability

The raw sequencing data and assembled genome presented in this study have been submitted to the NCBI BioProject database under accession number PRNA846873. All supporting data are available in the *GigaScience* GigaDB database [202].

## Additional Files

**Supplementary Fig. S1**. Sex Chromosomes.

**Supplementary Table S1**. Comparison of Genome Statistics for Published Genomes.

**Supplementary Table S2**. Summary of Spider Genome Repeat Content.

**Supplementary Table S3**. Library of Annotated Genes from Spider Genomes.

**Supplementary Table S4**. Mitogenome Sequences Used for NOVOplasty Seeds.

**Supplementary Table S5**. Spidroin Protein Sequences Used in BLAST Searches.

**Supplementary Table S6**. Summary of Common Sex-Linked Annotations.

**Spidroin_sequences.txt**.Text file containing *U. diversus* spidroin protein sequences.

## Abbreviations

AcSp: aciniform spidroin; AmSp: ampullate spidroin; BLAST: Basic Local Alignment Search Tool; bp: base pair; BUSCO: Benchmarking Universal Single-Copy Orthologs; Gb: gigabase; Gbp: gigabase pair; HMW: high molecular weight; IPA: Improved Phased Assembly; kb: kilobase; MaSp: major spidroin; Mbp: megabase pair; MiSp: minor spidroin; ORF: open reading frame; PySp: pyriform spidroin; RNA-seq: RNA sequencing; RTA: retrolateral tibial apophysis; tRNA; transfer RNA; TuSp: tubuliform spidroin; UDOH: Uloboridae, Deinopidae, Oecobiidae, Hersiliidae.

## Competing Interests

The authors declare they have no competing interests.

## Funding

J.M. acknowledges funding from the NSF Graduate Research Fellowship Program (DGE-1746891). A.G. acknowledges funding from NIH (R35GM124883). A.V.Z. acknowledges funding from the USDA National Institute of Food and Agriculture (2018–67015–28199), NSF (IOS-1744309), and NIH (R01-HG006677 and R35-GM130151).

## Authors' Contributions

J.M., A.Z., and A.G. designed the research study. J.M. performed DNA purification and sample preparation for Illumina and Oxford Nanopore sequencing. J.M. performed all computational analyses, except for HiRise scaffolding (performed by Dovetail), MaSuRCA, and SAMBA. A.Z. performed MaSuRCA assembly and merging with SAMBA. J.M. and A.G. analyzed the data and wrote the paper.

## Supplementary Material

giad002_Spidroin_sequences

giad002_Figure_Supplemental_1

giad002_Table_S1

giad002_Table_S2

giad002_Table_S3

giad002_Table_S4

giad002_Table_S5

giad002_Table_S6

giad002_GIGA-D-22-00169_Original_Submission

giad002_GIGA-D-22-00169_Revision_1

giad002_Response_to_Reviewer_Comments_Original_Submission

giad002_Reviewer_1_Report_Original_Submission Jonathan Coddington -- 8/10/2022

giad002_Reviewer_1_Report_Revision_1 Hui Xiang, PH.D -- 11/24/2022

giad002_Reviewer_2_Report_Original_Submission Hui Xiang, PH.D -- 8/13/2022

giad002_Reviewer_3_Report_Original_Submission Zhisheng Zhang, Ph.D -- 8/20/2022
