# Supplementary figures and images for: Chromosome-level genome and the identification of sex chromosomes in Uloborus diversus
Source: Gigascience. 2023 Feb 10;12:giad002. doi: 10.1093/gigascience/giad002 (PMC9912274; doi:10.1093/gigascience/giad002)

Supplemental Figure 1

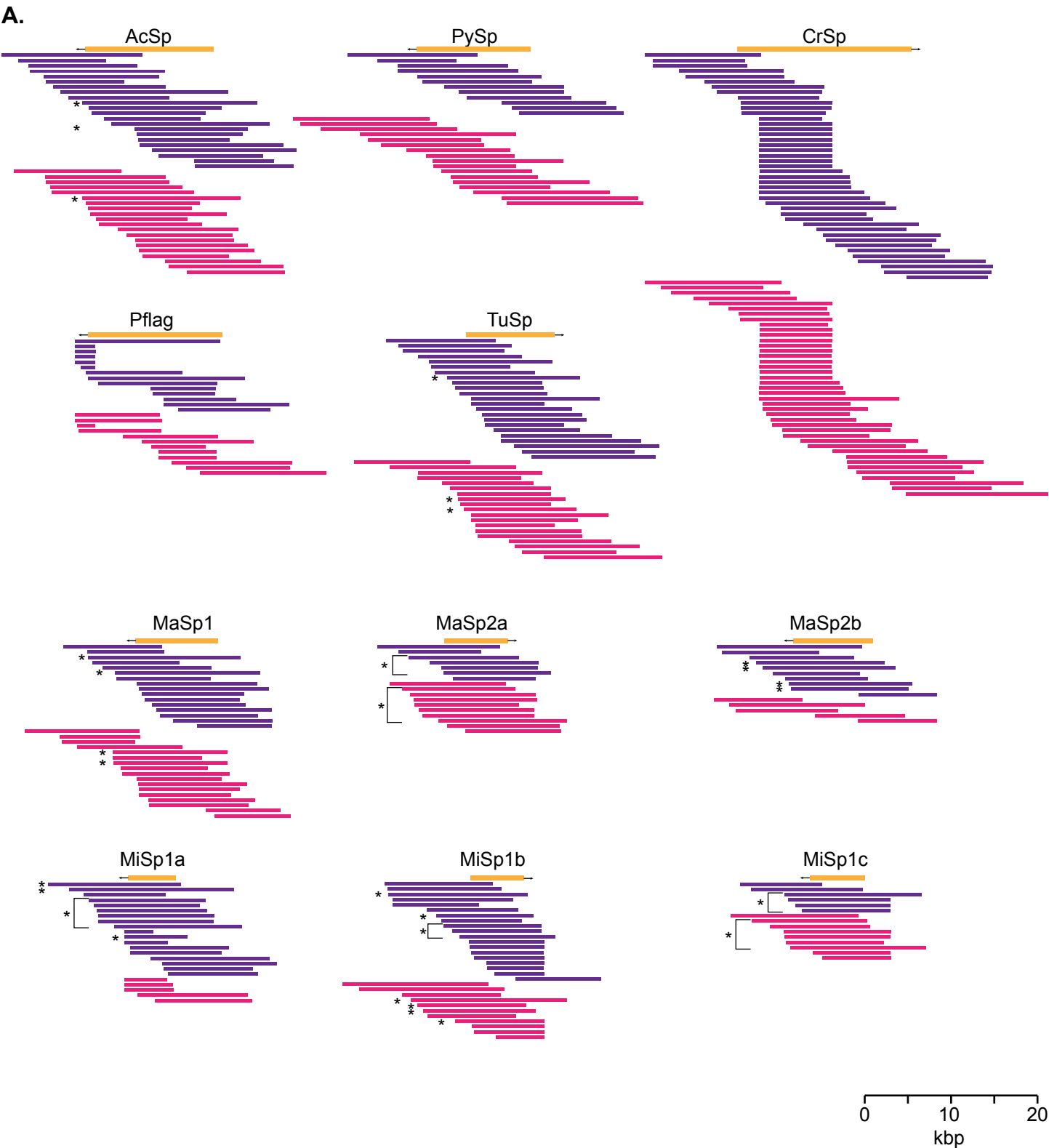

Supplement: giad002_Figure_Supplemental_1 [file giad002_figure_supplemental_1.pdf]
