# Supplementary material for: Chromosome-level genome and the identification of sex chromosomes in Uloborus diversus
Source: Gigascience. 2023 Feb 10;12:giad002. doi: 10.1093/gigascience/giad002 (PMC9912274; doi:10.1093/gigascience/giad002)
Supplement: giad002_GIGA-D-22-00169_Original_Submission [file giad002_giga-d-22-00169_original_submission.pdf]

GigaScience

Chromosome-level genome and the identification of sex chromosomes in *Uloborus diversus*

--Manuscript Draft--

**Manuscript Number:**	GIGA-D-22-00169
**Full Title:**	Chromosome-level genome and the identification of sex chromosomes in *Uloborus diversus*
**Article Type:**	Research
**Funding Information:**	Division of Graduate Education (DGE-1746891) — Mr. Jeremiah Miller<br>National Institute of General Medical Sciences (R35GM124883) — Dr. Andrew Gordus<br>Agricultural Research Service (2018-67015-28199) — Dr. Aleksey V Zimin<br>Division of Integrative Organismal Systems (IOS-1744309) — Dr. Aleksey V Zimin<br>National Human Genome Research Institute (R01-HG006677) — Dr. Aleksey V Zimin<br>National Institute of General Medical Sciences (R35GM130151) — Dr. Aleksey V Zimin
**Abstract:**	The orb-web is a remarkable example of animal architecture that is observed in families of spiders that diverged over 200 million years ago. While several genomes exist for Araneid orb-weavers, none exist for other orb-weaving families, hampering efforts to investigate the genetic basis of this complex behavior. Here we present a chromosome-level genome assembly for the cribellate orb-weaving spider *Uloborus diversus*. The assembly reinforces evidence of an ancient arachnid genome duplication and identifies complete open reading frames for every class of spidroin gene, which encode the proteins that are the key structural components of spider silks. We identified the two X chromosomes for *U. diversus* and identify candidate sex-determining genes. This chromosome-level assembly will be a valuable resource for evolutionary research into the origins of orb-weaving, spidroin evolution, chromosomal rearrangement, and chromosomal sex-determination in spiders.
**Corresponding Author:**	Andrew Gordus, Ph.D.<br>Johns Hopkins University - Homewood Campus: Johns Hopkins University<br>Baltimore, MD UNITED STATES
**Corresponding Author Secondary Information:**	
**Corresponding Author's Institution:**	Johns Hopkins University - Homewood Campus: Johns Hopkins University
**Corresponding Author's Secondary Institution:**	
**First Author:**	Jeremiah Miller
**First Author Secondary Information:**	
**Order of Authors:**	Jeremiah Miller<br>Aleksey V Zimin, Ph.D.<br>Andrew Gordus, Ph.D.
**Order of Authors Secondary Information:**	
**Additional Information:**	
**Question**	**Response**

Powered by Editorial Manager® and ProduXion Manager® from Aries Systems Corporation

Are you submitting this manuscript to a special series or article collection?	No
**Experimental design and statistics**<br><br>Full details of the experimental design and statistical methods used should be given in the Methods section, as detailed in our Minimum Standards Reporting Checklist. Information essential to interpreting the data presented should be made available in the figure legends.<br><br>Have you included all the information requested in your manuscript?	Yes
**Resources**<br><br>A description of all resources used, including antibodies, cell lines, animals and software tools, with enough information to allow them to be uniquely identified, should be included in the Methods section. Authors are strongly encouraged to cite Research Resource Identifiers (RRIDs) for antibodies, model organisms and tools, where possible.<br><br>Have you included the information requested as detailed in our Minimum Standards Reporting Checklist?	Yes
**Availability of data and materials**<br><br>All datasets and code on which the conclusions of the paper rely must be either included in your submission or deposited in publicly available repositories (where available and ethically appropriate), referencing such data using a unique identifier in the references and in the "Availability of Data and Materials" section of your manuscript.<br><br>Have you have met the above requirement as detailed in our Minimum	Yes

Standards Reporting Checklist?	

Powered by Editorial Manager® and ProduXion Manager® from Aries Systems Corporation

Manuscript
Click here to access/download;Manuscript;Miller_Draft_GIGA_v.6.2.JM.docx

Chromosome-level genome and the identification of sex chromosomes in Uloborus diversus.

Authors: Jeremiah Miller[1], Aleksey V Zimin[2,3], Andrew Gordus[1,4]

[1] Department of Biology, Johns Hopkins University, Baltimore, MD
[2] Department of Biomedical Engineering, Johns Hopkins University, Baltimore, MD
[3] Center for Computational Biology, Johns Hopkins University, Baltimore, MD
[4] Solomon H. Snyder Department of Neuroscience, Johns Hopkins University, Baltimore, MD

Corresponding Author:
Andrew Gordus
agordus@jhu.edu
410-516-6509
Johns Hopkins University
3400 N. Charles St.
Mudd Hall
Baltimore, MD 21218

Abstract

The orb-web is a remarkable example of animal architecture that is observed in families of spiders that diverged over 200 million years ago. While several genomes exist for Araneid orb-weavers, none exist for other orb-weaving families, hampering efforts to investigate the genetic basis of this complex behavior. Here we present a chromosome-level genome assembly for the cribellate orb-weaving spider *Uloborus diversus*. The assembly reinforces evidence of an ancient arachnid genome duplication and identifies complete open reading frames for every class of spidroin gene, which encode the proteins that are the key structural components of spider silks. We identified the two X chromosomes for *U. diversus* and identify candidate sex-determining genes. This chromosome-level assembly will be a valuable resource for evolutionary research into the origins of orb-weaving, spidroin evolution, chromosomal rearrangement, and chromosomal sex-determination in spiders.

Keywords

Arachnid, Spider, Genome, Uloborid, Cribellate, Spidroin

Background

Spiders are among the most successful and diverse terrestrial predators on Earth. Almost 400 million years of evolution has produced more than 50,000 extant spider species representing 128 families that are distributed over every continent except Antarctica[1]. The success of these animals is due in part to the diversity of behaviors that have evolved to capture prey in different environments[2]. Many spiders attack their prey by physically grabbing and immobilizing them with venom and use their silk exclusively for egg sacs. Others use silk to line their burrows, or construct webs of varying geometry and composition to detect or entrap prey. The diversity of

web use correlates with a diversity of spidroin proteins that form silk, as well as the glands that produce these proteins[2–6]. Spiders such as orb-weavers alternate between glands depending upon the web feature they are constructing. For example, load bearing parts of the web such as the radii are composed of major ampullate silk that has high tensile strength, whereas the anchors are made up of pyriform silk which is sticky and amorphous[5].

Remarkably, the orb web is not restricted to a single monophyletic group, but is observed in two lineages that diverged 250 million years ago, leading to considerable debate about its evolutionary origins[3,7–9] (**Figure 1**). Araneoidea is the largest superfamily of orb weavers, which have evolved adhesive aggregate spidroins that are used in the capture spiral to adhere prey to the web[4,10–13]. However, uloborids also build orb webs, but use a more ancient cribellate spidroin in their capture spiral to immobilize prey[14–17]. In addition to Uloboridae, other families such as Deinopidae, and Oecobiidae + Hersiliidae (UDOH grade in **Figure 1**) also build orb-webs, but with more derived behavioral and structural characteristics[18]. Orb-weaving is an innate behavior, with discrete stages of web construction that are shared between araneoid and non-araneoid orb-weavers[19]. When exposed to neuroactive compounds, the behaviors within specific stages are altered, indicating that the neuronal targets of these compounds are more important for certain stages than others[20–22]. This behavioral paradigm offers an excellent model for understanding not only how complex behaviors can be organized in a small brain[23], but also how such behaviors evolve.

However, a genetic understanding of the evolution of orb weaving behavior is hampered by a lack of sequenced genomes for non-araneoid families. Spider genomes are enormous with high repeat content, making them challenging to assemble[24–26]. While 22 spider genomes have been assembled and made publicly available[24,27–42] (**Table S1**), most are highly fragmented, with 6 assembled to chromosome-scale[29,32,35,37,38,42]. Of the 22 genomes, only 7 represent the Araneoidea[28,29,32,39] or ecribellate orb-weavers, 2 of which have been assembled to

chromosome- scale[29,32]. There are no published genomes for the UDOH clade, and only 1 genome represents a member of the cribellate RTA clade (**Figure 1B**)[43]. Chromosome-level assemblies are essential for understanding evolutionary divergence and identifying sites of chromosomal reorganization that play roles in adaptation and speciation.

Spiders have multiple sex chromosomes, with ♂X_1X_2/♀$X_1X_1X_2X_2$ being the most common sex determination observed. Sex chromosome dosage compensation has evolved multiple times, but all known genetic mechanisms are for single sex chromosome systems. A molecular understanding of dosage compensation in spiders is lacking, in part due to a paucity of sex-associated genetic loci.

Uloborus diversus (**Figure 1A**) of the UDOH clade, is a cribellate orb weaving spider native to the desert Southwest in the United States[44] and an important model for understanding the evolution of spidroins and orb-weaving[43]. The existence of a non-araneoid orb-weaving genome is crucial for addressing the evolutionary origins of orb-weaving. Recent work has demonstrated the utility of this species as a model system for understanding orb-weaving behavior[23]. This, combined with the potential to compare both behaviors and their genetic underpinnings across divergent species of orb-weavers offers a rich opportunity to understand the underlying genetics that encode this behavior, and whether orb-weaving behaviors are conserved or convergently evolved. Here, we report a high-quality, chromosome-scale draft genome assembly of *Uloborus diversus* (NCBI: txid327109), as well as a complementary transcriptome assembly and gene annotations. This genome enabled the identification of full >10kb spidroin genes, as well as the identification of sex chromosomes for this species. This chromosome-level assembly will be a valuable resource for evolutionary research into the origins of orb-weaving, spidroin evolution, chromosomal rearrangement, and chromosomal sex-determination in spiders.

Data Description

Genome Sequencing

We sequenced and assembled a high-quality, chromosome-scale genome assembly for *Uloborus diversus* using a hybrid approach that leveraged the complementary benefits of multiple technologies. The genome of *U. diversus* contains long regions of low-complexity sequence, which hinders assembly using short-reads alone, as well as extremely long protein-coding genes, which makes long-reads necessary for a reference-quality assembly[24–29]. Illumina sequencing provides high sequence fidelity but short read lengths, while ONT sequencing provides long read lengths, useful for scaffolding and spanning long, low complexity regions, but lower sequence fidelity[45]. PacBio HiFi sequencing provides an excellent combination of long read lengths and high sequence fidelity, however, we were able to produce multiple megabase-long reads with ONT, which is not possible with PacBio. Each of these sequencing technologies provided unique advantages for improving the overall assembly.

To limit genetic variation, we used sequencing data from only 5 unmated female spiders in our assembly. We used Illumina to obtain high fidelity read data with from a single female spider, generating 795M 150 bp read pairs totaling 119.3 Gb. Because Illumina short reads are not sufficiently long to span long, highly repetitive regions encountered in spider genomes, we sequenced 3 ONT libraries, each from a single female, generating 14.7M reads totaling 98.4 Gb, with a read N50 of 6.7 kb. To obtain long sequencing reads with high sequence fidelity, we also sequenced a single adult female using PacBio HiFi, generating 35M subreads totaling 412.8 Gb with a read N50 of 12.8 kb, which yielded 2M consensus reads totaling 26 Gb with a read N50 of 13.0 kb. To investigate the sex determination system in *U. diversus*, we also generated an Illumina

library from a single adult male, producing 937M 50 bp read pairs totaling 46.8 Gb. Sequencing library statistics are available in **Table 1**.

Genome Size, Heterozygosity, and Coverage Estimation

To assess the size and heterozygosity of the genome, we used Jellyfish[46] to count the frequency of canonical 21-mers in our adult female Illumina sequencing reads and used the 21-mer distribution as input to GenomeScope[47]. The resulting model estimated a genome size of 1.98 Gb with a heterozygosity of 1.38% and 50.2% of the genome occurring as unique sequence (**Figure 2**), similar to other spider genomes (**Supplementary Table S1**)[24,27–34]. Given this genome size, our Illumina sequencing yielded 63x coverage, our ONT sequencing yielded 68x coverage, and our PacBio HiFi yielded 208x in raw read coverage and 13x in consensus read coverage (**Table 1**).

Table 1. Summary of Sequencing Library Statistics

Library Type	Instrument	Mean Read Length	Number of Reads or Read Pairs	Bases Sequenced	Coverage (X)[a]
Illumina	NovaSeq 6000	2 x 150 bp	795 million (female)<br>937 million (male)	119.3 Gb (female)<br>46.8 Gb (male)	60 (female)<br>24 (male)
ONT	PromethION	6.7 kb	14.7 million	98.4 Gb	50
PacBio	Sequel II	12.8 kb (subreads)<br>13.0 kb (consensus)	34.9 million (subreads)<br>2.0 million (consensus)	412.8 Gb (subreads)<br>26.2 Gb (consensus)	208 (subreads)<br>13.1 (consensus)
Chicago Hi-C	HiSeq X	2 x 150 bp	286 million	85.8 Gb	21b<br>43c
Dovetail Hi-C	HiSeq X	2 x 150 bp	537 million	161.1 Gb	1,009b<br>81c

[a] Based on in silico genome size estimate of 1.98 Gb by *k*-mer analysis with GenomeScope 2.0

[b] Physical coverage, defined as the number of read pairs that span a base pair

[c] Sequence coverage, defined as number of times a base pair is directly observed in sequencing data

Karyotype of *U. diversus*

To infer the expected number of pseudo-chromosomes in our final assembly, we determined the number of chromosomes in *U. diversus* using metaphase karyotyping. Mitotic chromosome spreads from developing embryos displayed two distinct patterns of chromosome number; either 18 or 20 (**Figure 3A**), consistent with ♂X_1X_2/♀$X_1X_1X_2X_2$ sex determination, which is the most common form of sex determination observed in spiders[48]. Thus, *U. diversus* appears to have 8 autosomes and 2 sex chromosomes.

***De novo* Nuclear Genome Assembly**

First, we used MaSuRCA[49,50] to produce an initial assembly (*U. diversus* v.1.0) using Illumina short-read data scaffolded by ONT long-read data, consisting of 68,259 scaffolds spanning 3.22 Gb. The scaffold N50 was 98,014 bp and the scaffold L50 was 6,558 (**Table 2**), with 94.7% of complete BUSCOs (**Table 3**). The inferred redundancy accounts for the significant increase in the length of the assembly compared to the expected genome size. High heterozygosity leads to alternative haplotypes that can often be misassembled into their own contigs.

Next, we used Rascaf[51] to improve continuity and ordering of scaffolds in the initial MaSuRCA assembly. Rascaf uses paired-end RNA-seq reads to improve the contiguity of gene models and scaffolds. We observed a modest improvement, reducing the number of

scaffolds from 68,259 to 63,265 with the scaffold N50 increasing from 98,014 bp to 108,431 bp and the scaffold L50 decreasing from 6,885 to 5,994, with no change in the assembly span (**Table 2**). However, despite identifying 95.4% of BUSCOs, 22.5% were duplicated (**Table 3**). This, combined with the large span of the genome, indicated a high degree of redundancy in the assembly.

Table 2. Summary of *Uloborus diversus* Draft Genome Assembly Statistics

	U. div v.1.0		*U. div* v.1.1		*U. div* v.1.2		*U. div* v.1.3		*U. div* v.2.0		*U. div* v.3.0		*U. div* v.3.1	
	**Contigs**	**Scaffolds**	**Contigs**	**Scaffolds**	**Contigs**	**Scaffolds**	**Contigs**	**Scaffolds**	**Contigs**	**Scaffolds**	**Contigs**	**Scaffolds**	**Contigs**	**Scaffolds**
Total Length	3,218,774,062		3,218,805,127		2,798,889,957		2,802,061,857		2,122,354,655		2,151,304,433		2,151,890,133	
Number of Contigs / Scaffolds	68,581	68,259	68,581	66,417	50,738	48,632	55,142	21,317	9,997	9,734	7,467	7,197	7,713	1,586
Largest Contig / Scaffold	3,987,394	3,987,394	3,987,394	6,446,995	3,987,394	6,446,995	3,987,394	367,325,988	4,617,239	4,617,239	5,877,357	5,877,357	5,877,357	272,330,431
Mean Contig / Scaffold Length	46,933	47,155	46,933	48,463	55,162	57,552	50,756	131,447	212,298	218,035	288,107	298,916	278,918	1,356,803
Median Contig / Scaffold Length	21,841	21,923	21,841	21,546	23,184	22,648	24,516	12,994	137,429	218,035	166,676	175,879	168,537	94,191
Smallest Contig / Scaffold	1,106	1,667	1,106	1,667	1,106	1,667	10	312	10,045	10,045	10,045	10,045	380	10,220
N10 / L10	558,595 / 374	564,850 / 373	558,595 / 374	686,213 / 308	613,610 / 302	744,166 / 249	432,729 / 4,437	367,325,988 /1	1,100,324 / 132	1,100,324 / 132	1,705,719 / 86	1,715,742 / 84	1,564,267 / 91	272,330,431 / 1
N20 / L 20	330,632 / 1,135	332,714 / 1,131	330,632 / 1,135	395,570 / 943	375,534 / 894	452,633 / 744	271,565 / 1,278	289,477,137 / 2	711,683 / 383	713,631 / 382	1,147,222 / 244	1,163,865 / 241	1,032,642 / 237	221,850,441 / 2
N30 / L30	214,753 / 2,358	215,989 / 2,348	214,753 / 2,358	252,183 / 1,983	252,860 / 1,816	296,524 / 1,521	192,451 / 2,518	264,078,423 / 3	516,155 / 741	518,859 / 736	826,976 / 467	844,177 / 461	763,983 / 509	218,885,758 / 3
N40 / L40	114,360 / 4,189	145,056 / 4,171	114,360 / 4,189	166,010 / 3,572	178,542 / 3,143	205,473 / 2,661	139,664 / 4,234	243,450,885 / 4	401,815 / 1,210	404,285 / 1,205	636,610 / 763	648,167 / 753	587,015 / 831	185,519,777 / 4
N50 / L50	97,524 / 6,913	98,015 / 6,885	97,524 / 6,913	108,431 / 5,994	126,236 / 5,019	142,390 / 4,302	103,046 / 6,850	241,033,576 / 5	326,127 / 1,798	328,082 / 1,789	487,746 / 1,150	496,769 / 1,133	452,781 / 1,250	185,519,777 / 5
N60 / L60	64,898 / 10,992	65,255 / 10,942	64,898 / 10,992	69,185 / 9,724	88,433 / 7,678	97,899 / 6,679	75,059 /9,775	217,901,867 / 7	259,595 / 2,527	261,845 / 2,511	380,517 / 1,651	387,963 / 1,626	359,368 /1,784	172,099,698 / 7
N70 / L70	42,949 / 17,131	43,310 / 17,041	42,949 / 17,131	44,720 / 15,580	60,079 / 11,529	64,377 / 10,226	53,001 / 14,221	213,666,783 / 8	201,138 / 3,460	204,029 / 3,433	292,644 / 2,296	298,089 / 2,258	280,097 / 2,463	161,310,338 / 8
N80 / L80	28,594 / 26,353	28,760 / 26,208	28,594 / 26,353	29,173 / 24,561	38,159 / 17,400	39,689 / 15,782	35,344 / 20,692	183,225,947 / 9	148,105 / 4,686	151,467 / 4,638	206,980 / 3,169	211,349 / 3,116	200,858 / 3,369	159,483,530 / 9
N90 / L90	18,127 / 40,511	18,198 / 40,294	18,127 / 40,511	18,295 / 38,527	21,027 / 27,290	21,374 / 25,391	20,312 / 31,107	37,840 / 1,399	97,979 / 6,436	100,002 / 6,354	127,987 / 4,484	131,815 / 4,398	126,330 / 4,713	9,355,840 / 14
N100 [illegible]00	1,106 / 68,581	1,667 / 68,259	1,106 / 68,581	1,667 / 66,417	1,106 / 50,738	1,667 / 48,632	10 / 55142	312 / 21,317	10,045 / 9,997	10,045 / 9,734	10,045 / 7,467	10,045 / 7,197	380 / 7,713	10,220 / 1,586
Gaps	322		2,164		2,106		33,825		263		270		6,127	
Ns	32,200		63,265		57,963		3,229,863		6,049		6,749		592,449	
GC Content (%)	33.78		33.78		33.72		33.72		33.83		33.82		33.82	

U. div v.1.0 is the MaSuRCA assembly
U. div v.1.1 is the MaSuRCA assembly with further scaffolding using Rascaf
U. div v.1.2 is the MaSuRCA assembly with Rascaf and with reduction of redundancy using Pseudohaploid
U. div v.1.3 is the MaSuRCA assembly with Rascaf and Pseudohaploid further scaffolded using Chicago and Dovetail Hi-C
U. div v.2.0 is the PacBio IPA assembly
U. div v.3.0 is the MaSuRCA assembly and PacBio IPA assembly merged using MaSuRCA
U. div v.3.1 is the merged MaSuRCA/IPA assembly further scaffolded using Dovetail Hi-C

Table 3. Summary of Draft Genome Assembly BUSCO Scores

	v.1.0	*v.1.1*	*v.1.2*	*v.1.3*	*v.2.0*	*v.3.0*	*v.3.1*
Complete	94.7	95.4	95.1	97.0	92.4	94.1	94.8
Single Copy	70.9	72.9	78.4	84.6	82.1	82.9	85.2
Duplicated	23.8	22.5	16.7	12.4	10.3	11.2	9.6
Fragmented	2.3	1.9	2.0	1.2	1.7	1.3	1.0
Missing	3.0	2.7	2.9	1.8	5.9	4.6	4.2

To filter out redundant heterozygous contigs we used Pseudohaploid[52]. Pseudohaploid filters suspected homologous contigs and selects a single representative contig where high rates of heterozygosity prevent assemblers from appropriately identifying haplotypes. We reduced the number of scaffolds by 23.1%, with an increase in scaffold N50 and a decreased span from 3.22 Gb to 2.8 Gb (**Table 2**) and a drop in duplicated BUSCOs to 16.7% (**Table 3**). This suggests that Pseudohaploid was able to accurately collapse much of the redundant sequence attributable to alternative haplotypes.

To further improve our assembly, we sequenced using PacBio HiFi and assembled the resulting HiFi reads with PacBio's IPA (Improved Phased Assembly) pipeline, resulting in a substantial decrease in the new assembly span, from 2.8 Gbp to only 2.1 Gbp. The number of scaffolds in this assembly was only 9,734, a remarkable improvement. The scaffold N50 in the IPA assembly was 328,082 bp and the scaffold L50 in the IPA assembly was 1,789 (**Table 2**). The BUSCO score for the IPA assembly indicated that this assembly contained 92.3% of BUSCOs complete (83.7% in single copy, 9.3% duplicated) (**Table 3**).

We then used SAMBA[53] to merge the previous MaSuRCA assembly with the IPA assembly.

SAMBA used scaffolds from the MaSuRCA assembly to merge the scaffolds in the IPA assembly. The new assembly had a length of 2.1 Gbp from only 7,197 scaffolds, with a scaffold N50 of 496,769 bp (**Table 2**), and 94% complete BUSCOs (**Table 3**).To generate a chromosome-level assembly, we used HiRise to scaffold the IPA+MaSuRCA assembly with a Dovetail Hi-C library[54]. Scaffolding did not change the amount of sequencing in the assembly; however, the total number of scaffolds was reduced by 78% to only 1,586 scaffolds, with a remarkable improvement in scaffold N50, which increased to 185,519,777 bp in the Hi-C scaffolded assembly (**Table 2, Figure 3C**). Most importantly, 88% of the total assembly was represented by 10 large scaffolds that comprise 1.9 Gbp (**Figure 3B**), matching the expected number of chromosomes (**Figure 3A**). The BUSCO score for the final assembly showed 94.8% of the BUSCOs were complete (with 85.2% in single copy, 9.6% duplicated) (**Table 3**). Our final chromosome-level genome assembly statistics are consistent with previously published spider genomes, with 10 scaffolds representing the 10 chromosomes and high scaffold N50[24,27–34] (**Supplementary Table S1**). We will refer to these 10 scaffolds as pseudochromosomes.

Repeat Annotation

To characterize repetitive sequences, we constructed a species-specific repeat library using RepeatModeler2[55] This library was used in conjunction with the RepBase RepeatMasker Edition[56] database for masking the genome. RepeatMasker analysis of the combined *U. diversus* and RepBase repeats masked 66.6% of the final *U. diversus* genome assembly. Many (29.27%) of the repetitive regions were unclassified; however, DNA transposons accounted for a similar proportion (22.73%). Retroelements accounted for a much smaller proportion (7.7%). Total interspersed repeats account for 59.7% and simple repeats cover 2.61% of the genome (**Table 4**). The disparity between the GenomeScope estimation of 49.8% repetitive sequence and the RepeatMasker estimation of 66.6% suggests that the repeat content may be underestimated by

GenomeScope. Therefore, the genome size may also be underestimated by GenomeScope. Because the length of the *U. diversus* genome is slightly less than the prediction by GenomeScope, this possibility suggests that the length of the assembly may more closely represent the true length of the *U. diversus* genome than the GenomeScope prediction. The repeat content is typical of spider genomes (**Supplemental Table S2**).

Transcriptome Sequencing and Assembly

To identify protein-coding genes, we assembled a transcriptome. To capture a wide range of transcripts, we extracted RNA from spiders at multiple developmental stages and from male and female adults. We produced an Illumina short-read sequencing library from: a whole adult female, a whole adult male, the dissected prosoma (cephalothorax) from an adult female, the dissected opisthosoma (abdomen) from the same adult female, the dissected prosoma from an adult male, the dissected opisthosoma from the same adult male, the pooled legs from the dissected male and female, a single 4th instar female, and approximately 30 pooled 2nd instars. 302M read pairs were generated, totaling 45.4 Gbp.

Table 4. Summary of the Repeat Content of the Uloborus diversus Draft Genome Assembly

Type of Element	Number of Elements	Total Length	Percent of Assembly
Retroelements	311,947	165,713,554	7.70
SINEs	87,037	46,791,087	2.17
Penelope	29,911	11,105,124	0.52
LINEs	151,953	66,189,386	3.08
CRE/SLAC	0	0	0.00
L2 / CR1 / Rex	31,294	18,030,662	0.84
R1 / LOA / Jockey	17,662	9,587,620	0.45
R2 / R4 / NeSL	0	0	0.00
RTE / Bov-B	40,080	15,062,698	0.70
L1 / CIN4	21,465	5,682,132	0.26

LTR elements	72,957	52,733,081	2.45
BEL / Pao	10,210	8,094,671	0.38
Ty1 / Copia	24,043	10,565,700	0.49
Gypsy / DIRS1	27,837	29,214,338	1.36
Retroviral	10,867	4,858,372	0.23
DNA transposons	1,510,089	489,113,225	22.73
hobo-Activator	545,025	164,842,101	7.66
Tc1-IS630-Pogo	428,233	150,644,883	7.00
En-Spm	0	0	0.00
MuDR-IS905	0	0	0.00
PiggyBac	11,411	4,281,311	0.20
Tourist / Harbinger	6,573	2,466,806	0.11
Other	2,942	1,822,173	0.08
Rolling-circles	229,864	88,460,474	4.11
Unclassified	2,482,847	629,886,344	29.27
Total Interspersed Repeats		1,284,713,123	59.70
Small RNA	20,797	5,420,377	0.25
Satellites	0	0	0.00
Simple repeats	529,329	56,254,893	2.61
Low complexity	67,980	3,208,338	0.15
		**Total:**	66.58

We used Trinity[57] to assemble a genome-guided transcriptome. We then used TransDecoder[57] to find coding regions within our transcripts. We included homology searches to known proteins using both BLAST (Basic Local Alignment Search Tool)[58,59] and Pfam[60] searches. We assessed the BUSCO score of the long ORFs predicted by TransDecoder, finding that 90.9% of the BUSCOs were present and complete, with 54.8% single copy and 36.1% duplicated, with 1.5% present but fragmented and 7.6% missing. (**Table 3**).

Protein-Coding Gene Annotation

For protein coding gene annotations, we used BRAKER2[61,62] with our RNAseq data and homology evidence using a custom library of spider proteins obtained from NCBI (**Supplemental Table S3**). The number of predicted genes in the final *U. diversus* assembly was 44,408; with 40,466 models predicted on the 10 pseudochromosomes (**Table 5**), with 86.7% of complete BUSCOs. To functionally annotate these genes, we used Interproscan[63,64] to annotate the longest CDS for each gene. 30,911 models were assigned a domain or function from one or more of the databases used (**Table 6**).

Table 5. Summary of Annotation Statistics for the Uloborus diversus Draft Genome Assembly

Number of Gene Models	45,762
Minimum Gene Model Length (bp)	60
Maximum Gene Model Length (bp)	408,348
Average Gene Model Length (bp)	16,764
Number of Exons	222,483
Average Number of Exons per Gene Model	5
Average Exon Length (bp)	237
Number of Transcripts	47,540
Average Number of Transcripts per Gene Model	1
Number of Gene Models < 200 bp	37

Table 6. Summary of Interproscan Results

Database	Total Hits	Individual mRNAs with Hits
CDD	11047	6560
Coils	9254	5947
Gene3D	34377	16424
Hamap	270	260
MobiDBLite	44028	14046
PANTHER	46497	20941
Pfam	35843	19491

PIRSF	916	736
PRINTS	16380	3134
ProSitePatterns	8222	3898
ProSiteProfiles	23252	9922
SFLD	120	62
SMART	25638	7561
SUPERFAMILY	26766	15559
TIGRFAM	821	736

Non-Coding RNA Annotation

We used tRNAscan-SE[65,66] to annotate transfer RNAs. We found 3,084 tRNAs coding for the standard 20 amino acids and 14 tRNAs coding for selenocysteine (TCA) tRNAs. We found 21 tRNAs with undetermined or unknown isotypes, 537 tRNAs with mismatched isotypes, and 57,824 putative tRNA pseudogenes. We identified no putative suppressor tRNAs. We used Barrnap[67] to annotate ribosomal rRNAs. We found 114 rRNAs, of which 100 were located on the 10 pseudo-chromosomes. These included: 6 copies of the 18S subunit, with 4 on pseudochromosomes; 83 copies of the 5S subunit, with 81 on pseudochromosomes; 6 copies of the 5.8S subunit, with 2 on pseudochromosomes; and 19 copies of the 28S subunit, with 13 pseudochromosomes.

Mitogenome Assembly

Animal mitochondrial genomes comprise 37 genes: 13 protein coding genes, 22 tRNAs, 2 rRNAs, and at least one control region[68]. We assembled the mitochondrial genome sequence with NOVOplasty[69] using the adult female Illumina DNA seq data and each of the mitochondrial genome sequences listed in **Supplemental Table S4** as sources for seed sequences[70–83]. Each run produced the same single, circularized 14,737 bp mitochondrial sequence, consistent with the expected size for an arachnid mitochondrial sequence[68]. We annotated the sequence with the MITOS2 web server[84] and found all 13 of the expected protein coding genes, 20 of 22

tRNAs, 2 rRNAs, and the control region (**Fig. 3E**). All identified tRNAs were truncated and lacked T-arms, which is unique to spiders and has been observed in other species[75,77,85,86].

Identification and Analysis of Spidroins

Spidroins are a unique class of proteins that are the primary components of spider silk. While all spiders produce silk, spidroins have evolved for different uses in web-making. Orb-weavers in particular evolved several silk glands that each produce a different repertoire of spidroins to make different silks with varying utility. Several ecribellate Araneidae spidroins have been sequenced, and many of these spidroins are also made by cribellate orb-weavers such as *U. diversus*. However, ecribellate spiders evolved a unique type of hydrated flageliform silk for their capture spiral, whereas cribellate spiders such as *U. diversus* use a dry cribellate silk in their capture spirals.

Spider dragline silk has the strongest stress and strain capabilities of any known substance. Interest in silk properties extends beyond their evolved use, as silk has many potential human applications in both industry and medicine[87–94]. However, the genetic characterization of spidroins is often challenging due to their exceptional length (coding regions >5 kb) and high repeat content[25]. The annotation of these genes is difficult and often fragmented because reads rarely span the entire length of these genes. With the exceptional contiguity and read depth of our assembly, due to the diversity of sequencing technologies employed, we identified the entire open-read frames of all major spidroins in the *U. diversus* genome.

We found 10 full-length candidate sequences, including at least one candidate for each of the seven types of spidroin used by cribellate orb-weavers. There were no gaps in the assembly interrupting any of our candidate spidroin sequences. We performed read mapping to validate the continuity of each full-length sequence and ensure that the predicted sequences were not

chimeric. In 8 cases, including 3 minor spidroin (MiSp) candidates, a major spidroin (MaSp) MaSp-1 candidate, 2 MaSp-2 candidates, a tubuliform spidroin (TuSp) candidate, and an aciniform spidroin (AcSp) candidate, the full length of the predicted genomic region was spanned entirely by at least one HiFi consensus read. In the remaining 3 cases, consisting of a pyriform spidroin (PySp) candidate, a cribellate spidroin CrSp candidate, and a pseudoflagelliform spidroin (Pflag) candidate, no more than 2 HiFi reads were necessary to span the entirety of the predicted genomic region, and in each of these cases there was sufficient depth and overlap in the reads to call the region with high confidence (see **Supplemental Figure S1**). The length of the coding regions for the spidroins ranged from 5.5 kb to 20 kb. This is consistent with expectations of full-length sequences found in other spiders[27,28,95]. Only in the case of MaSp-1 were we unable to call a complete, full-length sequence. The finalized spidroin annotations included only two spidroins with multiple exons: CrSp and MaSp-1; however, the structure of MaSp1 remains unclear. All other spidroins were found to be single exon sequences. While most single-exon genes tend to be small highly expressed proteins such as histones, spidroins are a rare exception. The single exon structure of spidroin genes has been noted in other species[26,95–102].

Aciniform spidroin (AcSp)

Aciniform silk is one of the toughest spider silks, and is typically used for wrapping prey[103]. A single exon for AcSp was identified on Chromosome 7 (**Figure 4A** and **Table 7**), consistent with the sequences in the Araneid orb-weaving spiders *Araneus ventricosus*[104] and *Argiope agentata*[105], as well as the cobweb spider *Latrodectus hesperus*[26]. Our confidence in this sequence is high since the complete sequence was spanned entirely by multiple PacBio HiFi reads. In the repetitive region, we found 13 iterated repeats of a 357 amino acid motif, and a 14th partial repeat (**Supplemental Figure S1**). As with previous reports on the structure of AcSp[26,104–106], we also found that the repeats are remarkably well-homogenized. After

removal of the signal peptide between Ser-23 and Arg-24, the remaining N-terminal domain secondary structure includes 5 alpha-helices, and a C-Terminal domain consisting of 4 alpha helices which is consistent with the structure found in other AcSps[104].

Pseudoflagelliform spidroin (Pflag)

The capture spiral of an orb-web is a composite of two types of silk[107]. For cribellate spiders such as *U. diversus*, the core fiber is the pseudoflagelliform silk made up of pseudoflagelliform spidroin (Pflag). When produced, this core fiber is coated with finely brushed cribellate silk which provides adhesive properties to the capture silk (see Cribellate spidroin).

We found a single candidate for Pflag on Chromosome 7 (**Figure 4A** and **Table 7**). We determined that the internal structure consists of 91 repeats, each of which range between 39 and 70 aa in length and are composed of two parts: a glycine-poor spacer, which is usually either 7 or 12 aa, followed by a glycine-rich repeat with variations on the motif *PSSGGXGG*. The final repeat motif in each module always terminates in a proline.

Table 7. Summary of Spidroins

Spidroin	Gene Length (bp)	CDS Length (bp)	Protein Length (aa)	N-terminal Length (aa)	C-terminal Length (aa)	Signal Peptide Stop
Aciniform	14,907	14,907	4,968	148	109	Ser-23
Pseudoflagelliform	15,546	15,546	5,181	195	96	Gly-29
Cribellate	20,198	20,115	6,704	874	296	Gly-23
Major Ampullate 2a	7,317	7,317	2,438	165	100	Gly-25
Major Ampullate 2b	9,141	9,141	3,046	195	109	Gly-25
Minor Ampullate 1a	5,523	5,523	1,840	242	95	Gly-23
Minor Ampullate 2a	6,255	6,255	2,084	254	99	Gly-23
Minor Ampullate 2b	6,432	6,432	2,143	254	98	Gly-23
Pyriform	13,179	13,179	4,392	168	266	Gly-23
Tubuliform	10,146	10,146	3,381	179	346	Ala-25

Cribellate spidroin (CrSp)

Cribellate silk is produced by numerous silk glands with hundreds to thousands of spigots in the cribellum. These numerous fibers are combined into a single silk which is combed into a "wooly" silk by calamistra located on the posterior legs. This wooly silk soaks into the waxy cuticle of insects by means of van der Waals interactions and hygroscopic forces and is used as the capture silk by cribellate orb-weavers[108]. No full-length sequence for CrSp has been reported to date, although partial spidroin sequences have been reported for some CrSps in *Tengella perfuga*[109] and several *Octonoba* species[95]. The whole genomic length of the *CrSp* locus on Chromosome 10 is 20,195 nt (**Figure 4A** and **Table 7**). The *U. diversus CrSp* gene was predicted to be a 2-exon gene, with a single 83 nt intron, which is consistent with what we found by manual inspection. While the entire *CrSp* locus was not spanned by single HiFi reads, we are still confident in the sequence produced, since no more than 2 reads were required to span the entire sequence. The N-terminal region of the predicted protein product consists of 874 aa and the C-terminal region consists of 296 aa (**Table 7**). The long N-terminal domain is consistent with that found in *Octonoba* spp, which were also found to have coding regions more than 2 kbp[95]. We found an internal region that consists of variations on 4 repetitive motifs, with the first half of the sequence made up of motifs 1 and 2 alone, and the second half of the sequence including all 4 motifs. Motifs 1 and 3 are similar, whereas motifs 2 and 4 are distinct from each other motif (**Supplemental Figure S1)**.

Major ampullate spidroin (MaSp)

Draglines are produced by the major ampullate gland which produces two major ampullate spidroins (MaSp1 & MaSp2). This silk has extremely high tensile strength and elasticity and is commonly used for the primary load-bearing parts of the web such as the frame and radii. It is also the primary silk produced by spiders when they are navigating their environment[5]. We found

three candidates for major ampullate spidroin (MaSp). Based on previous work that identified multiple distinct classes of MaSps, we were able to assign one of our candidate sequences to the MaSp-1 class and the other two candidates to the MaSp-2 class. All three MaSp candidates are on Chromosome 6, although the MaSp-1 locus was located distantly from the two MaSp-2 loci (**Figure 4A**).

The MaSp-1 candidate is the single spidroin sequence we were not able to call as a complete sequence. In our annotation, the sequence appears as a two-exon gene, with the 5' sequence and 3' sequence found in different reading frames; however, a close inspection of the data suggests that this is not likely to be correct. We found instead that there is a large region to which the PacBio HiFi reads mapped poorly. There is consensus between the reads that indicates sequence found in the reference assembly that is not found in the reads. However, it is not clear from inspection exactly where the boundaries should be called for this region. This is likely an artifact of the assembly, since the reference was assembled from polymerase-based sequencing which is susceptible to polymerase-slippage.

The first MaSp-2 candidate, MaSp-2a, is a single exon sequence. We found that there were two distinct regions. Interestingly, the first repetitive region, which is 958 aa in length, contains mostly *GPGPQ* motifs reminiscent of the *GPGPX* motifs found in the MaSp-4 sequence recently reported in *Caerosris darwini*, but not elsewhere in the known catalog of spidroins (Garb, *et al*., 2019). The second repetitive region, which is 1,215 aa long, contains runs of poly-A and *GPX*, although *GPGPQ* repeats are also found less frequently in this region.

The second MaSp-2 candidate, MaSp-2b is also a single exon sequence. In the repetitive region, GPGPQ occurs in a few instances, but is relatively rare compared to MaSp-2a. Alternating runs of polyalanine and variations on the motif *GSGPGQQGPGQQGPGGYGPG* characterize the repetitive region. Unlike the case of the first MaSp-2 candidate, MaSp-2b does not have two distinct repetitive regions.

Minor ampullate spidroin (MiSp)

Minor ampullate silk has lower strength, but greater extensibility, and is composed of spidroin made by the minor ampullate gland. While it is commonly used for the construction of the auxiliary spiral in orb-weavers, it is used for prey wrapping by cob-weavers[110]. We found three candidates for minor ampullate spidroin (MiSp). All three MiSp loci were located near one another on Chromosome 1 (**Figure 4A**).

The first candidate, MiSp-1 is a single exon sequence (**Table 7**). There are three repetitive regions in MiSp-1, separated by short spacers. Previous work in *Araneus ventricosus* and the cobweb weaving spiders *Latrodectus hesperus*, *L. tredecimguttatus*, *L. geometricus*, *Steatoda grossa*, and *Parasteatoda tepidariorum* has suggested that MiSp length and sequence are conserved[110,111]; however, while the spacers we observed shared some sequence similarities, such as the presence of serine, threonine, and valine residues, the lengths of the spacers observed in *U. diversus* are much shorter.

The second and third candidates, MiSp-2a and MiSp-2b, shared a nearly identical amino acid composition, which was slightly different from that of MiSp-1. Both are single exon sequences (**Table** 7). Their hydrophobicity profiles are also slightly different from MiSp-1.

Pyriform spidroin (PySp)

Pyriform silk serves as an adhesive compound used to adhere silk lines to one-another, or to substrate that holds the web[5]. We found one single exon candidate pyriform spidroin (PySp) on Chromosome 1 (**Figure 4A**), which is consistent in size with a prior PySp sequence reported from *Araneus ventricosus*[101]. We found that the internal repetitive region was preceded by a Q-rich N-linker region. 19 tandem repeat motifs, ranging from 188 - 196 aa were found.

Tubuliform spidroin (TuSp)

Tubuliform silk is used to encase the egg sac and is spun from tubuliform glands. We found a single candidate for tubuliform spidroin (TuSp) on Chromosome 2 (**Figure 4A** and **Table 7**) which is a single exon We found an internal region that was composed of 10 repeats, ranging from 262 aa - 302 aa in length. This is consistent with other reported TuSp repeats, which have been observed between 176 and 375 residues, although it seems that the typical TuSp module is repeated 15 to 20 times[102]. The N-terminal region has 3 Cys residues: Cys-21, Cys-52, and Cys-132. Other TuSp N-terminal sequences have been reported with 2 Cys residues[102], however Cys-21 is expected to be removed during signal peptide cleavage. After cleavage, the N-terminal domain contains five predicted alpha helices. Cys-52 and Cys-132 are found in alpha helix 1 and alpha helix 4 after cleavage, which is also where the AcSp Cys residues are found. This conservation suggests functionality.

Whole Genome Duplication

Gene duplication acts as a primary mode of evolutionary diversification by providing new genetic material that serves as a reservoir for subfunctionalization and neofunctionalization under selective pressure[112–114]. In previous studies in spiders, evidence of whole genome duplication has been reported, including the presence of multiple copies of *Hox* genes[29,36,115,116] and the expansion of silk genes and chemosensory genes[36]. We analyzed the *U. diversus* genome to identify signatures of whole genome duplication.

Evidence for whole genome duplication can be ascribed by assessing the number of *Hox* gene clusters[117]. We used published *Hox* gene sequences from the spider *Parasteatoda tepidariorum* as query sequences for BLAST searches against the *U. diversus* genome, identifying two *Hox* gene clusters on Chromosome 5 and Chromosome 10 (**Figure 4A**), which were found to retain the expected order of *Hox* genes[118]. Each cluster was missing a single *Hox* gene; however, the specific missing gene was different for each of the clusters. Cluster A on Chromosome 5 is missing the *fushi tarazu* (*ftz*) gene sequence, while Cluster B on Chromosome 10 is missing the *labial* gene sequence. Our findings are consistent with the discovery of two *Hox* gene clusters in *P. tepidariorum*[31]. In the genome of *T. antipodiana*, two *Hox* clusters were found[32], including one complete cluster on chromosome 12 which included a copy of all 10 expected *Hox* genes, and a second cluster on chromosome 8 which was found to be missing *abdominal-A*, *Hox3*, *ftz*, and *Ultrabithorax*.The presence of multiple *Hox* clusters in the *U. diversus* genome adds further support to an ancient, ancestral whole genome duplication.

We searched for evidence of synteny between pseudochromosomes using AnchorWave[119]. The initial identification of syntenic blocks was quite ubiquitous across all 10

pseudochromosomes, but with large gaps between mRNAs and/or with very large interanchor distances. To constrain these results, we chose to include only mRNAs where the number of missing mRNAs between anchors was 4 or less. We made this allowance to account for the fact that we expect there to be significant loss of copies of duplicated genes[112,113,120]. This resulted in retaining 196 blocks of at least 2 mRNAs (**Figure 5**). Considering the conservative nature of our analysis, this line of evidence provides further support for an ancient duplication event. However, considerable reorganization has occurred since the duplication event, an observation also made in mammalian genomes, and potentially associated with their successful adaptation to diverse environments[121,122].

We also compared the synteny of our *U. diversus* pseudochromosomes with those from *Argiope bruennichi*, an Araneid orb-weaver, *Meta bourneti* a Tetragnathid orb-weaver, as well as *Dolomedes plantarius* a Pisaurid (**Figure 1**). While the syntenic blocks of some of the chromosomes seem split between multiple chromosomes across the species, certain chromosomes, or pairs of chromosomes, have nearly exclusive synteny between species. *A. bruennichi* chromosomes 6, 7, and 8 share nearly exclusive syntenic blocks with *U. diversus* chromosomes 5, 6, and 4, respectively. This shared synteny with *U. diversus* chromosomes 5 and 6 is also observed for chromosomes 8 and 5 from *M. bourneti*, however U. diversus chromosome 4 is split between M. bourneti chromosomes 1 and 7. *U. diversus* chromosome 1 shares considerable synteny with *A. bruennichi* chromosomes 3-5, while *U. diversus* 2 is biased for shared synteny with A. bruennichi chromosomes 1-2 and 11-12. Even though *D. plantarius* diverged more recently from *U. diversus* (**Figure 1**), there appears to a greater degree of chromosomal rearrangement between these two species. However, two outliers are *U. diversus* 3 and 10 which share nearly exclusive synteny with *D. plantarius* 12 and 5, respectively. The large degree of synteny between *U. diversus* 3 and 10 with *M. bourneti* X1 and X2 is a strong indication that these two chromosomes are the sex chromosomes for *U. diversus*.

Sex Chromosomes

The most common and likely ancestral system of chromosomal sex determination in spiders is ♂X_1X_2/♀$X_1X_1X_2X_2$.[48] However, spiders exhibit a diversity of sex determining systems, with some including Y chromosomes, and others possessing up to 13 X chromosomes[123]. Usually, these sex determining systems are determined by karyotyping[48,123] (**Figure 3**). The genetic basis of sex determination and chromosomal dosage compensation are unknown for spiders. Determining the genetic identity of X chromosomes has been challenging, in part due to significant levels of shared synteny between sex chromosomes and autosomes[48] (**Figure 5**), as well as a paucity of spider genomes with chromosome-level scaffolds. X chromosome scaffolds from more fragmented genomes have been identified by quantifying the relative difference in read depth from sperm with or without the X chromosomes[124]. Recently, the X chromosomes for *A. bruennichi* (also ♂X_1X_2/♀$X_1X_1X_2X_2$) were identified through disparities in read coverage of X chromosomes between males and females[48]. In principle, because males have only one copy of each X chromosome, the average read depth for scaffolds from these chromosomes should be half that of autosomes. To determine the sex chromosome in *U. diversus*, we assembled an Illumina short-read library from a single male spider and mapped the reads onto the 10 assembled pseudochromosomes (**Figure 6**).

While 8 of the 10 pseudochromsomes had a median read depth of 40 ± 2, pseudochromosomes 3 and 10 were outliers, with read depths of 36 and 33, respectively. If these pseudochromosomes were exclusively unique X chromosomes, the expected read depth would have been ~20. However, as observed in other species[48] and our own (**Figure 5**), orthologous autosomal regions should decrease the expected depth disparity. The higher than expected read depth could also be due to mis-assembly of these pseudochromosomes, however very little linkage was observed between pseudochromosomes 3 and 10 in the Hi-C data (**Figure 3**). Despite these

caveats, the lower median read depth in males for pseudochromosomes 3 and 10 is a strong indicator these likely represent the two X chromosomes for *U. diversus*.

Prior work with *Stegodyphus mimosarum* (also ♂X_1X_2/♀$X_1X_1X_2X_2$) identified sex-linked scaffolds based on lower read depth of sperm lacking X chromosomes[124]. When genes identified on these X-linked *S. mimosarum* scaffolds were mapped on to the *U. diversus* pseudochromosomes (**Table 5**), 62% of these genes mapped onto pseudochromosomes 3 and 10 (**Figure 6B**). This large fraction of predicted X-linked genes between two distantly related species of spiders is a strong indicator that not only are pseudochromosomes 3 and 10 likely to be the X chromosomes, but that the genetic composition of these chromosomes has remained fairly stable amongst spiders. Since two X chromosomes were recently identified in *A. bruennichi*, we compared the genetic composition (**Supplemental Table S6**) and synteny between the X chromosomes identified in both species (**Figure 6**). In addition to shared X-linked genes (**Supplemental Table S6, Figure 6B**), *A. bruennichi* scaffolds 9 and 10 appear to share considerable synteny with *U. diversus* pseudochromosomes 3 and 10 (**Figure 6C**), while sharing little synteny with the autosomes of the other species (**Figure 5E**). This also appears to be true of the sex chromosomes X1 and X2 from *M. bourneti*, however this genome is not currently annotated. When chromosomal rearrangements have occurred, they appear to have been confined to rearrangements between sex-chromosomes. The sex chromosomes themselves share little synteny with each other (**Figure 5**), which indicates they are not the result of an ancient duplication, but there appears to be selective pressure to ensure that when chromosomal rearrangements do occur, that they occur between the sex chromosomes. However, some syntenic blocks are shared with autosomes. One of these syntenic blocks is the *Hox* gene cluster located on chromosome 10 for both *U. diversus* and *A. bruennichi*. The presence of a *hox* cluster on a sex chromosome was surprising since these genes play critical roles in development. Therefore, either dosage compensation is needed

in males, or dosage disparity between males and females plays a role in developmental sexual dimorphism.

In insects, the primary sex chromosome dosage sensor is *sex lethal* (*sxl*), which then triggers a cascade of sex-defining signaling events leading to sexually dimorphic expression of genes and/or splice variants. While no *sxl* homologue has been found in spider genomes (including *U. diversus*), other genes involved in sexual dimorphism, such as *doublesex* (*dsx*) are present. Thus, the mechanism spiders use for sensing X:autosome ratio differences remains unknown, but relevant genes are likely shared between *U. diversus*, *A. bruennichi*, and *S. mimosarum*. Of the 534 shared sex-linked genes in these three species, 14 are predicted to be DNA/RNA-binding, and may play a role in sex-determination. The X-linked genes shared between these three species (**Supplemental Table S6**) will be a resource for comparative analysis to identify conserved genes that serve as sex-specifying triggers for spiders. Uncovering how spiders perform sex-linked dosage compensation can not only illuminate how arthropods evolved different sex-determining systems, but also how dosage compensation has evolved independently in numerous animals.

Discussion

Here, we present a high-quality chromosome-level genome and complementary transcriptome assembly of the hackled orb-weaver *Uloborus diversus*. The 2.15 Gbp draft genome assembly comprises 1,586 scaffolds, including 10 pseudochromosomes that contain 1.9 Gbp (88%) of the total assembly, comparable to the estimated genome size (1.98 Gbp) predicted by GenomeScope2 and contains the vast majority of highly conserved orthologs (94.1% complete, with 88.6% complete and in single copy) as estimated by BUSCO. We predicted a total of 44,408 protein-coding gene models with a BUSCO completeness of 86.7%. Despite the aforementioned

technical hurdles, the contiguity and completeness of this assembly, along with the recovery of a complete catalog of full-length spidroin gene sequences, demonstrates the utility of using multiple complementary sequencing technologies for large, repetitive, and highly heterozygous genomes.

The repetitive nature and length of spidroin genes have posed a technical challenge for identifying and reporting full-length sequences. However, it is exactly these qualities that lend spidroins their unique mechanical properties[125–127]; underscoring the need for accurate assemblies. Recent studies leveraging single molecule, long read sequencing technology have predicted longer spidroin sequences than those using PCR approaches[28]. Here, we used ONT and PacBio HiFi reads to achieve a complete catalog of full-length spidroin sequences for *Uloborus diversus*. The ability to recover full-length sequences for this family of genes is an indication of the high quality of the assembly.

All current models of chromosomal dosage compensation are based on single-sex chromosome animals, however multiple sex-chromosome systems exist in both vertebrates and invertebrates[128,129]. Spiders exhibit considerable morphological and behavioral sexual dimorphism that is based on a multiple-sex chromosome system. Understanding the genetic underpinnings of spider sexual development will contribute to a fuller understanding of how chromosomal sex determination can evolve independently in different species. Here we provide evidence for the identities of sex chromosomes in *U. diversus* and leverage this information to identify 14 candidate DNA-binding genes that are shared between three divergent species of spiders.

Our genome will facilitate comparative studies and meets a specific need in the field for a greater representation of genomes from the UDOH+RTA clade that represent nearly half of all known

spider species (**Figure 1**)[43]. We expect that the highly contiguous draft genome and transcriptome datasets we produced for *U. diversus* will serve as a valuable resource for continuing research into the evolution, development, and physiology of spiders, as well as a vital tool to study the genetic basis of orb-weaving behavior. While a handful of spider genomes have been published, all orb-weaving genomes have been from ecribellate Araneid spiders, with no representative genomes from the cribellate families Uloboridae, Deinopidae, Oecobiidae, or Hersiliidae. Improved knowledge of genomes from these families, combined with behavioral and cellular analyses of orb-weaving behavior, will offer a crucial foundation for understanding how and when orb-weaving evolved.

Materials and Methods

Sample Collection and Husbandry

We collected spiders of the species *Uloborus diversus* from the ancestral lands of the Ramaytush, in Half Moon Bay, California, USA. We collected colony founders from a single greenhouse during several trips between 2016 and 2019 and transported them to custom-fabricated habitats in an on-campus greenhouse at Johns Hopkins University. We later transferred experimental animals from the on-site greenhouse to custom-fabricated habitats in the laboratory until required for experiments. We fed all animals alternately *Drosophila melanogaster* or *Drosophila virilis* once per week.

Karyotyping

We soaked embryos soaked in Grace's insect medium (Gibco) containing 0.1% colchicine for 2 hours. We then added an equal volume of hypotonic solution. After 15 min, we transferred the embryos to a 3:1 ethanol:acetic acid solution for 1 hour. After fixing, we transferred embryos to gelatin-coated microscope slides and dissociated them in a drop of 45% acetic acid. We used siliconized coverslips to squash the dissociated tissue and briefly froze them in liquid nitrogen. After removing the slides from LN2, we immediately removed the coverslips with a razor blade and transferred the slides as quickly as possible to 95% ethanol. We then performed a step-down series from 95% ethanol to 70%, 35%, and finally to Grace's insect medium to return the tissue to an aqueous solution. We then transferred the slides to a 1ug/mL DAPI solution. After a 10-minute incubation, we transferred the slides to de-ionized water to rinse and mounted coverslips with a drop of Vectamount (Vector Laboratories, Burlingame, CA, USA).

RNA Extraction, Library Preparation, and Sequencing

We extracted total RNA from multiple samples: a whole adult female, a whole adult male, adult female prosoma and opisthosoma, adult male prosoma and opisthosoma, pooled legs from both the adult female and adult male dissections, a 4th instar female, and approximately 30 pooled 2nd instars. We used the Qiagen RNeasy Mini Kit (Qiagen, Hilden, Germany) to extract total RNA, following the manufacturer's protocol. We estimated the quality and quantity of total RNA using a NanoDrop One Microvolume UV-Vis Spectrophotometer (ThermoFisher Scientific, Waltham, MA, USA). Before library preparation, we also measured the quality, quantity, and fragment length of our total RNA using a TapeStation 4200 System with RNA ScreenTape and reagents (Agilent, Santa Clara, CA, USA). We prepared barcoded, directional, paired-end RNA-seq libraries with the NEBNext Ultra II Directional RNA Library Prep Kit for Illumina using the NEBNext Poly(A)

mRNA Magnetic Isolation Module. We submitted the resulting libraries to the Johns Hopkins Genomics Core Resources Facility to be sequenced on an Illumina HiSeq 2500 Sequencing System with 150 bp paired-end chemistry.

Genomic DNA Extraction, Library Preparation, and Sequencing

Prior to extraction of DNA, we withdrew food for 3 days to minimize the potential contribution of contaminating DNA from dietary sources. We extracted high molecular weight (HMW) DNA using the QIAgen MagAttract HMW DNA kit. Prior to HMW purification, we followed the manufacturer's protocol for disruption/lysis of tissue. We avoided fast pipetting and prolonged vortexing to minimize shearing of DNA. We flash froze adult spiders in liquid N_2 and crushed them with a pellet pestle (Fisher, 12-141-364) in a Protein LoBind tube (Eppendorf, 022431081) containing 220 uL of Buffer ATL. We then added 20 uL Proteinase K and briefly vortexed the sample. We next incubated the sample overnight at 56C with 900 rpm shaking on a ThermoMixer C (Eppendorf, 5382000023). After the overnight incubation, we then briefly centrifuged the sample to spin down condensate on the tube. We next transferred 200 uL of lysate to a fresh 2 mL sample tube and followed the manufacturer's protocol for manual purification of HMW DNA from fresh or frozen tissue. We estimated DNA quality using a NanoDrop One Microvolume UV-Vis Spectrophotometer and quantified DNA using a Qubit 4 Fluorometer (ThermoFisher) with a Quant-iT dsDNA HS Assay Kit. We also measured DNA quality, quantity, and fragment length distributions using the Agilent TapeStation 4200 System with Genomic DNA ScreenTape and reagents before proceeding to library prepration. A typical preparation from a 20 mg spider yielded 8.5ug of DNA.

Illumina Sequencing

For Illumina sequencing, we extracted genomic DNA from a single, whole, unmated penultimate stage female to minimize the potential contribution of extraneous haplotypes from stored sperm after mating events. We submitted the HMW gDNA to the Johns Hopkins GCRF, where they prepared a PCR-free library of approximately 400 bp DNA insert size using the Illumina TruSeq PCR-Free High Throughput Library Prep Kit (San Diego, CA, USA), according to the manufacturer's protocol. They then sequenced the prepared library on an Illumina NovaSeq 6000 Sequencing System with 150bp paired-end chemistry.

ONT Sequencing

For ONT sequencing, we extracted HMW genomic DNA from 3 adult females. We prepared sequencing libraries using the Ligation Sequencing Kit (SQK-LSK109) (Oxford Nanopore Technologies, UK), according to the manufacturer's protocols. Third party reagents we used during library preparation included: New England Biolabs (New England Biolabs, Ipswitch, MA, USA) NEBNext End Repair/dA-Tailing Module (E7546), NEBNext FFPE DNA Repair Mix (M6630), and NEB Quick Ligation Module (E6056). We then sequenced the libraries, using ONT R.9.4.1 flowcells (FLO-PRO002) on an ONT PromethION sequencing platform. We then used ONT's Albacore basecalling software v.2.0.1 (RRID:SCR_015897) to basecall the raw fast5 data.

PacBio HiFi Sequencing

For PacBio sequencing, HMW DNA was extracted from a single adult female spider provided to Circulomics (Baltimore, MD, USA). They extracted DNA using a modified protocol with the Nanobind Tissue Kit (Circulomics, #NB-900-701-01). Briefly, they froze and crushed a single, adult female spider with a pellet pestle (Fisher, #12-141-364) in a Protein LoBind tube (Eppendorf, #022431081) containing 200 uL of Buffer CT. The crushed spider was centrifuged at 16,000 x g at 4 C for 2 min. The supernatant was discarded, and the pellet was resuspended in 500 ul Buffer CT and the mixture was transferred to a 2.0 mL Protein LoBind tube (Eppendorf # 022431102). The suspension was spun again at 16,000 x g at 4 C for 2 min and the supernatant discarded. The spider tissue pellet was combined with 20 ul Proteinase K and 150 ul Buffer PL1 and resuspended by pipetting with a P200 wide bore pipette tip. The tissue was incubated on a ThermoMixer at 55 C with 900 rpm mixing for 1 hour. After lysis, 20 ul RNaseA was added, and the lysate was mixed by pipetting with a P200 wide bore pipette tip. The lysate was incubated at RT for 3 min. After RNaseA incubation, 25 ul Buffer SB was added, the lysate was vortexed 5 x 1 sec pulses, and then centrifuged at 16,000 x g at 4 C for 5 min. The supernatant (~200 ul) was transferred to a 70uM filter (Fisher # NC1444112) set in a new 1.5 mL Protein LoBind tube (Eppendorf # 022431081). The tube with the 70 uM filter was spun on a mini-centrifuge (Ohaus # FC5306) for 1 sec and then the filter was dicarded. 50 ul Buffer BL3 was added to the cleared lysate and the tube was inversion mixed 10X. The tube was then incubated on a ThermoMixer at 55 C with 900 rpm mixing for 5 min. After incubation, the tube was allowed to come to RT, which took about 2 min. The tube was spun for 1 sec on a mini-centrifuge to spin down condensate from the lid. One 5 mm Nanobind disk was added to the tube followed by 250 uL isopropanol and then the tube was inversion mixed 5X. The tube was then rocked on a platform rocker (ThermoScientific # M48725Q) at RT and max speed for 30 min. The DNA-bound Nanobind disk was washed according to handbook directions with one 500 ul CW1 wash and one 500 ul CW2 wash. The tube with the disk was tap spun for 2 x 1 sec to dry the disk. The DNA was eluted with 50 ul Buffer EB and incubated at RT overnight. The next day, the eluate was pipette mixed with

a standard bore pipette tip 5x and then quantitated with Nanodrop and Qubit dsDNA BR assay and then sized by pulsed-field gel electrophoresis.

We then submitted the DNA sample to the University of Maryland School of Medicine Genomics Core Facility for PacBio HiFi sequencing. There, they size selected the DNA using a Safe Science BluePippin with a 9kb high-pass cutoff. They prepared the sequencing library using the Express v2 kit, according to the standard protocol for preparing HiFi sequencing libraries. They then sequenced the library on a PacBio Sequel II 8M SMRT Cell using a 30 hour HiFi run mode and processed using SMRT Link v.9.0 software.

Dovetail Chicago and Dovetail Hi-C Sequencing

To further improve the *U. diversus* genome assembly, we used proximity ligation-based sequencing techniques to scaffold intermediate versions of our assembly. We provided 19 spider specimens to Dovetail Genomics (Scotts Valley, CA, USA) for Chicago and Hi-C library preparation as previously described[54]. They prepared a Chicago library using 15 pooled adult females and a Hi-C library using 4 pooled adult females. They sequenced both the prepared Chicago and Dovetail Hi-C libraries on an Illumina HiSeq X sequencing platform on 1 flowcell.

DNA-seq and RNA-seq QA/QC

For Illumina, we examined read quality using FastQC[130] v.0.11.9 (RRID:SCR_014583). For DNA-seq data, we determined that, due to high quality of reads and absence of adapter

sequences, no further processing would be required and proceeded to assembly with raw read data.For RNA-seq data, we used TrimGalore[131] v.0.4.2 (RRID:SCR_011847) to apply quality filtering and remove adapter sequences from the FASTQ files. We performed additional filtering for quality with Trimmomatic[132] v.0.33 (RRID:SCR_011848) . For ONT, reads shorter than 3 kbp were discarded. The length-filtered ONT long reads were used in downstream assembly.

Genome Size, Heterozygosity, and Unique Sequence Estimation

Prior to assembly, we used Jellyfish[46] v.2.2.4 (RRID:SCR_005491) to count the frequency of canonical 21-mers in our Illumina sequencing data. We used the resulting sorted *k*-mer frequencies vs counts histogram as input to GenomeScope[47,133] v.2.0 to estimate genome size, heterozygosity, and repetitiveness.

Recovery of Mitogenome

We used Novoplasty[69] v.4.2 (RRID:SCR_017335) to generate a complete circularized mitochondrial sequence using raw Illumina read data. The mitochondrial sequences of several spider species were used to provide seed sequences (**Supplemental Table S3**). The resulting mitogenome sequences assembled by Novoplasty were compared for consensus. The consensus mitogenome was uploaded to the MITOS 2 web server[84] for annotation. The CGView web server[134] (RRID:SCR_011779) was used to visualize the annotated mitogenome.

Nuclear Genome Assembly

De novo Nuclear Genome Assembly with MaSuRCA

Illumina reads were assembled into contigs and the resulting contigs were scaffolded with ONT long reads using the MaSuRCA assembly pipeline[49,50] v.3.4.2 (RRI:010691). We used default settings, including the default CABOG contigging module in lieu of the Flye assembler. The resulting genome assembly is referred to as *U. diversus* v.1.0.

To improve the assembly, we used *Rascaf*[51] v.2016-11-29 to scaffold with Illumina RNA-seq read data. The resulting genome assembly is referred to as *U. diversus* v.1.1. To reduce redundancy in the assembly due to the presence of alternative haplotigs, we used Pseudohaploid with default settings. The resulting genome assembly is referred to as *U. diversus* v.1.2

De novo Nuclear Genome Assembly with PB-IPA

We used PacBio's Improved Phased Assembly (IPA) HiFi Genome Assembler with default settings, specifying a genome size of 1.9 Gbp, to assemble the HiFi reads. The resulting genome assembly is referred to as *U. diversus* v.2.0.

Merging MaSuRCA and PB-IPA Assemblies with SAMBA

We used the SAMBA tool distributed with MaSuRCA to merge the MaSuRCA assembly, *U. diversus* v.1.2, and the PB-IPA assembly, *U. diversus* v.2.0. The resulting genome assembly is referred to as *U. diversus* v.3.0.

Scaffolding Assemblies with *HiRise*

The initial *U. diversus* v.1.2 draft assembly obtained using a combination of MaSuRCA, Rascaf, and Pseudohaploid was provided to Dovetail Genomics in FASTA format. The resulting genome assembly is referred to as *U. diversus* v.1.3.

The merged MaSuRCA and PB-IPA assembly, *U. diversus* v.3.0, was provided to Dovetail Genomics in FASTA format. The resulting genome assembly is referred to as *U. diversus* v.3.1.

Genome Assembly Metrics and Assessments

For each assembly, completeness was estimated with Benchmarking Universal Single-Copy Orthologs (BUSCO)[135–137] v.5.2.1 (RRID:SCR_015008) using the arachnida_odb10 database[138]. Contiguity of each assembly was evaluated for comparison using Quast[139] v.5.0.2 (RRID:SCR_001228).

Genome-Guided Transcriptome Assembly

Cleaned and trimmed Illumina RNA-seq reads were aligned to the genome using *HISAT2*[140] v.2.2.1. We then used the Trinity assembler[43] v.2.12.0 to produce a genome-guided transcriptome assembly [--CPU 60 –max_memory 200G –genome_guided_max_intron 20000 –SS_lib_type RF –include_supertranscripts –verbose]. We used TransDecoder[57] v.5.5.0 with default settings, including homology searches using both BlastP[58,59] against a SwissProt UniProt database[141] as well as the Pfam database[60] v.32, as ORF retention criteria.

Repeat Annotations

To characterize the repeat elements in the *U. diversus* genome, we generated a custom *de novo* repeat library using RepeatModeler[55] v.2.0.2 with default parameters. We used RepeatMasker[142] v.4.1.2 to screen and mask repeat and low-complexity regions of the genome with the Dfam consensus[143] v.3.4 and RepBase RepeatMasker Edition[56] v.2018-10-26 repeat libraries.

Annotation of Protein Coding Genes

We performed gene annotation using the BRAKER 2 pipeline[61,62,144–154] v.2.1.6 with RNA-seq evidence and protein homology evidence based on a custom library of spider sequences obtained from NCBI. BRAKER2 uses RNA-seq data to produce intron hints for training the *ab initio* gene prediction program AUGUSTUS[146,147] on a species-specific model. This species-specific model is then used in conjunction with RNA-seq data to predict protein coding genes.

The bam file previously generated in transcriptome assembly and analysis was passed to BRAKER2, which was run with default settings.

Annotation of Non-Coding RNAs

We used tRNAscan-SE[65,66] v.2.0.7 with default settings to predict tRNAs. We then used Barrnap[67] v.0.9 (RRID:SCR_015995) with default settings to predict rRNAs.

Functional Annotation

We started the annotation of predicted genes used the BLAST+ blastp algorithm. First, we obtained the longest coding sequence for each gene predicted by BRAKER2. We then used the EMBOSS[155] v.6.6.0.0 Transeq tool to translate and trim the coding sequences. Once translated and trimmed, we used the BLAST+ v.2.10.1+ Blastp tool to search against the UniProt SwissProt database with an e-value cutoff of 1e-10. We used InterProScan[63,64] (RRID:SCR_005829) to predict motifs, domains, and gene ontology (GO)[156,157] terms (RRID:SCR_002811), as well as MetaCyc[158,159] and Reactome[160,161] pathways,using the following analyses: CDD[162] v.3.18, Coils v.2.2.1, Gene3D[163] v.4.3.0, Hamap[164] v.2020-05, MobiDBLite[165] v.2.0, PANTHER[166] v.15.0, Pfam[60] v.34.0, PIRSF[167] v.3.10, PIRSR[168] v.2021-02, PRINTS[169] v.42.0, ProSitePatterns[170,171] v.2021-01, ProSiteProfiles[170,171] v.2021-01, SFLD[172] v.4, SMART[173,174] v.7.1, SUPERFAMILY[175,176] v.1.75, and TIGRFAM[177–180] v.15.0.

Spidroins

Identification of Spidroin Candidate Sequences

We identified *Uloborus diversus* by conducting BLAST[58,59] searches using the list of spidroin sequences included in **Supplemental Table S5** as queries against the assembled genome, transcriptome, and gene models predicted by BRAKER2. We looked for matches to both N- and C-terminal sequences from members of each type of spidroin, as well as to available repetitive motifs. After cross-referencing genomic coordinates with gene models and transcripts, we used JBrowse[181] to visualize mapping of Illumina RNAseq data and PacBio HiFi reads to the assembled genome. RNAseq reads were mapped to the genome with HISAT2[140], while minimap2[182,183] was used to map PacBio HiFi reads. Samtools[149] was used to convert the resulting SAM files to BAM files, as well as to sort and index the BAM files. For each spidroin candidate, the entire sequence from start codon to stop codon, ignoring any predicted splicing, with an additional 5 kb of sequence on both the 5' and 3' end, was translated in all six frames using the ExPASy Translate Tool via the ExPASy web server[184] and inspected for ORFs as well as the presence of repetitive motifs characteristic of spidroins. Predicted splice sites were compared with RNAseq data. Unsupported splice sites, either by lack of evidence in the mapping of RNAseq reads or by the obvious presence of spidroin repeat motifs within the predicted intronic region, were removed from the annotations. Spidroins sequences were called based upon the preponderance of available evidence, which in some cases conflicted with the structure predicted by BRAKER2.

Spidroin Sequence Analysis

We used the ExPASy web server tool ProtScale to find the amino acid composition of each sequence, as well as to estimate the hydrophobicity using the the Kyte-Doolittle method[184,185]. We used the PSIPRED v.4.0 tool in the UCL Bioinformatics Group's PSIPRED Protein Analysis Workbench[186] to predict the secondary structure of each sequence. The sequences were often too long and necessitated judicious segmentation into reasonable sequences that were short enough for analysis. In such cases, we selected natural breaks in the sequence structure, such as separating the N-terminal region from the repetitive regions, etc. We used SignalP[187] v.6.0 to predict the presence signal peptides and signal peptidase cleavage sites in the N-terminal regions.

Data Availability

The raw sequencing data and assembled genome presented in this study have been submitted to the NCBI BioProject database (https://www.ncbi.nlm.nih.gov/bioproject/) under accession number PRNA846873.

Conflicts of Interest

The authors declare no conflicts of interest.

Funding

J.M. acknowledges funding from the NSF Graduate Research Fellowship Program (DGE-1746891). A.G. acknowledges funding from NIH (R35GM124883). A.V.Z. acknowledges funding from the USDA National Institute of Food and Agriculture (2018-67015-28199), NSF (IOS-1744309), and NIH (R01-HG006677 and R35-GM130151).

Author Contributions

J.M., A.Z. and A.G. designed the research study. J.M. performed DNA purification and sample preparation for Illumina and Oxford Nanopore sequencing. J.M. performed all computational analyses, except for HiRise scaffolding (performed by Dovetail), MaSuRCA and SAMBA. A.Z. performed MaSuRCA assembly and merging with SAMBA. J.M. and A.G. analyzed the data and wrote the paper.

Acknowledgements

We thank Circulomics Inc., particularly Kelvin Liu and Michelle Kim, for assistance in DNA extraction for PacBio HiFi sequencing. We thank the Johns Hopkins University Genomics Core, and David Mohr in particular, for Illumina sequencing and consultation. We additionally thank the University of Maryland Genomic Resource Center, and Luke Tallon specifically, for PacBio HiFi sequencing. We thank Dovetail Genomics, particularly Mark Daly and Tom Swale, for Chicago

and Dovetail Hi-C library preparation and sequencing, as well as HiRise assembly scaffolding and consultation. We thank the members of the Timp Lab, in particular Winston Timp, Norah Sadowski, and Rachael Workman, for training and graciously permitting the use of their ONT PromethION and TapeStation. We thank Gordus lab members, James Taylor, Michael Schatz, Bob Johnston, Rajiv McCoy, Prashant Sharma, and Ben Matthews for helpful discussions and comments on the manuscript.

Figure Legends

Figure 1: Spider Phylogeny

A) A female *U. diversus.*

B) Phylogeny of spiders. Orb weaver families are highlighted in orange. Species with sequenced genomes are highlighted in blue. *U. diversus* is highlighted in red. Example webs from Rooney[188], Glatz[189], Coddington[18]. UDOH = Uloboridae, Deinopidae, Oecobiidae, Hersiliidae. RTA = Retrolateral tibial apophysis clade. O = Ordovician, S = Silurian, D = Devonian, C = Carboniferous, P = Permian, T = Triassic, J = Jurassic, K = Cretaceous, Pg. = Paleogene, Ng. = Neogene, Mya = millions of years ago

Figure 2: GenomeScope Plot from Illumina Data

A) Kmer spectra for Illumina reads from a single, virgin female. The diploid and haploid peaks are at 70x and 35x coverage, respectively.

Figure 3: Chromosome Scale Genome Assembly

A) Karyotype of female and male embryos. Female and male diploid sizes of 20 and 18, respectively, indicate a ♂X1X2/♀X1X1X2X2 sex-determination system, with 8 autosomes.

B) Hi-C linkage map of assembled scaffolds. The 10 largest scaffolds are annotated.

C) Comparison of HiRise and MaSuRCA assemblies. The majority of the HiRise assembly is captured by the first 10 scaffolds.

D) Circos plot of 10 largest nuclear scaffolds, highlighting GC content, repeat content, and gene content across the scaffolds.

E) Circos plot of mitochondrial scaffold, highlighting tRNA-coding loci, protein-coding loci, and GC content.

Figure 4: Gene Annotations

A) Gene loci for spidroins and *hox* gene clusters.

B) Domain composition of identified spidoins. (Repeat region annotations are condensed for clarity.)

Figure 5: Synteny and Chromosomal Rearrangements

A) Inter-anchor mRNA ID difference distribution of syntenic blocks identified by AnchorWave analysis. Each syntenic block is defined by ORF or inter-ORF anchors. All ORFS are numerically annotated in consecutive order from scaffold 1 through scaffold 10. Inter-anchor mRNA ID difference is defined as the difference in these numerical ORF IDs between consecutive ORF anchors. If the distance equals 1, it means the two anchors are consecutive ORFs within the block. Asterisk indicates syntenic blocks used in **E**.

B) Inter-anchor Mbp difference distribution of syntenic blocks identified with AnchorWave analysis. Inter-anchor difference was calculated as the base-pair distance between consecutive ORF anchors within a syntenic block. Asterisk indicates syntenic blocks used in **E**.

C) Ribbon plot of all AnchorWave-defined syntenic blocks shared between chromosomal scaffolds.

D) Ribbon plot of filtered AnchorWave-defined syntenic blocks shared between chromsomal scaffolds. Only blocks consisting of consecutive ORF anchors < 4 mRNA IDs apart are plotted.

E) Ribbon plot of filtered AnchorWave-defined syntenic blocks shared between *U. diversus* and *A. bruennichi, M. bourneti,* and *D. plantarius* chromsomal scaffolds. Only blocks consisting of consecutive ORF anchors < 4 mRNA IDs apart are plotted.

Figure 6: Sex Chromosomes

A) Read depth of Illumina reads from a male spider aligned to the chromosomal scaffolds. Scaffolds 3 and 10 (asterisks) exhibited lower read depth than other scaffolds.

B) Venn diagram of shared sex-associated genes identified in *U. diversus*, *S. mimosarum*, and *A. bruennichi*.

C) Ribbon plot of shared synteny between predicted X chromosomes from *U. diversus* and *A. bruennichi*.

Table 1 - Summary of Library Statistics.

Table 2 - Summary of Genome Assembly Statistics.

Table 3 - Summary of Genome Assembly BUSCO Scores.

Table 4 - Summary of Repeat Content.

Table 5 - Summary of Annotation Statistics.

Table 6 - Summary of InterproScan Results.

Table 7 - Summary of Spidroin Gene Features.

Table S1 - Comparison of Genome Statistics for Published Genomes.

Table S2 - Summary of Spider Genome Repeat Content.

Table S3 – Library of Annotated Genes from Spider Genomes.

Table S4 - Mitogenome Sequences Used for NOVOplasty Seeds.

Table S5 - Spidroin Protein Sequences Used in BLAST Searches.

Table S6 - Summary of Common Sex-Linked Annotations.

Literature Cited

1. Gloor D, Nentwig W, Blick T, Kropf C. World Spider Catalog. Natural History Museum Bern; 2017; doi: 10.24436/2.

2. Vollrath F, Selden P. The Role of Behavior in the Evolution of Spiders, Silks, and Webs. *Annu Rev Ecol Evol Syst*. 2007; doi: 10.1146/annurev.ecolsys.37.091305.110221.

3. Blackledge TA, Scharff N, Coddington JA, Szüts T, Wenzel JW, Hayashi CY, et al.. Reconstructing web evolution and spider diversification in the molecular era. *Proc Natl Acad Sci USA*. 2009; doi: 10.1073/pnas.0901377106.

4. Gatesy J, Hayashi C, Motriuk D, Woods J, Lewis R. Extreme Diversity, Conservation, and Convergence of Spider Silk Fibroin Sequences. *Science*. 2001; doi: 10.1126/science.1057561.

5. Foelix RF. Biology of spiders. 3rd ed. Oxford ; New York: Oxford University Press;

6. Vollrath F. Biology of spider silk. *International Journal of Biological Macromolecules*. 1999; doi: 10.1016/S0141-8130(98)00076-2.

7. Fernández R, Kallal RJ, Dimitrov D, Ballesteros JA, Arnedo MA, Giribet G, et al.. Phylogenomics, Diversification Dynamics, and Comparative Transcriptomics across the Spider Tree of Life. *Current Biology*. 2018; doi: 10.1016/j.cub.2018.03.064.

8. Coddington JA, Agnarsson I, Hamilton CA, Bond JE. Spiders did not repeatedly gain, but repeatedly lost, foraging webs. *PeerJ*. 2019; doi: 10.7717/peerj.6703.

9. Kallal RJ, Kulkarni SS, Dimitrov D, Benavides LR, Arnedo MA, Giribet G, et al.. Converging on the orb: denser taxon sampling elucidates spider phylogeny and new analytical methods support repeated evolution of the orb web. *Cladistics*. 2021; doi: 10.1111/cla.12439.

10. Sahni V, Blackledge TA, Dhinojwala A. Viscoelastic solids explain spider web stickiness. *Nat Commun*. 2010; doi: 10.1038/ncomms1019.

11. Opell BD, Hendricks ML. The role of granules within viscous capture threads of orb-weaving spiders. *Journal of Experimental Biology*. 2010; doi: 10.1242/jeb.036947.

12. Hayashi CY, Lewis RV. Molecular Architecture and Evolution of a Modular Spider Silk Protein Gene. *Science*. 2000; doi: 10.1126/science.287.5457.1477.

13. Hayashi CY, Lewis RV. Evidence from flagelliform silk cDNA for the structural basis of elasticity and modular nature of spider silks 1 1Edited by M. F. Moody. *Journal of Molecular Biology*. 1998; doi: 10.1006/jmbi.1997.1478.

14. Peters HM. On the spinning apparatus and the structure of the capture threads ofDeinopis subrufus (Araneae, Deinopidae). *Zoomorphology*. 1992; doi: 10.1007/BF01632992.

15. Peters HM. The spinning apparatus of Uloboridae in relation to the structure and construction of capture threads (Arachnida, Araneida). *Zoomorphology*. 1984; doi: 10.1007/BF00312023.

16. Blackledge TA, Hayashi CY. Unraveling the mechanical properties of composite silk threads spun by cribellate orb-weaving spiders. *Journal of Experimental Biology*. 2006; doi: 10.1242/jeb.02327.

17. Piorkowski D, Blackledge TA, Liao C-P, Joel A-C, Weissbach M, Wu C-L, et al.. Uncoiling springs promote mechanical functionality of spider cribellate silk. *Journal of Experimental Biology*. 2020; doi: 10.1242/jeb.215269.

18. Coddington JA. ORB WEBS IN “NON-ORB WEAVING” OGRE-FACED SPIDERS (ARANEAE: DINOPIDAE): A QUESTION OF GENEALOGY. *Cladistics*. 1986; doi: 10.1111/j.1096-0031.1986.tb00442.x.

19. Zschokke S, Vollrath F. Web construction patterns in a range of orb weaving spiders (Araneae). *European Journal of Entomology*. 92:523–411995;

20. Witt PN, Reed CF. Spider-Web Building: Measurement of web geometry identifies components in a complex invertebrate behavior pattern. *Science*. 1965; doi: 10.1126/science.149.3689.1190.

21. Hesselberg T, Vollrath F. The effects of neurotoxins on web-geometry and web-building behaviour in Araneus diadematus Cl. *Physiology & Behavior*. 2004; doi: 10.1016/j.physbeh.2004.04.058.

22. Reed CF, Witt PN, Scarboro MB. Maturation andd-amphetamine-induced changes in web building. *Dev Psychobiol*. 1982; doi: 10.1002/dev.420150109.

23. Corver A, Wilkerson N, Miller J, Gordus A. Distinct movement patterns generate stages of spider web building. *Current Biology*. 2021; doi: 10.1016/j.cub.2021.09.030.

24. Sanggaard KW, Bechsgaard JS, Fang X, Duan J, Dyrlund TF, Gupta V, et al.. Spider genomes provide insight into composition and evolution of venom and silk. *Nat Commun*. 2014; doi: 10.1038/ncomms4765.

25. Stellwagen SD, Renberg RL. Toward Spider Glue: Long Read Scaffolding for Extreme Length and Repetitious Silk Family Genes AgSp1 and AgSp2 with Insights into Functional Adaptation. *G3 Genes|Genomes|Genetics*. 2019; doi: 10.1534/g3.119.400065.

26. Ayoub NA, Garb JE, Kuelbs A, Hayashi CY. Ancient Properties of Spider Silks Revealed by the Complete Gene Sequence of the Prey-Wrapping Silk Protein (AcSp1). *Molecular Biology and Evolution*. 2013; doi: 10.1093/molbev/mss254.

27. Babb PL, Lahens NF, Correa-Garhwal SM, Nicholson DN, Kim EJ, Hogenesch JB, et al.. The Nephila clavipes genome highlights the diversity of spider silk genes and their complex expression. *Nat Genet*. 2017; doi: 10.1038/ng.3852.

28. Kono N, Nakamura H, Ohtoshi R, Moran DAP, Shinohara A, Yoshida Y, et al.. Orb-weaving spider Araneus ventricosus genome elucidates the spidroin gene catalogue. *Sci Rep*. 2019; doi: 10.1038/s41598-019-44775-2.

29. Sheffer MM, Hoppe A, Krehenwinkel H, Uhl G, Kuss AW, Jensen L, et al.. Chromosome-level reference genome of the European wasp spider *Argiope bruennichi* : a resource for studies on range expansion and evolutionary adaptation. *GigaScience*. 2021; doi: 10.1093/gigascience/giaa148.

30. Sánchez-Herrero JF, Frías-López C, Escuer P, Hinojosa-Alvarez S, Arnedo MA, Sánchez-Gracia A, et al.. The draft genome sequence of the spider Dysdera silvatica (Araneae, Dysderidae): A valuable resource for functional and evolutionary genomic studies in chelicerates. *GigaScience*. 2019; doi: 10.1093/gigascience/giz099.

31. Schwager EE, Sharma PP, Clarke T, Leite DJ, Wierschin T, Pechmann M, et al.. The house spider genome reveals an ancient whole-genome duplication during arachnid evolution. *BMC Biol*. 2017; doi: 10.1186/s12915-017-0399-x.

32. Fan Z, Yuan T, Liu P, Wang L-Y, Jin J-F, Zhang F, et al.. A chromosome-level genome of the spider *Trichonephila antipodiana* reveals the genetic basis of its polyphagy and evidence of

an ancient whole-genome duplication event. *GigaScience*. 2021; doi: 10.1093/gigascience/giab016.

33. Yu N, Li J, Liu M, Huang L, Bao H, Yang Z, et al.. Genome sequencing and neurotoxin diversity of a wandering spider *Pardosa pseudoannulata* (pond wolf spider). Genomics; 2019 Aug.

34. Liu S, Aagaard A, Bechsgaard J, Bilde T. DNA Methylation Patterns in the Social Spider, Stegodyphus dumicola. *Genes*. 2019; doi: 10.3390/genes10020137.

35. Escuer P, Pisarenco VA, Fernández-Ruiz AA, Vizueta J, Sánchez-Herrero JF, Arnedo MA, et al.. The chromosome-scale assembly of the Canary Islands endemic spider *Dysdera silvatica* (Arachnida, Araneae) sheds light on the origin and genome structure of chemoreceptor gene families in chelicerates. *Molecular Ecology Resources*. 2022; doi: 10.1111/1755-0998.13471.

36. Cerca J, Armstrong EE, Vizueta J, Fernández R, Dimitrov D, Petersen B, et al.. The *Tetragnatha kauaiensis* Genome Sheds Light on the Origins of Genomic Novelty in Spiders. Gossmann T, editor. *Genome Biology and Evolution*. 2021; doi: 10.1093/gbe/evab262.

37. Zhu B, Jin P, Hou Z, Li J, Wei S, Li S. Chromosomal-level genome of a sheet-web spider provides insight into the composition and evolution of venom. *Molecular Ecology Resources*. 2022; doi: 10.1111/1755-0998.13601.

38. Hendrickx F, De Corte Z, Sonet G, Van Belleghem SM, Köstlbacher S, Vangestel C. A masculinizing supergene underlies an exaggerated male reproductive morph in a spider. *Nat Ecol Evol*. 2022; doi: 10.1038/s41559-021-01626-6.

39. Kono N, Nakamura H, Mori M, Yoshida Y, Ohtoshi R, Malay AD, et al.. Multicomponent nature underlies the extraordinary mechanical properties of spider dragline silk. *Proc Natl Acad Sci USA*. 2021; doi: 10.1073/pnas.2107065118.

40. Purcell J, Pruitt JN. Are personalities genetically determined? Inferences from subsocial spiders. *BMC Genomics*. 2019; doi: 10.1186/s12864-019-6172-5.

41. Li Y-Y, Tsai J-M, Wu C-Y, Chiu Y-F, Li H-Y, Warrit N, et al.. In Silico Assessment of Probe-Capturing Strategies and Effectiveness in the Spider Sub-Lineage Araneoidea (Order: Araneae). *Diversity*. 2022; doi: 10.3390/d14030184.

42. Kono N, Ohtoshi R, Malay AD, Mori M, Masunaga H, Yoshida Y, et al.. Darwin's bark spider shares a spidroin repertoire with *Caerostris extrusa* but achieves extraordinary silk toughness through gene expression. *Open Biol*. 2021; doi: 10.1098/rsob.210242.

43. Garb JE, Sharma PP, Ayoub NA. Recent progress and prospects for advancing arachnid genomics. *Current Opinion in Insect Science*. 2018; doi: 10.1016/j.cois.2017.11.005.

44. Eberhard WG. The ecology of the web ofUloborus diversus (Araneae: Uloboridae). *Oecologia*. 1971; doi: 10.1007/BF00389107.

45. Giani AM, Gallo GR, Gianfranceschi L, Formenti G. Long walk to genomics: History and current approaches to genome sequencing and assembly. *Computational and Structural Biotechnology Journal*. 2020; doi: 10.1016/j.csbj.2019.11.002.

46. Marçais G, Kingsford C. A fast, lock-free approach for efficient parallel counting of occurrences of k-mers. *Bioinformatics*. 2011; doi: 10.1093/bioinformatics/btr011.

47. Ranallo-Benavidez TR, Jaron KS, Schatz MC. GenomeScope 2.0 and Smudgeplot for reference-free profiling of polyploid genomes. *Nat Commun*. 2020; doi: 10.1038/s41467-020-14998-3.

48. Sember A, Pappová M, Forman M, Nguyen P, Marec F, Dalíková M, et al.. Patterns of Sex Chromosome Differentiation in Spiders: Insights from Comparative Genomic Hybridisation. *Genes*. 2020; doi: 10.3390/genes11080849.

49. Zimin AV, Marçais G, Puiu D, Roberts M, Salzberg SL, Yorke JA. The MaSuRCA genome assembler. *Bioinformatics*. 2013; doi: 10.1093/bioinformatics/btt476.

50. Zimin AV, Puiu D, Luo M-C, Zhu T, Koren S, Marçais G, et al.. Hybrid assembly of the large and highly repetitive genome of *Aegilops tauschii* , a progenitor of bread wheat, with the MaSuRCA mega-reads algorithm. *Genome Res*. 2017; doi: 10.1101/gr.213405.116.

51. Song L, Shankar DS, Florea L. Rascaf: Improving Genome Assembly with RNA Sequencing Data. *Plant Genome*. 2016; doi: 10.3835/plantgenome2016.03.0027.

52. Chen L-Y, VanBuren R, Paris M, Zhou H, Zhang X, Wai CM, et al.. The bracteatus pineapple genome and domestication of clonally propagated crops. *Nat Genet*. 2019; doi: 10.1038/s41588-019-0506-8.

53. Zimin AV, Salzberg SL. The SAMBA tool uses long reads to improve the contiguity of genome assemblies. Shao M, editor. *PLoS Comput Biol*. 2022; doi: 10.1371/journal.pcbi.1009860.

54. Putnam NH, O’Connell BL, Stites JC, Rice BJ, Blanchette M, Calef R, et al.. Chromosome-scale shotgun assembly using an in vitro method for long-range linkage. *Genome Res*. 2016; doi: 10.1101/gr.193474.115.

55. Flynn JM, Hubley R, Goubert C, Rosen J, Clark AG, Feschotte C, et al.. RepeatModeler2 for automated genomic discovery of transposable element families. *Proc Natl Acad Sci USA*. 2020; doi: 10.1073/pnas.1921046117.

56. Bao W, Kojima KK, Kohany O. Repbase Update, a database of repetitive elements in eukaryotic genomes. *Mobile DNA*. 2015; doi: 10.1186/s13100-015-0041-9.

57. Haas BJ, Papanicolaou A, Yassour M, Grabherr M, Blood PD, Bowden J, et al.. De novo transcript sequence reconstruction from RNA-seq using the Trinity platform for reference generation and analysis. *Nat Protoc*. 2013; doi: 10.1038/nprot.2013.084.

58. Altschul SF, Gish W, Miller W, Myers EW, Lipman DJ. Basic local alignment search tool. *Journal of Molecular Biology*. 1990; doi: 10.1016/S0022-2836(05)80360-2.

59. Altschul S. Gapped BLAST and PSI-BLAST: a new generation of protein database search programs. *Nucleic Acids Research*. 1997; doi: 10.1093/nar/25.17.3389.

60. Mistry J, Chuguransky S, Williams L, Qureshi M, Salazar GA, Sonnhammer ELL, et al.. Pfam: The protein families database in 2021. *Nucleic Acids Research*. 2021; doi: 10.1093/nar/gkaa913.

61. Hoff KJ, Lange S, Lomsadze A, Borodovsky M, Stanke M. BRAKER1: Unsupervised RNA-Seq-Based Genome Annotation with GeneMark-ET and AUGUSTUS: Table 1. *Bioinformatics*. 2016; doi: 10.1093/bioinformatics/btv661.

62. Brůna T, Hoff KJ, Lomsadze A, Stanke M, Borodovsky M. BRAKER2: automatic eukaryotic genome annotation with GeneMark-EP+ and AUGUSTUS supported by a protein database. *NAR Genomics and Bioinformatics*. 2021; doi: 10.1093/nargab/lqaa108.

63. Quevillon E, Silventoinen V, Pillai S, Harte N, Mulder N, Apweiler R, et al.. InterProScan: protein domains identifier. *Nucleic Acids Research*. 2005; doi: 10.1093/nar/gki442.

64. Jones P, Binns D, Chang H-Y, Fraser M, Li W, McAnulla C, et al.. InterProScan 5: genome-scale protein function classification. *Bioinformatics*. 2014; doi: 10.1093/bioinformatics/btu031.

65. Chan PP, Lowe TM. tRNAscan-SE: Searching for tRNA Genes in Genomic Sequences. In: Kollmar M, editor. *Gene Prediction*. New York, NY: Springer New York;

66. Chan PP, Lin BY, Mak AJ, Lowe TM. tRNAscan-SE 2.0: improved detection and functional classification of transfer RNA genes. *Nucleic Acids Research*. 2021; doi: 10.1093/nar/gkab688.

67. Seeman T. Barrnap: BAsic Rapbid Ribosomal RNA Predictor.

68. Boore JL. Animal mitochondrial genomes. *Nucleic Acids Research*. 1999; doi: 10.1093/nar/27.8.1767.

69. Dierckxsens N, Mardulyn P, Smits G. NOVOPlasty: *de novo* assembly of organelle genomes from whole genome data. *Nucleic Acids Res*. 2016; doi: 10.1093/nar/gkw955.

70. Wang Z-L, Wang Z-Y, Huang J, Yu X-P. The complete mitochondrial genome of an orb-weaver spider *Araneus angulatus* (Araneae: Araneidae). *Mitochondrial DNA Part B*. 2019; doi: 10.1080/23802359.2019.1687344.

71. Zhu X-L, Zhang Z-S. The complete mitochondrial genome of *Agelena silvatica* (Araneae: Agelenidae). *Mitochondrial DNA Part B*. 2017; doi: 10.1080/23802359.2017.1280702.

72. Wang Z-L, Li C, Fang W-Y, Yu X-P. The complete mitochondrial genome of orb-weaving spider *Araneus ventricosus* (Araneae: Araneidae). *Mitochondrial DNA*. 2014; doi: 10.3109/19401736.2014.971290.

73. Liu M, Zhang Z, Peng Z. The mitochondrial genome of the water spider *Argyroneta aquatica* (Araneae: Cybaeidae). *Zool Scr*. 2015; doi: 10.1111/zsc.12090.

74. Fang W-Y, Wang Z-L, Li C, Yang X-Q, Yu X-P. The complete mitogenome of a jumping spider Carrhotus xanthogramma (Araneae: Salticidae) and comparative analysis in four salticid mitogenomes. *Genetica*. 2016; doi: 10.1007/s10709-016-9936-8.

75. Masta SE, Boore JL. Parallel Evolution of Truncated Transfer RNA Genes in Arachnid Mitochondrial Genomes. *Molecular Biology and Evolution*. 2008; doi: 10.1093/molbev/msn051.

76. Kumar V, Tyagi K, Chakraborty R, Prasad P, Kundu S, Tyagi I, et al.. The Complete Mitochondrial Genome of endemic giant tarantula, Lyrognathus crotalus (Araneae: Theraphosidae) and comparative analysis. *Sci Rep*. 2020; doi: 10.1038/s41598-019-57065-8.

77. Li C, Wang Z-L, Fang W-Y, Yu X-P. The complete mitochondrial genome of the orb-weaving spider *Neoscona theisi* (Walckenaer) (Araneae: Araneidae). *Mitochondrial DNA Part A*. 2016; doi: 10.3109/19401736.2014.1003831.

78. Qiu Y, Song D, Zhou K, Sun H. The Mitochondrial Sequences of Heptathela hangzhouensis and Ornithoctonus huwena Reveal Unique Gene Arrangements and Atypical tRNAs. *J Mol Evol*. 2005; doi: 10.1007/s00239-004-0010-2.

79. Pan W-J, Fang H-Y, Zhang P, Pan H-C. The complete mitochondrial genome of striped lynx spider *Oxyopes sertatus* (Araneae: Oxyopidae). *Mitochondrial DNA*. 2014; doi: 10.3109/19401736.2014.958695.

80. Kim JY, Yoo JS, Park YC. The complete mitochondrial genome of the green crab spider *Oxytate striatipes* (Araneae: Thomisidae). *Mitochondrial DNA*. 2014; doi: 10.3109/19401736.2014.971268.

81. Pan W-J, Fang H-Y, Zhang P, Pan H-C. The complete mitochondrial genome of flat spider *Selenops bursarius* (Araneae: Selenopidae). *Mitochondrial DNA*. 2016; doi: 10.3109/19401736.2014.953105.

82. Tian X-X, Pan W-J, Chen L-L, Xu Y-Y, Pan H-C. The complete mitochondrial genome of stretch spider *Tetragnatha maxillosa* (Araneae: Tetragnathidae). *Mitochondrial DNA Part A*. 2016; doi: 10.3109/19401736.2015.1066352.

83. Wang Z-L, Li C, Fang W-Y, Yu X-P. The complete mitochondrial genome of the wolf spider *Wadicosa fidelis* (Araneae: Lycosidae). *Mitochondrial DNA Part A*. 2016; doi: 10.3109/19401736.2014.987260.

84. Bernt M, Donath A, Jühling F, Externbrink F, Florentz C, Fritzsch G, et al.. MITOS: Improved de novo metazoan mitochondrial genome annotation. *Molecular Phylogenetics and Evolution*. 2013; doi: 10.1016/j.ympev.2012.08.023.

85. Wang Z-L, Li C, Fang W-Y, Yu X-P. The Complete Mitochondrial Genome of two *Tetragnatha* Spiders (Araneae: Tetragnathidae): Severe Truncation of tRNAs and Novel Gene Rearrangements in Araneae. *Int J Biol Sci*. 2016; doi: 10.7150/ijbs.12358.

86. Pons J, Bover P, Bidegaray-Batista L, Arnedo MA. Arm-less mitochondrial tRNAs conserved for over 30 millions of years in spiders. *BMC Genomics*. 2019; doi: 10.1186/s12864-019-6026-1.

87. Kumari S, Lang G, DeSimone E, Spengler C, Trossmann VT, Lücker S, et al.. Engineered spider silk-based 2D and 3D materials prevent microbial infestation. *Materials Today*. 2020; doi: 10.1016/j.mattod.2020.06.009.

88. Xu M, Jiang Y, Pradhan S, Yadavalli VK. Use of Silk Proteins to Form Organic, Flexible, Degradable Biosensors for Metabolite Monitoring. *Front Mater*. 2019; doi: 10.3389/fmats.2019.00331.

89. Öksüz KE, Özkaya NK, İnan ZDŞ, Özer A. Novel natural spider silk embedded electrospun nanofiber mats for wound healing. *Materials Today Communications*. 2021; doi: 10.1016/j.mtcomm.2020.101942.

90. Choi D, Choy KL. Spider silk binder for Si-based anode in lithium-ion batteries. *Materials & Design*. 2020; doi: 10.1016/j.matdes.2020.108669.

91. Mayank, Bardenhagen A, Sethi V, Gudwani H. Spider-silk composite material for aerospace application. *Acta Astronautica*. 2022; doi: 10.1016/j.actaastro.2021.08.013.

92. Liu Z, Zhang M, Zhang Y, Xu Y, Zhang Y, Yang X, et al.. Spider silk-based tapered optical fiber for humidity sensing based on multimode interference. *Sensors and Actuators A: Physical*. 2020; doi: 10.1016/j.sna.2020.112179.

93. Lewis RV. Spider Silk: Ancient Ideas for New Biomaterials. *Chem Rev*. 2006; doi: 10.1021/cr010194g.

94. Teulé F, Furin WA, Cooper AR, Duncan JR, Lewis RV. Modifications of spider silk sequences in an attempt to control the mechanical properties of the synthetic fibers. *J Mater Sci*. 2007; doi: 10.1007/s10853-007-1642-6.

95. Kono N, Nakamura H, Mori M, Tomita M, Arakawa K. Spidroin profiling of cribellate spiders provides insight into the evolution of spider prey capture strategies. *Sci Rep*. 2020; doi: 10.1038/s41598-020-72888-6.

96. Garb JE, Hayashi CY. Modular evolution of egg case silk genes across orb-weaving spider superfamilies. *Proc Natl Acad Sci USA*. 2005; doi: 10.1073/pnas.0502473102.

97. Motriuk-Smith D, Smith A, Hayashi CY, Lewis RV. Analysis of the Conserved N-Terminal Domains in Major Ampullate Spider Silk Proteins. *Biomacromolecules*. 2005; doi: 10.1021/bm050472b.

98. Ayoub NA, Hayashi CY. Multiple Recombining Loci Encode MaSp1, the Primary Constituent of Dragline Silk, in Widow Spiders (Latrodectus: Theridiidae). *Molecular Biology and Evolution*. 2008; doi: 10.1093/molbev/msm246.

99. Liu FYC, Liu JYX, Yao X, Wang B. Hybrid sequencing reveals the full-length Nephila pilipes pyriform spidroin 1 (PySp1). *International Journal of Biological Macromolecules*. 2022; doi: 10.1016/j.ijbiomac.2021.12.078.

100. Wen R, Wang K, Meng Q. The three novel complete aciniform spidroin variants from Araneus ventricosus reveal diversity of gene sequences within specific spidroin type. *International Journal of Biological Macromolecules*. 2020; doi: 10.1016/j.ijbiomac.2020.04.142.

101. Wang K, Wen R, Jia Q, Liu X, Xiao J, Meng Q. Analysis of the Full-Length Pyriform Spidroin Gene Sequence. *Genes*. 2019; doi: 10.3390/genes10060425.

102. Wen R, Liu X, Meng Q. Characterization of full-length tubuliform spidroin gene from Araneus ventricosus. *International Journal of Biological Macromolecules*. 2017; doi: 10.1016/j.ijbiomac.2017.07.086.

103. Tremblay M-L, Xu L, Lefèvre T, Sarker M, Orrell KE, Leclerc J, et al.. Spider wrapping silk fibre architecture arising from its modular soluble protein precursor. *Sci Rep*. 2015; doi: 10.1038/srep11502.

104. Wen R, Wang K, Liu X, Li X, Mi J, Meng Q. Molecular cloning and analysis of the full-length aciniform spidroin gene from Araneus ventricosus. *International Journal of Biological Macromolecules*. 2018; doi: 10.1016/j.ijbiomac.2017.12.090.

105. Chaw R, Zhao Y, Wei J, Ayoub NA, Allen R, Atrushi K, et al.. Intragenic homogenization and multiple copies of prey-wrapping silk genes in Argiope garden spiders. *BMC Evol Biol*. 2014; doi: 10.1186/1471-2148-14-31.

106. Hayashi CY. Molecular and Mechanical Characterization of Aciniform Silk: Uniformity of Iterated Sequence Modules in a Novel Member of the Spider Silk Fibroin Gene Family. *Molecular Biology and Evolution*. 2004; doi: 10.1093/molbev/msh204.

107. Tarakanova A, Buehler MJ. The role of capture spiral silk properties in the diversification of orb webs. *J R Soc Interface*. 2012; doi: 10.1098/rsif.2012.0473.

108. Hawthorn AC, Opell BD. van der Waals and hygroscopic forces of adhesion generated by spider capture threads. *Journal of Experimental Biology*. 2003; doi: 10.1242/jeb.00618.

109. Correa-Garhwal SM, Chaw RC, Clarke TH, Alaniz LG, Chan FS, Alfaro RE, et al.. Silk genes and silk gene expression in the spider Tengella perfuga (Zoropsidae), including a potential cribellar spidroin (CrSp). Heneberg P, editor. *PLoS ONE*. 2018; doi: 10.1371/journal.pone.0203563.

110. Vienneau-Hathaway JM, Brassfield ER, Lane AK, Collin MA, Correa-Garhwal SM, Clarke TH, et al.. Duplication and concerted evolution of MiSp-encoding genes underlie the material properties of minor ampullate silks of cobweb weaving spiders. *BMC Evol Biol*. 2017; doi: 10.1186/s12862-017-0927-x.

111. Chen G, Liu X, Zhang Y, Lin S, Yang Z, Johansson J, et al.. Full-Length Minor Ampullate Spidroin Gene Sequence. Uversky VN, editor. *PLoS ONE*. 2012; doi: 10.1371/journal.pone.0052293.

112. Ohno S. Evolution by Gene Duplication. Berlin, Heidelberg: Springer Berlin / Heidelberg;

113. Sémon M, Wolfe KH. Consequences of genome duplication. *Current Opinion in Genetics & Development*. 2007; doi: 10.1016/j.gde.2007.09.007.

114. Zhang J. Evolution by gene duplication: an update. *Trends in Ecology & Evolution*. 2003; doi: 10.1016/S0169-5347(03)00033-8.

115. Clarke TH, Garb JE, Hayashi CY, Arensburger P, Ayoub NA. Spider Transcriptomes Identify Ancient Large-Scale Gene Duplication Event Potentially Important in Silk Gland Evolution. *Genome Biol Evol*. 2015; doi: 10.1093/gbe/evv110.

116. Clarke TH, Garb JE, Hayashi CY, Haney RA, Lancaster AK, Corbett S, et al.. Multi-tissue transcriptomics of the black widow spider reveals expansions, co-options, and functional processes of the silk gland gene toolkit. *BMC Genomics*. 2014; doi: 10.1186/1471-2164-15-365.

117. Garcia-Fernàndez J, Holland PWH. Archetypal organization of the amphioxus Hox gene cluster. *Nature*. 1994; doi: 10.1038/370563a0.

118. Pace RM, Grbić M, Nagy LM. Composition and genomic organization of arthropod Hox clusters. *EvoDevo*. 2016; doi: 10.1186/s13227-016-0048-4.

119. Song B, Marco-Sola S, Moreto M, Johnson L, Buckler ES, Stitzer MC. AnchorWave: Sensitive alignment of genomes with high sequence diversity, extensive structural polymorphism, and whole-genome duplication. *Proc Natl Acad Sci USA*. 2022; doi: 10.1073/pnas.2113075119.

120. Hakes L, Pinney JW, Lovell SC, Oliver SG, Robertson DL. All duplicates are not equal: the difference between small-scale and genome duplication. *Genome Biol*. 2007; doi: 10.1186/gb-2007-8-10-r209.

121. Waters PD, Patel HR, Ruiz-Herrera A, Álvarez-González L, Lister NC, Simakov O, et al.. Microchromosomes are building blocks of bird, reptile, and mammal chromosomes. *Proc Natl Acad Sci USA*. 2021; doi: 10.1073/pnas.2112494118.

122. White MJD. Modes of speciation. San Francisco: W. H. Freeman;

123. Král J, Forman M, Kořínková T, Lerma ACR, Haddad CR, Musilová J, et al.. Insights into the karyotype and genome evolution of haplogyne spiders indicate a polyploid origin of lineage with holokinetic chromosomes. *Sci Rep*. 2019; doi: 10.1038/s41598-019-39034-3.

124. Bechsgaard J, Schou MF, Vanthournout B, Hendrickx F, Knudsen B, Settepani V, et al.. Evidence for Faster X Chromosome Evolution in Spiders. Larracuente A, editor. *Molecular Biology and Evolution*. 2019; doi: 10.1093/molbev/msz074.

125. Malay AD, Arakawa K, Numata K. Analysis of repetitive amino acid motifs reveals the essential features of spider dragline silk proteins. Zou Q, editor. *PLoS ONE*. 2017; doi: 10.1371/journal.pone.0183397.

126. Rising A, Nimmervoll H, Grip S, Fernandez-Arias A, Storckenfeldt E, Knight DP, et al.. Spider Silk Proteins – Mechanical Property and Gene Sequence. *Zoological Science*. 2005; doi: 10.2108/zsj.22.273.

127. Li X, Shi C-H, Tang C-L, Cai Y-M, Meng Q. The correlation between the length of repetitive domain and mechanical properties of the recombinant flagelliform spidroin. *Biology Open*. 2017; doi: 10.1242/bio.022665.

128. Yoshido A, Šíchová J, Pospíšilová K, Nguyen P, Voleníková A, Šafář J, et al.. Evolution of multiple sex-chromosomes associated with dynamic genome reshuffling in Leptidea wood-white butterflies. *Heredity*. 2020; doi: 10.1038/s41437-020-0325-9.

129. Rens W, O'Brien PC, Grutzner F, Clarke O, Graphodatskaya D, Tsend-Ayush E, et al.. The multiple sex chromosomes of platypus and echidna are not completely identical and several share homology with the avian Z. *Genome Biol*. 2007; doi: 10.1186/gb-2007-8-11-r243.

130. Andrews S. FastQC: A quality control tool for high throughput sequencing. 2010;

131. Krueger F, James F, Ewels P, Afyounian E, Schuster-Boeckler B. FelixKrueger/TrimGalore: v0.6.7 - DOI via Zenodo. Zenodo;

132. Bolger AM, Lohse M, Usadel B. Trimmomatic: a flexible trimmer for Illumina sequence data. *Bioinformatics*. 2014; doi: 10.1093/bioinformatics/btu170.

133. Vurture GW, Sedlazeck FJ, Nattestad M, Underwood CJ, Fang H, Gurtowski J, et al.. GenomeScope: fast reference-free genome profiling from short reads. Berger B, editor. *Bioinformatics*. 2017; doi: 10.1093/bioinformatics/btx153.

134. Stothard P, Wishart DS. Circular genome visualization and exploration using CGView. *Bioinformatics*. 2005; doi: 10.1093/bioinformatics/bti054.

135. Seppey M, Manni M, Zdobnov EM. BUSCO: Assessing Genome Assembly and Annotation Completeness. In: Kollmar M, editor. *Gene Prediction*. New York, NY: Springer New York;

136. Simão FA, Waterhouse RM, Ioannidis P, Kriventseva EV, Zdobnov EM. BUSCO: assessing genome assembly and annotation completeness with single-copy orthologs. *Bioinformatics*. 2015; doi: 10.1093/bioinformatics/btv351.

137. Waterhouse RM, Seppey M, Simão FA, Manni M, Ioannidis P, Klioutchnikov G, et al.. BUSCO Applications from Quality Assessments to Gene Prediction and Phylogenomics. *Molecular Biology and Evolution*. 2018; doi: 10.1093/molbev/msx319.

138. Kriventseva EV, Kuznetsov D, Tegenfeldt F, Manni M, Dias R, Simão FA, et al.. OrthoDB v10: sampling the diversity of animal, plant, fungal, protist, bacterial and viral genomes for evolutionary and functional annotations of orthologs. *Nucleic Acids Research*. 2019; doi: 10.1093/nar/gky1053.

139. Gurevich A, Saveliev V, Vyahhi N, Tesler G. QUAST: quality assessment tool for genome assemblies. *Bioinformatics*. 2013; doi: 10.1093/bioinformatics/btt086.

140. Kim D, Paggi JM, Park C, Bennett C, Salzberg SL. Graph-based genome alignment and genotyping with HISAT2 and HISAT-genotype. *Nat Biotechnol*. 2019; doi: 10.1038/s41587-019-0201-4.

141. The UniProt Consortium. UniProt: a worldwide hub of protein knowledge. *Nucleic Acids Research*. 2019; doi: 10.1093/nar/gky1049.

142. Tarailo-Graovac M, Chen N. Using RepeatMasker to Identify Repetitive Elements in Genomic Sequences. *Current Protocols in Bioinformatics*. 2009; doi: 10.1002/0471250953.bi0410s25.

143. Storer J, Hubley R, Rosen J, Wheeler TJ, Smit AF. The Dfam community resource of transposable element families, sequence models, and genome annotations. *Mobile DNA*. 2021; doi: 10.1186/s13100-020-00230-y.

144. Lomsadze A. Gene identification in novel eukaryotic genomes by self-training algorithm. *Nucleic Acids Research*. 2005; doi: 10.1093/nar/gki937.

145. Lomsadze A, Burns PD, Borodovsky M. Integration of mapped RNA-Seq reads into automatic training of eukaryotic gene finding algorithm. *Nucleic Acids Research*. 2014; doi: 10.1093/nar/gku557.

146. Stanke M, Schöffmann O, Morgenstern B, Waack S. Gene prediction in eukaryotes with a generalized hidden Markov model that uses hints from external sources. *BMC Bioinformatics*. 2006; doi: 10.1186/1471-2105-7-62.

147. Stanke M, Diekhans M, Baertsch R, Haussler D. Using native and syntenically mapped cDNA alignments to improve de novo gene finding. *Bioinformatics*. 2008; doi: 10.1093/bioinformatics/btn013.

148. Gotoh O. A space-efficient and accurate method for mapping and aligning cDNA sequences onto genomic sequence. *Nucleic Acids Research*. 2008; doi: 10.1093/nar/gkn105.

149. Li H, Handsaker B, Wysoker A, Fennell T, Ruan J, Homer N, et al.. The Sequence Alignment/Map format and SAMtools. *Bioinformatics*. 2009; doi: 10.1093/bioinformatics/btp352.

150. Barnett DW, Garrison EK, Quinlan AR, Stromberg MP, Marth GT. BamTools: a C++ API and toolkit for analyzing and managing BAM files. *Bioinformatics*. 2011; doi: 10.1093/bioinformatics/btr174.

151. Iwata H, Gotoh O. Benchmarking spliced alignment programs including Spaln2, an extended version of Spaln that incorporates additional species-specific features. *Nucleic Acids Research*. 2012; doi: 10.1093/nar/gks708.

152. Buchfink B, Xie C, Huson DH. Fast and sensitive protein alignment using DIAMOND. *Nat Methods*. 2015; doi: 10.1038/nmeth.3176.

153. Hoff KJ, Lomsadze A, Borodovsky M, Stanke M. Whole-Genome Annotation with BRAKER. In: Kollmar M, editor. *Gene Prediction*. New York, NY: Springer New York;

154. Brůna T, Lomsadze A, Borodovsky M. GeneMark-EP+: eukaryotic gene prediction with self-training in the space of genes and proteins. *NAR Genomics and Bioinformatics*. 2020; doi: 10.1093/nargab/lqaa026.

155. Rice P, Longden I, Bleasby A. EMBOSS: The European Molecular Biology Open Software Suite. *Trends in Genetics*. 2000; doi: 10.1016/S0168-9525(00)02024-2.

156. Ashburner M, Ball CA, Blake JA, Botstein D, Butler H, Cherry JM, et al.. Gene Ontology: tool for the unification of biology. *Nat Genet*. 2000; doi: 10.1038/75556.

157. The Gene Ontology Consortium, Carbon S, Douglass E, Good BM, Unni DR, Harris NL, et al.. The Gene Ontology resource: enriching a GOld mine. *Nucleic Acids Research*. 2021; doi: 10.1093/nar/gkaa1113.

158. Caspi R, Billington R, Ferrer L, Foerster H, Fulcher CA, Keseler IM, et al.. The MetaCyc database of metabolic pathways and enzymes and the BioCyc collection of pathway/genome databases. *Nucleic Acids Res*. 2016; doi: 10.1093/nar/gkv1164.

159. Caspi R, Billington R, Fulcher CA, Keseler IM, Kothari A, Krummenacker M, et al.. The MetaCyc database of metabolic pathways and enzymes. *Nucleic Acids Research*. 2018; doi: 10.1093/nar/gkx935.

160. Gillespie M, Jassal B, Stephan R, Milacic M, Rothfels K, Senff-Ribeiro A, et al.. The reactome pathway knowledgebase 2022. *Nucleic Acids Research*. 2022; doi: 10.1093/nar/gkab1028.

161. Jassal B, Matthews L, Viteri G, Gong C, Lorente P, Fabregat A, et al.. The reactome pathway knowledgebase. *Nucleic Acids Research*. 2019; doi: 10.1093/nar/gkz1031.

162. Lu S, Wang J, Chitsaz F, Derbyshire MK, Geer RC, Gonzales NR, et al.. CDD/SPARCLE: the conserved domain database in 2020. *Nucleic Acids Research*. 2020; doi: 10.1093/nar/gkz991.

163. Lewis TE, Sillitoe I, Dawson N, Lam SD, Clarke T, Lee D, et al.. Gene3D: Extensive prediction of globular domains in proteins. *Nucleic Acids Research*. 2018; doi: 10.1093/nar/gkx1069.

164. Pedruzzi I, Rivoire C, Auchincloss AH, Coudert E, Keller G, de Castro E, et al.. HAMAP in 2015: updates to the protein family classification and annotation system. *Nucleic Acids Research*. 2015; doi: 10.1093/nar/gku1002.

165. Necci M, Piovesan D, Dosztányi Z, Tosatto SCE. MobiDB-lite: Fast and highly specific consensus prediction of intrinsic disorder in proteins. *Bioinformatics*. 2017; doi: 10.1093/bioinformatics/btx015.

166. Mi H, Muruganujan A, Huang X, Ebert D, Mills C, Guo X, et al.. Protocol Update for large-scale genome and gene function analysis with the PANTHER classification system (v.14.0). *Nat Protoc*. 2019; doi: 10.1038/s41596-019-0128-8.

167. Wu CH. PIRSF: family classification system at the Protein Information Resource. *Nucleic Acids Research*. 2004; doi: 10.1093/nar/gkh097.

168. Chen C, Wang Q, Huang H, Vinayaka CR, Garavelli JS, Arighi CN, et al.. PIRSitePredict for protein functional site prediction using position-specific rules. *Database*. 2019; doi: 10.1093/database/baz026.

169. Attwood TK. PRINTS and its automatic supplement, prePRINTS. *Nucleic Acids Research*. 2003; doi: 10.1093/nar/gkg030.

170. Sigrist CJA. PROSITE: A documented database using patterns and profiles as motif descriptors. *Briefings in Bioinformatics*. 2002; doi: 10.1093/bib/3.3.265.

171. Sigrist CJA, de Castro E, Cerutti L, Cuche BA, Hulo N, Bridge A, et al.. New and continuing developments at PROSITE. *Nucleic Acids Research*. 2012; doi: 10.1093/nar/gks1067.

172. Akiva E, Brown S, Almonacid DE, Barber AE, Custer AF, Hicks MA, et al.. The Structure–Function Linkage Database. *Nucl Acids Res*. 2014; doi: 10.1093/nar/gkt1130.

173. Letunic I, Bork P. 20 years of the SMART protein domain annotation resource. *Nucleic Acids Research*. 2018; doi: 10.1093/nar/gkx922.

174. Letunic I, Khedkar S, Bork P. SMART: recent updates, new developments and status in 2020. *Nucleic Acids Research*. 2021; doi: 10.1093/nar/gkaa937.

175. Pandurangan AP, Stahlhacke J, Oates ME, Smithers B, Gough J. The SUPERFAMILY 2.0 database: a significant proteome update and a new webserver. *Nucleic Acids Research*. 2019; doi: 10.1093/nar/gky1130.

176. Gough J, Karplus K, Hughey R, Chothia C. Assignment of homology to genome sequences using a library of hidden Markov models that represent all proteins of known structure. *Journal of Molecular Biology*. 2001; doi: 10.1006/jmbi.2001.5080.

177. Haft DH, Selengut JD, Richter RA, Harkins D, Basu MK, Beck E. TIGRFAMs and Genome Properties in 2013. *Nucleic Acids Research*. 2012; doi: 10.1093/nar/gks1234.

178. Selengut JD, Haft DH, Davidsen T, Ganapathy A, Gwinn-Giglio M, Nelson WC, et al.. TIGRFAMs and Genome Properties: tools for the assignment of molecular function and biological process in prokaryotic genomes. *Nucleic Acids Research*. 2007; doi: 10.1093/nar/gkl1043.

179. Haft DH. The TIGRFAMs database of protein families. *Nucleic Acids Research*. 2003; doi: 10.1093/nar/gkg128.

180. Haft DH. TIGRFAMs: a protein family resource for the functional identification of proteins. *Nucleic Acids Research*. 2001; doi: 10.1093/nar/29.1.41.

181. Buels R, Yao E, Diesh CM, Hayes RD, Munoz-Torres M, Helt G, et al.. JBrowse: a dynamic web platform for genome visualization and analysis. *Genome Biol*. 2016; doi: 10.1186/s13059-016-0924-1.

182. Li H. Minimap2: pairwise alignment for nucleotide sequences. Birol I, editor. *Bioinformatics*. 2018; doi: 10.1093/bioinformatics/bty191.

183. Li H. New strategies to improve minimap2 alignment accuracy. *arXiv:210803515 [q-bio]*. 2021;

184. Gasteiger E. ExPASy: the proteomics server for in-depth protein knowledge and analysis. *Nucleic Acids Research*. 2003; doi: 10.1093/nar/gkg563.

185. Kyte J, Doolittle RF. A simple method for displaying the hydropathic character of a protein. *Journal of Molecular Biology*. 1982; doi: 10.1016/0022-2836(82)90515-0.

186. Buchan DWA, Minneci F, Nugent TCO, Bryson K, Jones DT. Scalable web services for the PSIPRED Protein Analysis Workbench. *Nucleic Acids Research*. 2013; doi: 10.1093/nar/gkt381.

187. Teufel F, Almagro Armenteros JJ, Johansen AR, Gíslason MH, Pihl SI, Tsirigos KD, et al.. SignalP 6.0 predicts all five types of signal peptides using protein language models. *Nat Biotechnol*. 2022; doi: 10.1038/s41587-021-01156-3.

188. Roberson EJ, Chips MJ, Carson WP, Rooney TP. Deer herbivory reduces web-building spider abundance by simplifying forest vegetation structure. *PeerJ*. 2016; doi: 10.7717/peerj.2538.

189. Glatz L. Zur biologie und morphologie von Oecobius annulipes lucas (Araneae, Oecobiidae). *Z Morph Tiere*. 1967; doi: 10.1007/BF00400986.

Figure 1

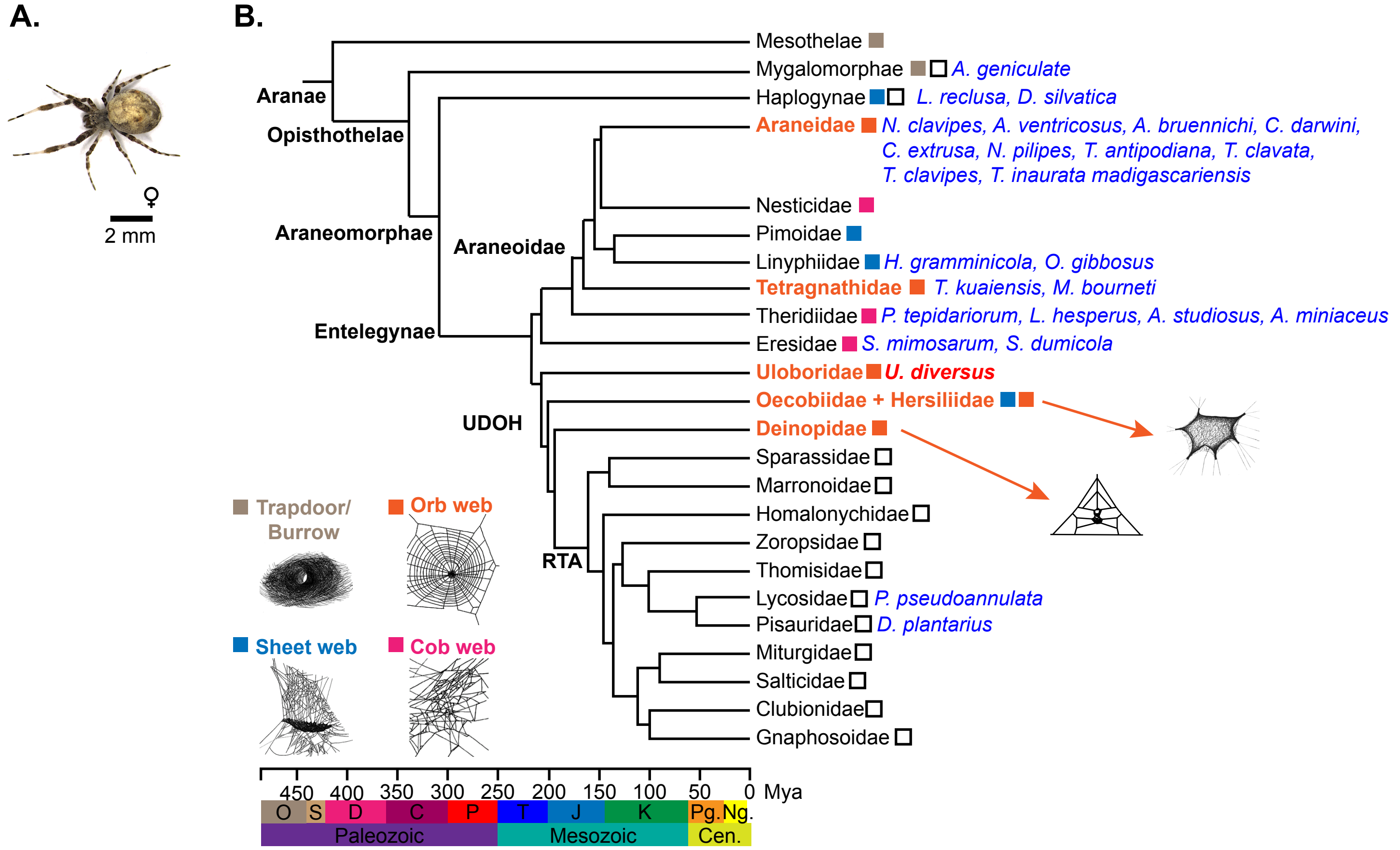
A.
B.
♀
2 mm
Aranae
Opisthothelae
Araneomorphae
Araneoidae
Entelegynae
UDOH
RTA
Mesothelae
Mygalomorphae A. geniculate
Haplogynae L. reclusa, D. silvatica
Araneidae N. clavipes, A. ventricosus, A. bruennichi, C. darwini, C. extrusa, N. pilipes, T. antipodiana, T. clavata, T. clavipes, T. inaurata madigascariensis
Nesticidae
Pimoidae
Linyphiidae H. gramminicola, O. gibbosus
Tetragnathidae T. kuaiensis, M. bourneti
Theridiidae P. tepidariorum, L. hesperus, A. studiosus, A. miniaceus
Eresidae S. mimosarum, S. dumicola
Uloboridae U. diversus
Oecobiidae + Hersiliidae
Deinopidae
Sparassidae
Marronoidae
Homalonychidae
Zoropsidae
Thomisidae
Lycosidae P. pseudoannulata
Pisauridae D. plantarius
Miturgidae
Salticidae
Clubionidae
Gnaphosoidae
Trapdoor/ Burrow
Orb web
Sheet web
Cob web
450 400 350 300 250 200 150 100 50 0 Mya
O S D C P T J K Pg. Ng.
Paleozoic
Mesozoic
Cen.

Figure 2

Click here to access/download;Figure;Figure_2_220613.pdf

Figure 2

A.

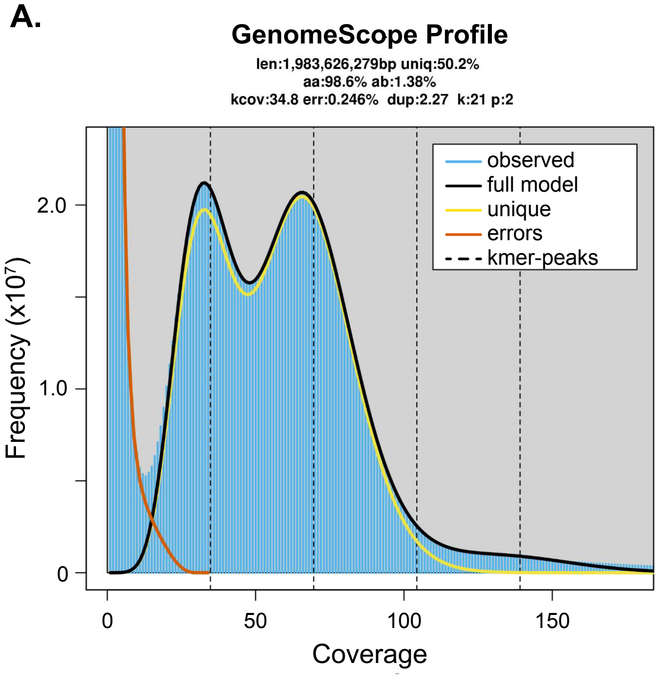

Figure 3
Click here to access/download;Figure;Figure_3_220510.pdf

Figure 3

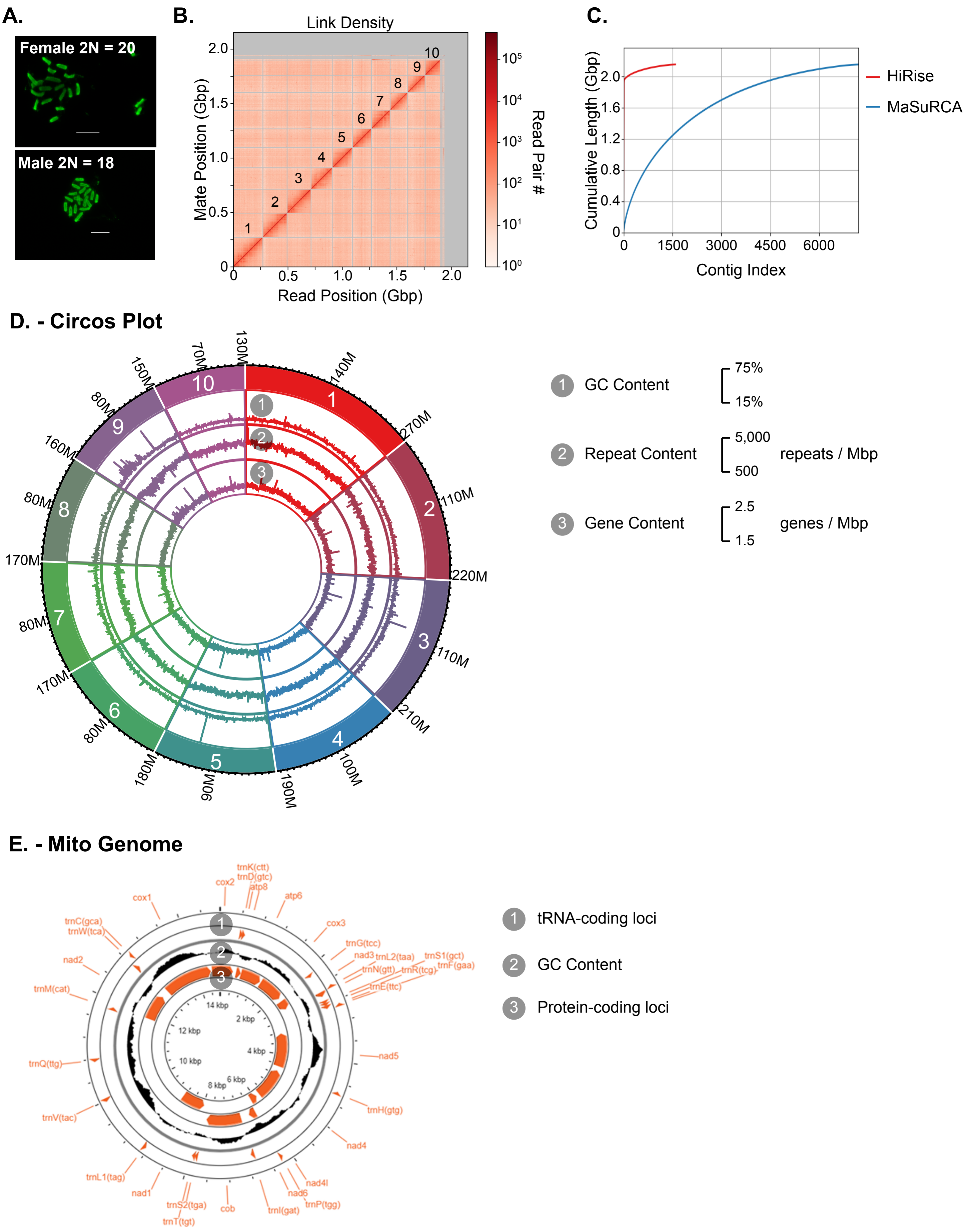

Figure 4 Click here to access/download;Figure;Figure_4_220425.pdf

Figure 4

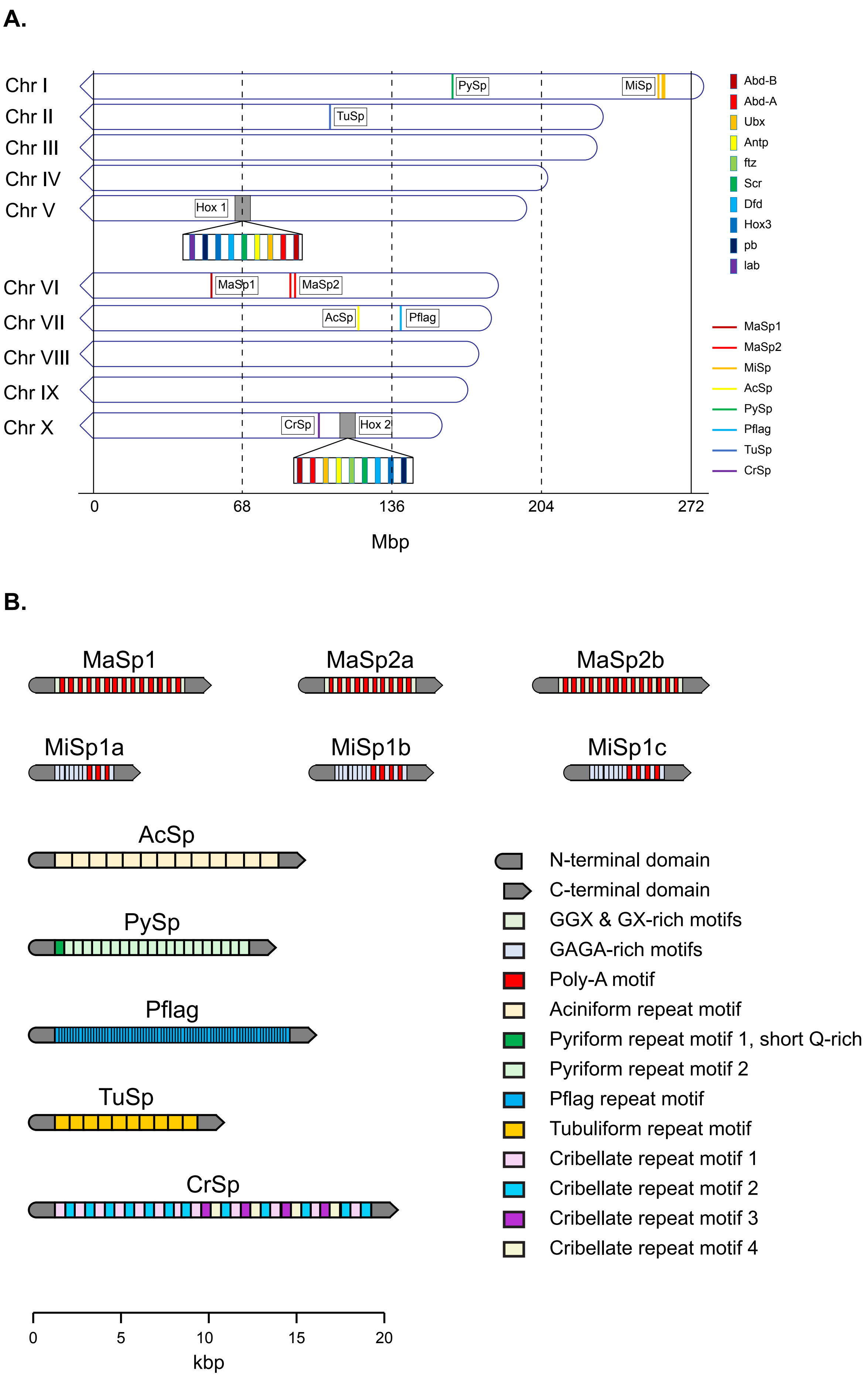

Figure 5 Click here to access/download;Figure;Figure_5_220628.pdf

Figure 5

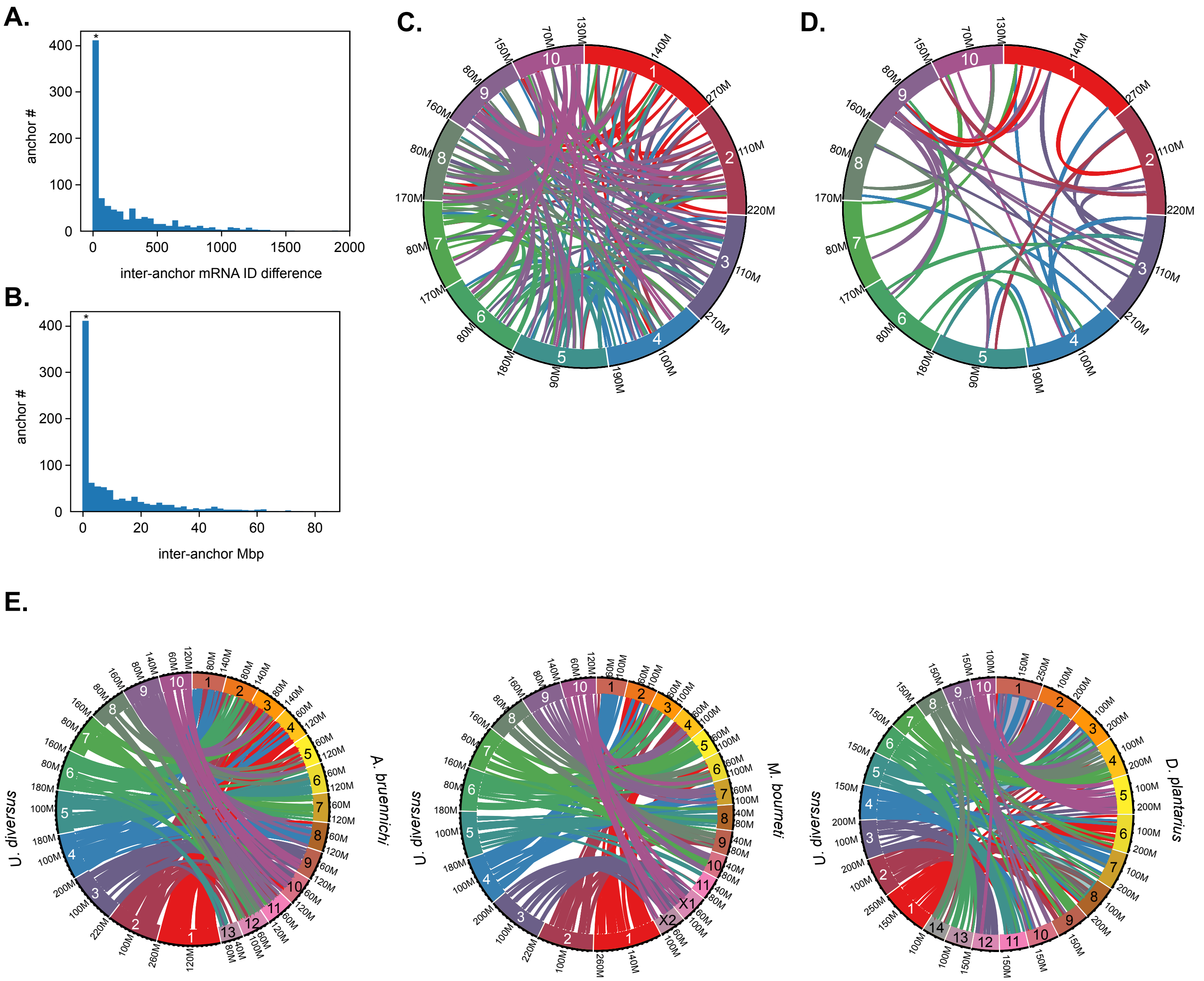

Figure 6

Click here to access/download;Figure;Figure_6_220617.pdf

Figure 6

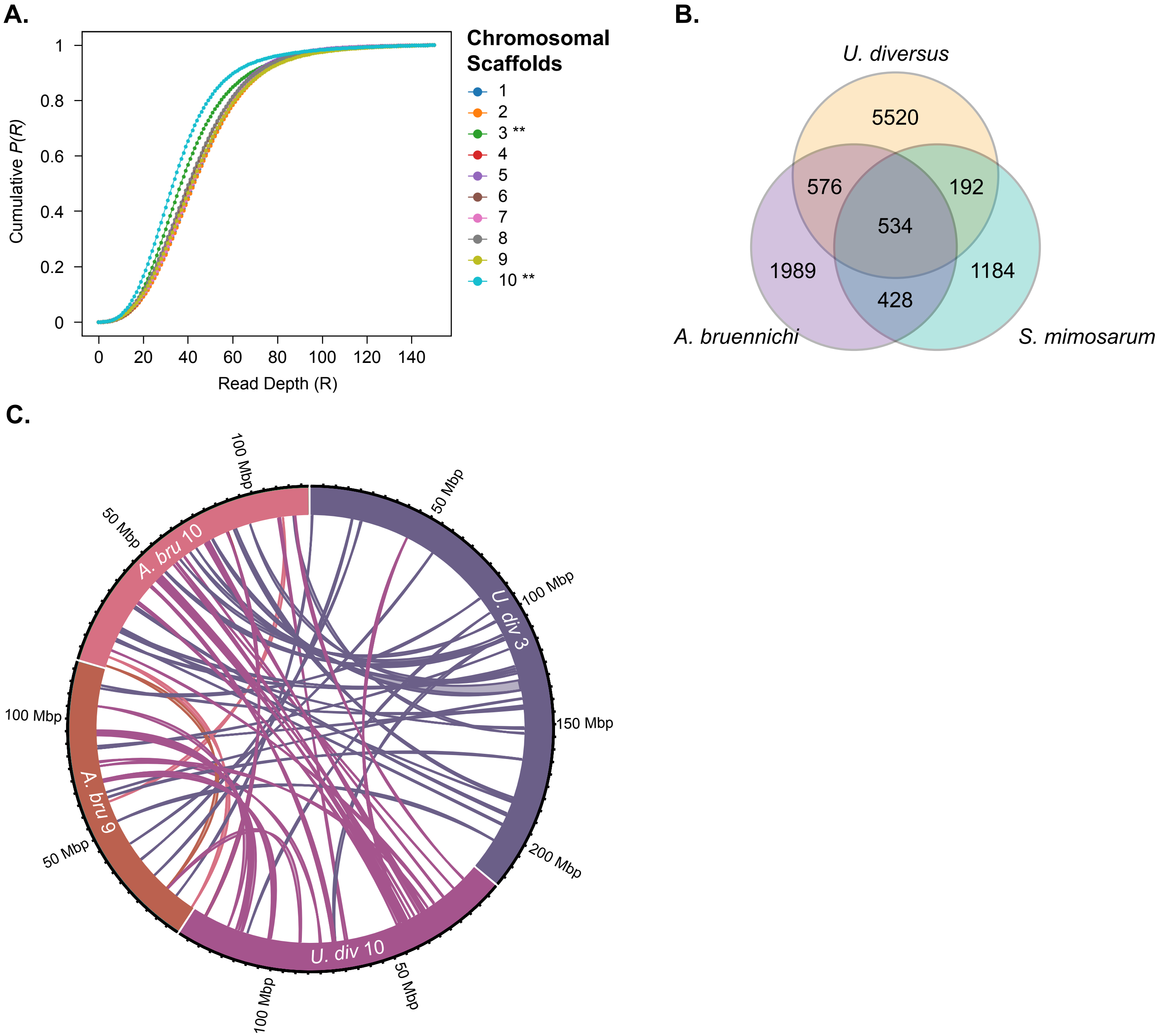

Click here to access/download
Supplementary Material
Table S1.xlsx

Click here to access/download
Supplementary Material
Table S2.xlsx

Click here to access/download
Supplementary Material
Table S3.txt

Click here to access/download
Supplementary Material
Table S4.xlsx

Click here to access/download
Supplementary Material
Table S5.xlsx

Click here to access/download
Supplementary Material
Table S6.xlsx

Personal Cover Click here to access/download;Personal Cover;Miller_Cover - 20220628.pdf

Andrew G. Gordus, Ph. D.
Assistant Professor
Department of Biology
Johns Hopkins University
Biology East 207
3400 N. Charles St.
Baltimore, MD 21218
Office: 410-516-6509
Email: agordus@jhu.edu

June 28, 2022
To the editors of *GigaScience*:

This letter accompanies our manuscript entitled "**Chromosome-level genome and the identification of sex chromosomes in Uloborus diversus**."

In this paper, we provide a chromosome-level assembly of the genome for *Uloborus diversus*, an orb-weaving spider. The origins of orb-weaving is hotly debated, since two distantly related branches of spider families (Araneoid and UDOH) perform this behavior, yet most spiders that are monophyletic with these two branches lack orb-weaving. This has led to disagreement over whether orb-weaving evolved once and was lost multiple times, or else independently evolved several times. While several Araneoid genomes have been sequenced, no genomes from the UDOH group have been assembled, hampering efforts to pursue a genetic understanding of this behavior. Nearly half of all spider species are within the UDOH+RTA clade, though very few genomes exist for these species, in part due to the difficulty in assembling spider genomes.

The primary advancements / findings of our paper are the following:

- **A chromosome-level assembly of an important spider genome**. Spider genomes are notoriously difficult to assemble due to their large size and high heterogeneity, which is compounded by the need to often use several individuals for sufficient DNA. Here we outline an approach for a chromosome-level assembly leveraging several sequencing technologies using only 5 small spiders (<20 mg/spider).
- **Synteny analysis showing that reshuffling of chromosomes has occurred since an ancient genome duplication**. Prior work with spider genomes has posited that the common ancestor of spiders and scorpion experienced a whole genome duplication. However, considerable chromosomal reshuffling has occurred. This is observed in mammalian genomes as well and has been posited as a source of genetic diversity that enabled mammals to successfully colonize a diversity of habitats.
- **Complete sequences for all spidroin families in the genome**. Due to their size (coding regions >10 kbp) and high repeat content, spidroins are notoriously difficult to sequence, let alone a complete catalog from a single species. Here we present a complete catalog from *U. diversus*, which is the only species to have its complete catalog of spidroins sequenced.
- **The identification of sex-chromosomes, and candidate sex-determining genes**. Spiders use a multi-sex chromosome system for sex determination, but a genetic understanding of how sex determination occurs is unknown, in part because of the absence of identified sex chromosomes. We identified sex chromosomes in U. diversus, and leveraged this information along with sex-associated scaffolds in other spiders to narrow down the pool of candidate sex-determining genes to 534.

We believe this makes a significant advance in spider genomics, especially with regard to sex-determination and providing a full catalog of full-length spidroins from a single species. The assembly approach we used leveraging several sequencing technologies to address the challenges of assembling a large genome from small, heterogeneic animals should be of broad interest to readers of *GigaScience* who are interested in genome assembly approaches.

All sequencing and assembly data have been deposited in the BioProject PRJNA846873. All gene and animal names adhere to approved nomenclature.

As potential reviewers, we can suggest Jonathan Coddington (Smithsonian), Damian Elias (UC Berkeley), Alistair McGregor (Oxford University). We are aware of a competing group who are also assembling a *U. diversus* genome, and request the following individuals NOT be reviewers: Sandra Correa-Garhwal, Cheryl Hayashi, Rick Baker, Nadia Ayoub, Thomas Clarke, Brent Opell, John Gatesy, Jessica Garb, Joe Arguelles, Nhen Hunter, Thomas Dugger.

We thank you for considering our manuscript for publication, and look forward to hearing from you soon.

Sincerely,

Andrew Gordus and Jeremiah Miller, on behalf of the authors
