# Supplementary material for: Chromosome-level genome and the identification of sex chromosomes in Uloborus diversus
Source: Gigascience. 2023 Feb 10;12:giad002. doi: 10.1093/gigascience/giad002 (PMC9912274; doi:10.1093/gigascience/giad002)
Supplement: giad002_Response_to_Reviewer_Comments_Original_Submission [file giad002_response_to_reviewer_comments_original_submission.pdf]

Author's Response To Reviewer Comments

Close

RESPONSE TO REVIEWERS

We appreciate the helpful comments from the Reviewers, which have improved the accuracy and clarity of the manuscript. W

We address the specific points raised by the Reviewers below:

Reviewer #1

This paper presents the first uloborid spider genome--and it is a chromosome level assembly. Genomes of this family are imp

We thank the reviewer for their enthusiasm and insightful suggestions. We have made suggested changes, as outlined below

Figure 1
A. geniculate -- spelling
N. clavipes = T. clavipes

Table S1
Number of Componenet Sequences-- typo

Thank you for catching these errors. They have been corrected in Figure 1 and Table S1.

Text
single exon We found a -- typo

Thank you for drawing our attention to this typo. The correct punctuation has been added on line 460.

can be ascribed by -- can be inferred by?

Thank you for this suggestion. This introductory sentence has been rewritten to provide clearer language on line 478. In doin

an Araneid orb-weaver-- araneid usually not capitalized

We appreciate this correction. The change has been made to the text on line 508. In addition, we identified three additional c

♂X1X2/♀X1X1X2X2.[48] should be ♂X1X2/♀X1X1X2X2 [48].

Thank you for catching this. We have made the correction to the punctuation on line 526, addressing the placement of the re

You might want to be careful about citing Purcell & Pruitt, see
https://nam02.safelinks.protection.outlook.com/?url=https%3A%2F%2Fpurcelllab.ucr.edu%2Fblog6.html&data=05%7C01%
JXVCI6Mn0%3D%7C3000%7C%7C%7C&sdata=H7Cicwwwc%2BkWKxxf7WpuqMtgiupNfnWKLF%2FefeTsyzM%3D&reserved=
and other questions about Pruitt's work.

Thank you so much for advising us of the troublesome nature of this work. We have removed the reference and any associat

Re methods, it would be of interest to know what HMW DNA fragment sizes were (expressed as kb, or mb), although Tape St

Thank you for bringing our attention to this omission. A statement regarding the average integrated percent of mass account

Any homologs of the Pyriform spidroin (PySp) in Acanthoscurria? Piriform silk attachment points are a synapomorphy of aran
of web architectures in non-araneomorph spiders. Therefore finding homologs to PySp spidroins in non-araneomorph spiders

Thank you, yes this is an excellent point. We initially held off on too much spidroin analysis since we were aware of another r
on this on lines 450 to 455, and have cited their paper.

Likewise, tubuliform spidroin (TuSp) is probably a synapomorphy of entelegyne spiders, with derived female genitalia--a "flow
is not clear. Any thoughts on this?

Thank you, this is an interesting point about entelegyne spiders. We can only speculate, but compared to mygalamorphs whic
may have evolved to provide enhanced protection for the eggs relative to mygalamorph egg sacs which rely on the burrow fo

It is good to see attention paid to the mitochondrial genome, as many whole genome studies ignore it. In spiders, early work
Evolution, Volume 21, Issue 5, May 2004, Pages 893-902. Any comments on U. diversus tRNAs from that point of view?

Thank you for the suggestion. In the original text we did state the tRNAs we identified also lacked the 3' aminoacyl acceptor

Finally, any comments on evidence for or against the convergent evolution of the orb web? Homology between the pseudofla
include a sentence in intro acknowledging this, but saying that this paper intends to present the genome and address sex chr

Thank you, yes we were vague on this point, and added text to address this in the spidroin and discussion sections (Lines 33

Reviewer #2

In this study, the authors generated huge genome sequencing data and RNA-seq data and provided a genome assembly with
to be addressed.
1. There are huge sequencing data from different samples. However, I don't think that marge of different assemblies is good
compared with the merged one, but obviously much better in avoiding redundancy. I strongly suggest that the author adopt

Thank you, we apologize for the confusion on this point. The final assembly is ultimately based on the single-individual PacBi
1.5% of sequence added to the HIFI assembly. The small amount of sequence added from the Illumina+ONT assembly impro

We have added language to clarify the process by which the SAMBA tool improves the PacBio IPA assembly, removing the mi

2. Proportion of repeats are somewhat affected by the quality of assembly. The high heterozygous genome assembly is comp

Thank you, and again we apologize for the confusion. On lines 190 to 198 we described the statistics of the assembly. We ag
valuable as the basis for an understanding of the genetics and evolution of hundreds of plants, animals, and other species. W
is consistent with that predicted by GenomeScope

3. About the assembly of RNA-seq data. The authors get huge amounts of data. However, it is not so helpful to obtain novel

We agree that de novo assembly of short-read Illumina RNA-seq data is an error-prone process, and the data were generated
from RNA-seq data guided by the genome assembly. Trinity is quite capable of assembling full length transcripts with the hel

4. As to whole genome duplication. The authors did not provided solid evidence supporting that WGD occurred in U. diversus
statements.

We apologize for this, and have downplayed our statements. Our observations are consistent with prior work on WGD. We in
domestic plants[3, 4] or humans and chimpanzees[5], duplications or fusion events can be observed based on large syntenic

5. The identification of the sex chromosome is still vague. The statements are not well organized. The statements and the res
rather convictive. As I know the authors sequenced female and male samples. So why they didn't clearly compare the depth

Thank you, we apologize for the poor communication and added additional sentences to clarify the analyses. As you thoughtf
consistent with what was observed in A. bruennichi. We provide 3 possible reasons for why we might observe this discrepanc
chromosome 5 and 10, the read depth disparity between males and females would be 75% for reads that map to this region

However, we think the sum total of read depth and synteny analysis are all consistent with chromosomes 3 and 10 being the

Other:
1. The information of chromosome-level spider genome are not Incomplete. As I know, there is a black widow genome with c

Thank you for bringing this discrepancy to our attention. We have added this reference and cited this genome and included th

2. The authors need to release the sequences of the spidroins the identified and described.

Thank you for the suggestion. While we intended to release these sequences in the public databases as soon as possible follo

Reviewer #3

The manuscript GIGA-D-22-00169 presents a chromosome-level genome of the cribellate orb-weaving spider Uloborus divers
candidate sex-determining genes.

The methods of work are well fitted to the aims of the study, clearly described, and well written.

Thank you for your kind comments.

Minor comments:

1. In the Figure 1B, I noticed that it noted the estimated divergence times of the Araneae, I think there should be add the ref

Thank you for bringing this to our attention. We have added the appropriate reference to the Figure 1B Legend on lines 998 a

2. There is something wrong with the table format, such as Table1, 2, 5 and Table 6

Thank you for pointing this out. Tables 2 and 4 have been removed from their in-line placement in the text and will be submi

3. Line 70: "chromosome- scale" changes to "chromosome-scale".

Thank you for pointing out this typographical error. The errant space has been removed on line 73.

4. Line 147 to lines 148: Line breaks error.

Thank you for catching this formatting error. The inappropriate carriage return has been removed on line 159.

5. Line 458: "[48]" in the wrong location.

Thank you for catching this. We have made the correction to the punctuation on line 526, addressing the placement of the re

6. Line 511-512: In the genome of spider Uloborus diversus, which chromosome the genes of "sex lethal (sxl)" and "doublese

We apologize for the misunderstanding here. As we stated in the text, sex-lethal is the insect sensor for chromosome dosage
but there is no reason to expect them to be on the X chromosomes. In flies, doublesex is not on the X chromosome (it's on c
involved in sex-determination would involve considerable work. Even then, there would be no expectation to find it on the X-

However, for chromosome-based sex determination, a chromosome dosage sensor is necessary to determine the embryo's zy
in different animal phyla[7]. However, our knowledge of this is limited to nematodes, insects, and placental mammals, and in
research.

7. Line 515-516: "The 534 shared sex-linked genes in these three species, 14 are predicted to be DNA/RNA-binding", if these

Thank you, this is a great question. Unfortunately, we do not have sufficient sample numbers or read depths for the single sa
purposes of this publication, we focused on obtaining a sufficient transcriptome assembly to assist in identifying gene models
the protein expression disparity between sexes. Alternatively, the chromosome dosage sensor may not be a protein-encoding

8. Line 685: "Dovetail Chicago and Dovetail Hi-C Sequencing" should be bold.

Thank you for your attention to this detail. We have changed the formatting from italic to bold for this subsection heading on

9. Line 764: "We then used the Trinity assembler43 v.2.12.0", the number of 43 may be redundancy.

Thank you for catching this typo. We have removed the number 43 from line 873.

10. Some softwares lack the number of RRID, such as line 223 "BRAKER2", line 245 of "NOVOplasty", line 790 of "tRNAscan-

Thank you for noticing this. We have added RRIDs for every software with an available RRID at this time. There are two softw

11. Lines 780 "using the BRAKER 2 pipeline" changes to "using the BRAKER2 pipeline.

Thank you for pointing this error out. We have removed the space indicated on line 891.

12. Lines 950: "Literature Cited" changes to "Reference".

We appreciate this suggestion. "Literature Cited" has been changed to "References" on line 1076.

13. Lines 952-953: wrong cite. The World Spider Catalog is a web online, the version and the data you accessed from should

Thank you for catching this error. The appropriate changes have been made to address this error in the reference formatting.

References

1. Correa-Garhwal SM, Baker RH, Clarke TH, Ayoub NA, Hayashi CY. The evolutionary history of cribellate orb-weaver capture

2. Haas BJ, Papanicolaou A, Yassour M, Grabherr M, Blood PD, Bowden J, et al. De novo transcript sequence reconstruction fr

3. Daccord N, Celton JM, Linsmith G, Becker C, Choisne N, et al. High-quality de novo assemlby of the apple genome and me

4. Qiao X, Yin H, Li L, Wang R, Wu J, Wu J, and Zhang S. Different modes of gene duplication show divergent evolutionary pa

5. de Pontbraind A, Wang X-P, Cavaloc Y, Mattei M-G, and Galibert F. Synteny comparison between apes and human using fi

6. Waters PD, Patel HR, Ruiz-Herrera A, Álvarez-González L, Lister NC, Simakov O, et al. Microchromosomes are building bloc

7. Ercan S. Mechanisms of x chromosome dosage compensation. J. Genomics. 2015; doi: 10.7150/jgen.10404

Close
