# Supplementary material for: Chromosome-level genome and the identification of sex chromosomes in Uloborus diversus
Source: Gigascience. 2023 Feb 10;12:giad002. doi: 10.1093/gigascience/giad002 (PMC9912274; doi:10.1093/gigascience/giad002)
Supplement: giad002_Reviewer_1_Report_Original_Submission — Jonathan Coddington -- 8/10/2022 [file giad002_reviewer_1_report_original_submission.pdf]

Reviewer Report

Title: Chromosome-level genome and the identification of sex chromosomes in Uloborus diversus.

Version: Original Submission **Date:** 8/10/2022

Reviewer name: Jonathan Coddington

Reviewer Comments to Author:

This paper presents the first uloborid spider genome--and it is a chromosome level assembly. Genomes of this family are important because the orb web is supposedly independently and convergently evolved in this group. Although my expertise is not in the technology and informatics of genome sequencing, it appears to be well done.
Figure 1
A. geniculate -- spelling
N. clavipes = T. clavipes
Table S1
Number of Componenet Sequences-- typo
Text
single exon We found a -- typo
can be ascribed by -- can be inferred by?
an Araneid orb-weaver-- araneid usually not capitalized
â™,X1X2/â™€X1X1X2X2.[48] should be â™,X1X2/â™€X1X1X2X2 [48].
You might want to be careful about citing Purcell & Pruitt, see
https://purcelllab.ucr.edu/blog6.html
and other questions about Pruitt's work.
Re methods, it would be of interest to know what HMW DNA fragment sizes were (expressed as kb, or mb), although Tape Stations are not very accurate. For people who collect spiders with the intent to yield HMW DNA, such data are important. Data are scarce, so any facts are significant.
Any homologs of the Pyriform spidroin (PySp) in Acanthoscurria? Piriform silk attachment points are a synapomorphy of araneomorph or "true" spiders. Liphistiomorph and mygalomorph spiders do not (cannot?) make point attachments, and the inability to make point attachments either to substrate or silk-silk point attachments probably constrains/ed the evolution of web architectures in non-araneomorph spiders. Therefore finding homologs to PySp spidroins in non-araneomorph spiders is of great interest to explain araneomorph web architecture diversity.
Likewise, tubuliform spidroin (TuSp) is probably a synapomorphy of entelegyne spiders, with derived female genitalia--a "flow-though" sperm management system. Eggsacs occur widely in non-entelegyne spiders, so it is a mystery why entelegynes have specialized spigots, glands, and spidroins for the same purpose. Indeed, the particular function of tubuliform silk is not clear. Any thoughts on this? E.g.
It is good to see attention paid to the mitochondrial genome, as many whole genome studies ignore it. In spiders, early work claimed that tRNA's appeared to be peculiar. Masta and Boore. 2004. The Complete Mitochondrial Genome Sequence of the Spider Habronattus oregonensis Reveals Rearranged

and Extremely Truncated tRNAs. Molecular Biology and Evolution, Volume 21, Issue 5, May 2004, Pages 893-902. Any comments on U. diversus tRNAs from that point of view?
Finally, any comments on evidence for or against the convergent evolution of the orb web? Homology between the pseudoflagelliform and flagelliform spidroins would be pertinent. The intro does raise expectations that some of the macro / larger evolutionary questions will be addressed in the paper, but many, see above, are only cursory or not too much. Perhaps include a sentence in intro acknowledging this, but saying that this paper intends to present the genome and address sex chromosomes, but other topics? For example the sections on some of the spidroins do not extensively discuss comparisons with other spider genomes.

Methods

Are the methods appropriate to the aims of the study, are they well described, and are necessary controls included? Choose an item.

Conclusions

Are the conclusions adequately supported by the data shown? Choose an item.

Reporting Standards

Does the manuscript adhere to the journal's guidelines on minimum standards of reporting? Choose an item.

Choose an item.

Statistics

Are you able to assess all statistics in the manuscript, including the appropriateness of statistical tests used? Choose an item.

Quality of Written English

Please indicate the quality of language in the manuscript: Choose an item.

Declaration of Competing Interests

Please complete a declaration of competing interests, considering the following questions:

- Have you in the past five years received reimbursements, fees, funding, or salary from an organisation that may in any way gain or lose financially from the publication of this manuscript, either now or in the future?
- Do you hold any stocks or shares in an organisation that may in any way gain or lose financially from the publication of this manuscript, either now or in the future?

- Do you hold or are you currently applying for any patents relating to the content of the manuscript?
- Have you received reimbursements, fees, funding, or salary from an organization that holds or has applied for patents relating to the content of the manuscript?
- Do you have any other financial competing interests?
- Do you have any non-financial competing interests in relation to this paper?

If you can answer no to all of the above, write 'I declare that I have no competing interests' below. If your reply is yes to any, please give details below.

I declare that I have no competing interests

I agree to the open peer review policy of the journal. I understand that my name will be included on my report to the authors and, if the manuscript is accepted for publication, my named report including any attachments I upload will be posted on the website along with the authors' responses. I agree for my report to be made available under an Open Access Creative Commons CC-BY license (http://creativecommons.org/licenses/by/4.0/). I understand that any comments which I do not wish to be included in my named report can be included as confidential comments to the editors, which will not be published.

Choose an item.

To further support our reviewers, we have joined with Publons, where you can gain additional credit to further highlight your hard work (see: https://publons.com/journal/530/gigascience). On publication of this paper, your review will be automatically added to Publons, you can then choose whether or not to claim your Publons credit. I understand this statement.

Yes Choose an item.
