# Supplementary material for: Chromosome-level genome and the identification of sex chromosomes in Uloborus diversus
Source: Gigascience. 2023 Feb 10;12:giad002. doi: 10.1093/gigascience/giad002 (PMC9912274; doi:10.1093/gigascience/giad002)
Supplement: giad002_Reviewer_2_Report_Original_Submission — Hui Xiang, PH.D -- 8/13/2022 [file giad002_reviewer_2_report_original_submission.pdf]

Reviewer Report

Title: Chromosome-level genome and the identification of sex chromosomes in Uloborus diversus.

Version: Original Submission **Date:** 8/13/2022

Reviewer name: Hui Xiang, PH.D

Reviewer Comments to Author:

In this study, the authors generated huge genome sequencing data and RNA-seq data and provided a genome assembly with rather complicated merging approach, of a spider with novel phylogenetic position. The genome undoubtedly added novel and important resources for deep understanding of spider evolution. However, there are still severe issues that need to be addressed.

1. There are huge sequencing data from different samples. However, I don't think that marge of different assemblies is good for a final qualified genome. Given high heterozygosity, that illumina data and ONT data from different individuals is quite difficult to use for assembling a clean genome. As shown in Table 2, assembly by Hify approach is not obviously inferior compared with the merged one, but obviously much better in avoiding redundancy. I strongly suggest that the author adopt the genome assembly of Hify data from one individual, instead of merging two sets of assemblies. Illumina and Nanopore assembly may be helpful in fully deciphering silk proteins.
2. Proportion of repeats are somewhat affected by the quality of assembly. The high heterozygous genome assembly is complicated merged by diverse batch of data, so the real quality might be not as good as the author described. The quality of repeat is especially hard to evaluate. Hence the statements on genome size (Line 193-200) are not convictive.
3. About the assembly of RNA-seq data. The authors get huge amounts of data. However, it is not so helpful to obtain novel transcripts if the data is saturated. More importantly, assembly of short reads is even not so useful to obtain long transcripts.
4. As to whole genome duplication. The authors did not provided solid evidence supporting that WGD occurred in U. diversus genome. They only demonstrated two hox clusters therein. The synteny analysis was quite confusing which is not helpful in confirmation of WGD. They need to provide more solid genome-wide evidence, or otherwise totally downplay the statements.
5. The identification of the sex chromosome is still vague. The statements are not well organized. The statements and the results are so vague and not convictive. "While 8 of the 10 pseudochromsomes had a median read depth of 40 Â± 2, pseudochromosomes 3 and 10 were outliers, with read depths of 36 and 33, respectively." The difference in sequencing depth is rather convictive. As I know the authors sequenced female and male samples. So why they didn't clearly compare the depth of the two sex chromosomes between them and make more evidence?

Other:

1. The information of chromosome-level spider genome are not Incomplete. As I know, there is a black widow genome with chromosome-level. The authors need to added this one.
2. The authors need to release the sequences of the spidroins the identified and described.

Methods

Are the methods appropriate to the aims of the study, are they well described, and are necessary controls included? Choose an item.

Conclusions

Are the conclusions adequately supported by the data shown? Choose an item.

Reporting Standards

Does the manuscript adhere to the journal's guidelines on minimum standards of reporting? Choose an item.

Choose an item.

Statistics

Are you able to assess all statistics in the manuscript, including the appropriateness of statistical tests used? Choose an item.

Quality of Written English

Please indicate the quality of language in the manuscript: Choose an item.

Declaration of Competing Interests

Please complete a declaration of competing interests, considering the following questions:

- Have you in the past five years received reimbursements, fees, funding, or salary from an organisation that may in any way gain or lose financially from the publication of this manuscript, either now or in the future?
- Do you hold any stocks or shares in an organisation that may in any way gain or lose financially from the publication of this manuscript, either now or in the future?
- Do you hold or are you currently applying for any patents relating to the content of the manuscript?
- Have you received reimbursements, fees, funding, or salary from an organization that holds or has applied for patents relating to the content of the manuscript?
- Do you have any other financial competing interests?
- Do you have any non-financial competing interests in relation to this paper?

If you can answer no to all of the above, write 'I declare that I have no competing interests' below. If your reply is yes to any, please give details below.

I declare that I have no competing interests.

I agree to the open peer review policy of the journal. I understand that my name will be included on my report to the authors and, if the manuscript is accepted for publication, my named report including any attachments I upload will be posted on the website along with the authors' responses. I agree for my report to be made available under an Open Access Creative Commons CC-BY license (http://creativecommons.org/licenses/by/4.0/). I understand that any comments which I do not wish to be included in my named report can be included as confidential comments to the editors, which will not be published.

Choose an item.

To further support our reviewers, we have joined with Publons, where you can gain additional credit to further highlight your hard work (see: https://publons.com/journal/530/gigascience). On publication of this paper, your review will be automatically added to Publons, you can then choose whether or not to claim your Publons credit. I understand this statement.

Yes Choose an item.
