# Supplementary material for: Chromosome-level genome and the identification of sex chromosomes in Uloborus diversus
Source: Gigascience. 2023 Feb 10;12:giad002. doi: 10.1093/gigascience/giad002 (PMC9912274; doi:10.1093/gigascience/giad002)
Supplement: giad002_Reviewer_3_Report_Original_Submission — Zhisheng Zhang, Ph.D -- 8/20/2022 [file giad002_reviewer_3_report_original_submission.pdf]

Reviewer Report

Title: Chromosome-level genome and the identification of sex chromosomes in Uloborus diversus.

Version: Original Submission **Date:** 8/20/2022

Reviewer name: Zhisheng Zhang, Ph.D

Reviewer Comments to Author:

The manuscript GIGA-D-22-00169 presents a chromosome-level genome of the cribellate orb-weaving spider Uloborus diversus. The assembly reinforces evidence of an ancient arachnid genome duplication and identifies complete open reading frames for every class of spidroin gene. And the authors identified the two X chromosomes for U. diversus and identify candidate sex-determining genes.
The methods of work are well fited to the aims of the study, clearly described, and well written.
Minor comments:

1. In the Figure 1B, I noticed that it noted the estimated divergence times of the Araneae, I think there should be add the reference, or detail describe how to do.
2. There is something wrong with the table format, such as Table1, 2, 5 and Table 6.
3. Line 70: "chromosome- scale" changes to "chromosome-scale".
4. Line 147 to lines 148: Line breaks error.
5. Line 458: "[48]" in the wrong location.
6. Line 511-512: In the genome of spider Uloborus diversus, which chromosome the genes of "sex lethal (sxl)" and "doublesex (dsx)" located at?
7. Line 515-516: "The 534 shared sex-linked genes in these three species, 14 are predicted to be DNA/RNA-binding", if these sex-linked genes have difference on RNA level between male and female?
8. Line 685: "Dovetail Chicago and Dovetail Hi-C Sequencing" should be bold.
9. Line 764: "We then used the Trinity assembler43 v.2.12.0", the number of 43 may be redundancy.
10. Some softwares lack the number of RRID, such as line 223 "BRAKER2", line 245 of "NOVOplasty", line 790 of "tRNAscan-SE", line 773 of "RepeatModeler", line 774 of "RepeatMasker", line 797 of "EMBOSS", and so on.
11. Lines 780 "using the BRAKER 2 pipeline" changes to "using the BRAKER2 pipeline".
12. Lines 950: "Literature Cited" changes to "Reference".
13. Lines 952-953: wrong cite. The World Spider Catalog is a web online, the version and the data you accessed from should also added, and the author's name should change to World Spider Catalog.

Methods

Are the methods appropriate to the aims of the study, are they well described, and are necessary controls included? Choose an item.

Conclusions

Are the conclusions adequately supported by the data shown? Choose an item.

Reporting Standards

Does the manuscript adhere to the journal's guidelines on minimum standards of reporting? Choose an item.

Choose an item.

Statistics

Are you able to assess all statistics in the manuscript, including the appropriateness of statistical tests used? Choose an item.

Quality of Written English

Please indicate the quality of language in the manuscript: Choose an item.

Declaration of Competing Interests

Please complete a declaration of competing interests, considering the following questions:

- Have you in the past five years received reimbursements, fees, funding, or salary from an organisation that may in any way gain or lose financially from the publication of this manuscript, either now or in the future?
- Do you hold any stocks or shares in an organisation that may in any way gain or lose financially from the publication of this manuscript, either now or in the future?
- Do you hold or are you currently applying for any patents relating to the content of the manuscript?
- Have you received reimbursements, fees, funding, or salary from an organization that holds or has applied for patents relating to the content of the manuscript?
- Do you have any other financial competing interests?
- Do you have any non-financial competing interests in relation to this paper?

If you can answer no to all of the above, write 'I declare that I have no competing interests' below. If your reply is yes to any, please give details below.

I declare that I have no competing interests

I agree to the open peer review policy of the journal. I understand that my name will be included on my report to the authors and, if the manuscript is accepted for publication, my named report including any attachments I upload will be posted on the website along with the authors' responses. I agree for my report to be made available under an Open Access Creative Commons CC-BY license (http://creativecommons.org/licenses/by/4.0/). I understand that any comments which I do not wish to

be included in my named report can be included as confidential comments to the editors, which will not be published.

Choose an item.

To further support our reviewers, we have joined with Publons, where you can gain additional credit to further highlight your hard work (see: https://publons.com/journal/530/gigascience). On publication of this paper, your review will be automatically added to Publons, you can then choose whether or not to claim your Publons credit. I understand this statement.

Yes Choose an item.
